# Association of armed conflict and global measles cases: A structural equation modeling analysis of 193 countries from 2000 to 2023

**DOI:** 10.1371/journal.pmed.1004819

**Published:** 2026-06-25

**Authors:** Tyler Y. Headley, Yesim Tozan

**Affiliations:** 1 Department of War Studies, King’s College London, London, United Kingdom; 2 Department of Global and Environmental Health, School of Global Public Health, New York University, New York, New York, United States of America; The Hospital for Sick Children, CANADA

## Abstract

**Background:**

Global armed conflict and population displacement are increasing, yet their association with population health remains poorly understood. We developed and tested four theoretical models linking armed conflict, population displacement, and socioeconomic development to measles burden across 193 countries from 2000 to 2023.

**Methods and findings:**

We analyzed longitudinal country-level data comprising 4,632 country-year observations, combining fixed-effects panel regression and structural equation modeling (SEM). Observed variables included battle-related deaths (BRDs) and forcibly displaced population sizes, while socioeconomic development was modeled as a latent variable incorporating gross domestic product (GDP) per capita, life expectancy, and mean years of schooling. Outcomes were total measles cases and incidence per million population. All four constructed models demonstrated excellent fit (Comparative Fit Index [CFI] 0.991–0.996; Tucker–Lewis Index [TLI] 0.976–0.989; Root Mean Square Error of Approximation [RMSEA] 0.046–0.062). Higher contemporaneous BRDs were associated with higher measles cases (β = 0.17; 95% Confidence Interval [CI] [0.14, 0.20]; *p* < 0.001), adjusting for population displacement and economic development. When prior-year BRDs were included as an observed variable, the direct effect of contemporaneous BRDs was no longer significant (β = 0.05; 95% CI [−0.01, 0.11]; *p* = 0.091), while the effect of prior-year BRDs was significantly associated with measles cases (β = 0.14; 95% CI [0.08, 0.20]; *p* < 0.001) but not incidence after accounting for displacement (β = 0.04; 95% CI [–0.02, 0.11]; *p* = 0.164). Each standard deviation (SD) increase in a country’s standardized log-transformed BRDs was associated with an approximately 0.20 SD increase in measles cases, equivalent to 2,500 additional reported cases for every 3,700 BRDs. In all models, BRDs had a slight negative association with socioeconomic development (β = −0.10; 95% CI [−0.13, −0.07]; *p* < 0.001), and each SD increase in displaced population corresponded with a 0.20 SD decline in socioeconomic development (95% CI [−0.23, −0.17]; *p* < 0.001), which was the primary pathway by which displacement was associated with measles. Socioeconomic development, in turn, had a significant direct association with both measles cases and incidence, with each SD increase in socioeconomic development corresponding to a 0.32 to 0.34 and 0.34 to 0.36 SD reduction in cases and incidence, respectively (*p* < 0.001). Key limitations include reliance on national-level annual aggregates, possible under-reporting of both battle-related deaths and measles cases, and the possibility of unmeasured time-varying confounders that preclude causal interpretation.

**Conclusions:**

Armed conflict is associated with an increased measles burden, both directly and indirectly through associations with lower socioeconomic development and greater population displacement. These findings suggest that mitigating infectious disease risks in volatile settings requires a dual strategy: preserving the structural foundations of health and education while systematically integrating displaced populations into routine immunization programs. Future research using subnational and higher-frequency data is needed to clarify the precise mechanisms and timing of these associations across other vaccine-preventable diseases.

## Author summary

**Why was this study done?:**

- Armed conflict and forced displacement are rising globally, yet we have a limited understanding of the direct and indirect associations between conflict and the burden of preventable infectious diseases like measles.
- Previous research has focused on individual countries or short timeframes, leaving major gaps in understanding how conflict is associated with disease outbreaks through weakened health systems, economic decline, or mass population displacement.
- We designed this study to examine these interconnected pathways across 193 countries over more than two decades.

**What did the researchers do and find?:**

- We combined data on battle-related deaths (BRDs), forced displacement, economic development, and measles cases from 2000 to 2023, using statistical models that can test multiple pathways simultaneously.
- We found that conflict was positively associated with measles cases, with this association persisting 1 year after fighting. This suggests that the association between conflict and health outcomes endures beyond the period of active violence.
- The strongest pathway ran through socioeconomic decline: conflict and displacement were associated with lower levels of economic and broader socioeconomic development, and these structural losses were in turn substantially associated with higher measles burden.

**What do these findings mean?:**

- Reducing measles outbreaks in conflict-affected settings requires more than emergency vaccination drives: it also requires protecting health systems and economies that underpin long-term population health and health outcomes.
- Displaced populations must be systematically included in routine immunization programs, as their exclusion creates pockets of vulnerability that can drive outbreaks.
- These results are subject to three main limitations: our reliance on national aggregate data may obscure local heterogeneities; BRDs and measles cases may be undercounted, especially in the most conflict-affected areas; and we cannot rule out the impact of unobserved factors, such as political instability or reduced humanitarian access, on the associations described.

## Abstract 3

In a structural equation modelling analysis, Tyler Headley and Yesim Tozan investigate the association between armed conflict, population displacement, socioeconomic development, and measles burden across 193 countries over more than two decades.

## Introduction

Global armed conflict and forcibly displaced populations are increasing worldwide [1,2], yet their empirical association with vaccine-preventable disease (VPD) outcomes remains poorly understood. Measles, a highly contagious VPD, continues to cause substantial morbidity and mortality, particularly in low-resource settings where vaccination coverage is incomplete [3]. Conflict has been hypothesized to be associated with greater measles burden not only through direct disruptions to immunization services [4], but also indirectly via population displacement, economic decline, and weakened health systems [5,6]. Understanding these interconnected pathways is critical for informing timely humanitarian response and global health policy.

Although prior research has linked armed conflict to outbreaks of infectious diseases, most studies have focused on direct effects and are limited to single-country analyses or short timeframes [5]. Few have quantified the indirect or mediating pathways linking conflict to population health outcomes, or examined the direct and indirect pathways in tandem, leaving unresolved questions about the mechanisms of disruption and the relative influence of each on infectious disease burden [7,8]. These underexplored pathways are important as economic decline and educational disruption occurring in conflict settings may be associated with reduced population resilience, while weakened health infrastructure and interrupted vaccination programs may further increase disease risk [5–7].

To address these gaps in the empirical evidence base, we developed and tested four theoretical models of the direct and indirect pathways that linked armed conflict, population displacement, and socioeconomic development to measles cases and incidence. To do so, we used data from across 193 countries from 2000 to 2023 and employed fixed-effects panel regressions and SEM to test and compare our models and hypotheses. We used SEM as a theory-informed, exploratory framework to characterize the relative strength and interdependence of multiple hypothesized pathways linking armed conflict, displacement, socioeconomic conditions, and measles burden over time, rather than to identify causal effects. More robust causal inference approaches were not feasible given the absence of well-defined treatment onset, substantial cross-national heterogeneity, and the likelihood of reciprocal and time-varying processes that limit credible causal identification in global country-year data. By employing this methodological framework, our study provides a global perspective on how armed conflict is associated with infectious disease outcomes and informs strategies to mitigate public health impacts in affected populations.

## Methods

This study employed country-level longitudinal panel data from 193 countries and applied statistical methods, including fixed-effects panel regression and SEM, to examine the direct and indirect pathways linking armed conflict and measles cases and incidence. The analytical period was restricted to 2000–2023, the years for which consistent data were available for all variables across all sources. This study is reported as per the Strengthening the Reporting of Observational Studies in Epidemiology (STROBE) guidelines (S1 Checklist). The hypotheses, primary Structural equation models (SEMs) (Models A–D), and fixed-effects regression strategy were specified before data analysis began as part of the project’s conceptual development, although no formal prospective protocol document was registered. The robustness analyses presented in the Supporting Information were planned in part prospectively and in part in response to peer-review comments. Analyses added in response to peer-review, and therefore identified as data-driven, comprise: the IHS transformations (S5 Table); the first-order AR [1] specifications (S7 Table); the cluster-robust standard errors (S9 Table); the first-difference SEM (S10 Table); the extended distributed-lag analysis (S11 Table); the parallel mediation analysis (S12 Table); and the hierarchical decomposition of total effects (S13 Table). These additions did not alter the substantive conclusions of the primary analyses.

### Hypotheses and analytical plan

We tested three a priori hypotheses: (H1) higher annual battle-related deaths (BRDs) within a country are associated with greater contemporaneous measles burden (cases and incidence per million population), adjusting for population displacement and socioeconomic development; (H2) higher 1-year-lagged BRDs are associated with a greater contemporaneous measles burden, capturing delayed disruption of health and immunization systems; and (H3) the association between armed conflict and measles burden is partly mediated by indirect pathways operating through population displacement and a latent construct of socioeconomic development. The prespecified analytical strategy to test these hypotheses comprised (i) descriptive summaries of all variables; (ii) fixed-effects panel regressions (Models 1–20, Table 2) to characterize the bivariate and progressively adjusted associations between conflict, displacement, socioeconomic indicators, and measles cases; and (iii) four prespecified SEMs (Models A–D, Table 3, and Fig 2) to estimate direct and indirect path coefficients within a single coherent system. The analyses performed correspond to this plan with the additions noted above (S5, S9, S10, S11, S12, and S13 Tables) introduced as supplementary robustness analyses in response to peer-review comments. Where reported analyses differ from the prespecified plan, this is identified in the relevant subsection of the Results, Robustness analysis subsection, or footnotes to the corresponding table or figure.

**Table 2:** Fixed-effects panel regression estimates showing the associations between battle-related deaths, population displacement, socioeconomic development, vaccination coverage, and measles cases, 2000–2023.

Predictors					
	BRDs	BRDs (lagged)	Population displacement	GDP per Capita	Mean Vaccination Coverage
**Predictors of GDP per Capita (logged, standardized)**					
*Model 1*	−0.02 (−0.04, 0) *p* = 0.044				
*Model 2*		−0.03 (−0.04, −0.01) *p* = 0.002			
*Model 3*	−0.01 (−0.02, 0.01) *p* = 0.474		−0.03 (−0.05, −0.02) *p* < 0.001		
*Model 4*	0 (−0.01, 0.02) *p* = 0.609		−0.02 (−0.03, 0) *p* = 0.025		0.14 (0.12, 0.16) *p* < 0.001
*Model 5*	0.01 (−0.01, 0.04) *p* = 0.151	−0.02 (−0.04, 0) *p* = 0.129	−0.02 (−0.03, 0) *p* = 0.041		0.12 (0.1, 0.14) *p* < 0.001
**Predictors of Displacement (standardized)**					
*Model 6*	0.35 (0.32, 0.39) *p* < 0.001				
*Model 7*		0.44 (0.4, 0.47) *p* < 0.001			
*Model 8*	0.35 (0.31, 0.39) *p* < 0.001			−0.13 (−0.2, −0.07) *p* < 0.001	
*Model 9*	0.33 (0.3, 0.37) *p* < 0.001			−0.07 (−0.14, −0.01) *p* = 0.025	−0.16 (−0.2, −0.12) *p* < 0.001
*Model 10*	0.15 (0.1, 0.19) *p* < 0.001	0.33 (0.29, 0.38) *p* < 0.001		−0.07 (−0.13, 0) *p* = 0.041	−0.14 (−0.18, −0.1) *p* < 0.001
**Predictors of BRDs (logged, standardized)**					
*Model 11*		0.60 (0.58, 0.62) *p* < 0.001			
*Model 12*			0.21 (0.19, 0.24) *p* < 0.001		
*Model 13*		0.55 (0.53, 0.58) *p* < 0.001	0.08 (0.06, 0.1) *p* < 0.001	0.02 (−0.03, 0.06) *p* = 0.514	
*Model 14*		0.55 (0.52, 0.57) *p* < 0.001	0.08 (0.05, 0.1) *p* < 0.001	0.03 (−0.01, 0.08) *p* = 0.151	−0.06 (−0.09, −0.03) *p* < 0.001
**Predictors of Measles Cases (logged, standardized)**					
*Model 15*	0 (−0.03, 0.04) *p* = 0.787				
*Model 16*		0.02 (−0.01, 0.06) *p* = 0.183			
*Model 17*	0 (−0.03, 0.04) *p* = 0.891		0.01 (−0.02, 0.03) *p* = 0.666		
*Model 18*	0 (−0.04, 0.03) *p* = 0.824		0 (−0.03, 0.03) *p* = 0.884	−0.42 (−0.48, −0.36) *p* < 0.001	
*Model 19*	−0.01 (−0.05, 0.02) *p* = 0.488		−0.01 (−0.04, 0.02) *p* = 0.439	−0.39 (−0.45, −0.33) *p* < 0.001	−0.1 (−0.14, −0.06) *p* < 0.001
*Model 20*	−0.03 (−0.08, 0.01) *p* = 0.125	0.03 (−0.01, 0.07) *p* = 0.193	−0.02 (−0.05, 0.01) *p* = 0.179	−0.34 (−0.41, −0.28) *p* < 0.001	−0.1 (−0.14, −0.06) *p* < 0.001

Notes: All models include country and year fixed-effects with cluster-robust standard errors (clustered at the country-level). Dependent variables (DV) by block: Models 1–5, gross domestic product (GDP) per capita (log, standardized); Models 6–10, population displacement (% population, standardized); Models 11–14, battle-related deaths (log, standardized); Models 15–20, measles cases (log, standardized). The first model in each block (Models 1, 6, 11, and 15) presents the unadjusted bivariate association between the primary exposure and outcome; subsequent models within each block progressively add covariates as listed in the column headers and are therefore adjusted estimates. Adjustment variables across the full table comprise: country fixed-effects, year fixed-effects, contemporaneous and 1-year-lagged battle-related deaths (logged, standardized), population displacement (% of population, standardized), GDP per capita (logged, standardized), and mean vaccination coverage across 10 vaccines (standardized). Values represent coefficients with 95% confidence intervals (CIs) and two-sided *p*-values from Wald tests rounded to three decimal places. Abbreviations: BRDs = Battle-related deaths (logged); GDP = gross domestic product.

**Table 3:** Structural equation modeling (SEM) results including standardized coefficients with 95% confidence intervals of the theoretical models of the association between armed conflict, population displacement, socioeconomic development, and measles outcomes (Models A–D), 2000–2023.

Effect	Path type	Model A	Model B	Model C	Model D
**Latent variable loading**					
GDP per capita → Socioeconomic status	Latent Loading	0.94 [0.93, 0.95] *p* < 0.001	0.94 [0.93, 0.95] *p* < 0.001	0.94 [0.93, 0.94] *p* < 0.001	0.94 [0.93, 0.95] *p* < 0.001
Life expectancy → Socioeconomic status	Latent Loading	0.87 [0.86, 0.88] *p* < 0.001	0.87 [0.86, 0.88] *p* < 0.001	0.88 [0.87, 0.89] *p* < 0.001	0.87 [0.86, 0.89] *p* < 0.001
Mean years of schooling → Socioeconomic status	Latent Loading	0.83 [0.82, 0.84] *p* < 0.001	0.83 [0.82, 0.84] *p* < 0.001	0.83 [0.82, 0.85] *p* < 0.001	0.84 [0.83, 0.85] *p* < 0.001
**Predictors of socioeconomic status**					
Displacement (%) → Socioeconomic status	Indirect (Mediation)	−0.20 [−0.23, −0.17] *p* < 0.001	−0.20 [−0.23, −0.17] *p* < 0.001	−0.20 [−0.23, −0.17] *p* < 0.001	−0.20 [−0.23, −0.17] *p* < 0.001
BRDs → Socioeconomic status	Indirect (Mediation)	−0.10 [−0.13, −0.07] *p* < 0.001	−0.04 [−0.11, 0.03] *p* = 0.247	−0.10 [−0.13, −0.07] *p* < 0.001	−0.04 [−0.11, 0.03] *p* = 0.269
Prior-year BRDs → Socioeconomic status	Indirect (Mediation)	NA	−0.07 [−0.14, 0.01] *p* = 0.072	NA	−0.07 [−0.14, 0.01] *p* = 0.072
**Predictors of displacement**					
BRDs → Displacement (%)	Indirect (Mediation)	0.37 [0.32, 0.42] *p* < 0.001	0.13 [0.03, 0.24] *p* = 0.014	0.37 [0.32, 0.42] *p* < 0.001	0.13 [0.03, 0.24] *p* = 0.014
Prior-year BRDs → Displacement (%)	Indirect (Mediation)	NA	0.28 [0.17, 0.39] *p* < 0.001	NA	0.28 [0.17, 0.39] *p* < 0.001
**Predictors of BRDs**					
Prior-year BRDs → BRDs	Direct (Autoregressive)	NA	0.90 [0.88, 0.92] *p* < 0.001	NA	0.90 [0.88, 0.92] *p* < 0.001
**Predictors of measles cases (standardized)**					
Socioeconomic status → Measles cases (z)	Indirect (Mediation)	−0.34 [−0.37, −0.31] *p* < 0.001	−0.32 [−0.35, −0.30] *p* < 0.001	NA	NA
BRDs → Measles cases (z)	Direct	0.17 [0.14, 0.20] *p* < 0.001	0.05 [−0.01, 0.11] *p* = 0.091	NA	NA
Prior-year BRDs → Measles cases (z)	Direct	NA	0.14 [0.08, 0.20] *p* < 0.001	NA	NA
Displacement (%) → Measles cases (z)	Indirect (Mediation)	−0.01 [−0.04, 0.01] *p* = 0.229	−0.02 [−0.05, 0.00] *p* = 0.095	NA	NA
**Predictors of measles incidence (per 1M, standardized)**					
Socioeconomic status → Measles incidence (per 1M, z)	Indirect (Mediation)	NA	NA	−0.36 [−0.39, −0.33] *p* < 0.001	−0.34 [−0.37, −0.31] *p* < 0.001
BRDs → Measles incidence (per 1M, z)	Direct	NA	NA	0.03 [0.01, 0.06] *p* = 0.015	−0.00 [−0.07, 0.06] *p* = 0.906
Prior-year BRDs → Measles incidence (per 1M, z)	Direct	NA	NA	NA	0.04 [−0.02, 0.11] *p* = 0.164
Displacement (%) → Measles incidence (per 1M, z)	Indirect (Mediation)	NA	NA	0.06 [0.03, 0.10] *p* < 0.001	0.06 [0.03, 0.09] *p* < 0.001
**Model fit indices**					
Comparative Fit Index (CFI)		0.993	0.996	0.991	0.994
Tucker–Lewis Index (TLI)		0.983	0.989	0.976	0.984
Root Mean Square Error of Approximation (RMSEA)		0.052	0.046	0.062	0.056
Standardized Root Mean Square Residual (SRMR)		0.012	0.014	0.014	0.015
Akaike Information Criterion (AIC)		25,563.7	30,586.5	25,437.8	29,138.1
Bayesian Information Criterion (BIC)		25,686.0	30,746.4	25,560.2	29,298.1

Note: Structural equation models (SEMs) estimated standardized effects. Values represent standardized path coefficients with 95% confidence intervals in brackets and *p-values* rounded to three decimal places. Models A and B use standardized total measles cases as the outcome; Models C and D use standardized measles incidence per million population. Socioeconomic development is a latent construct defined by gross domestic product (GDP) per capita, life expectancy, and mean years of schooling. Within each model, the regression of each endogenous variable is adjusted for all of its directly antecedent variables in the path diagram. Specifically: (i) socioeconomic development (latent, indicated by GDP per capita, life expectancy, and mean years of schooling) is adjusted for contemporaneous BRDs and population displacement (and 1-year-lagged BRDs in Models B and D); (ii) population displacement is adjusted for contemporaneous BRDs (and lagged BRDs in Models B and D); (iii) the measles outcome (cases in Models A and B; incidence per million in Models C and D) is adjusted for socioeconomic development, population displacement, and contemporaneous BRDs (and lagged BRDs in Models B and D). Values represent standardized path coefficients with 95% CIs in brackets and two-sided *p*-values from Wald tests rounded to three decimal places. All models adjust for missing data using full information maximum likelihood (FIML) and use robust standard errors (maximum likelihood estimation with robust standard errors [MLR] estimator). Unadjusted bivariate associations between the primary exposure (BRDs) and outcomes (population displacement, GDP per capita, measles cases) are presented in Table 2 (Models 1, 6, 11, and 15). Table 3 reports the jointly estimated structural model in which all paths are simultaneously adjusted for the other variables in the system. Abbreviations: AIC = Akaike Information Criterion; BIC = Bayesian Information Criterion; BRDs = Battle-related deaths; CFI = Comparative Fit Index; GDP = gross domestic product; 1M = 1 million; RMSEA = Root Mean Square Error of Approximation; SRMR = Standardized Root Mean Square Residual; TLI = Tucker–Lewis Index.

**Fig 1:**
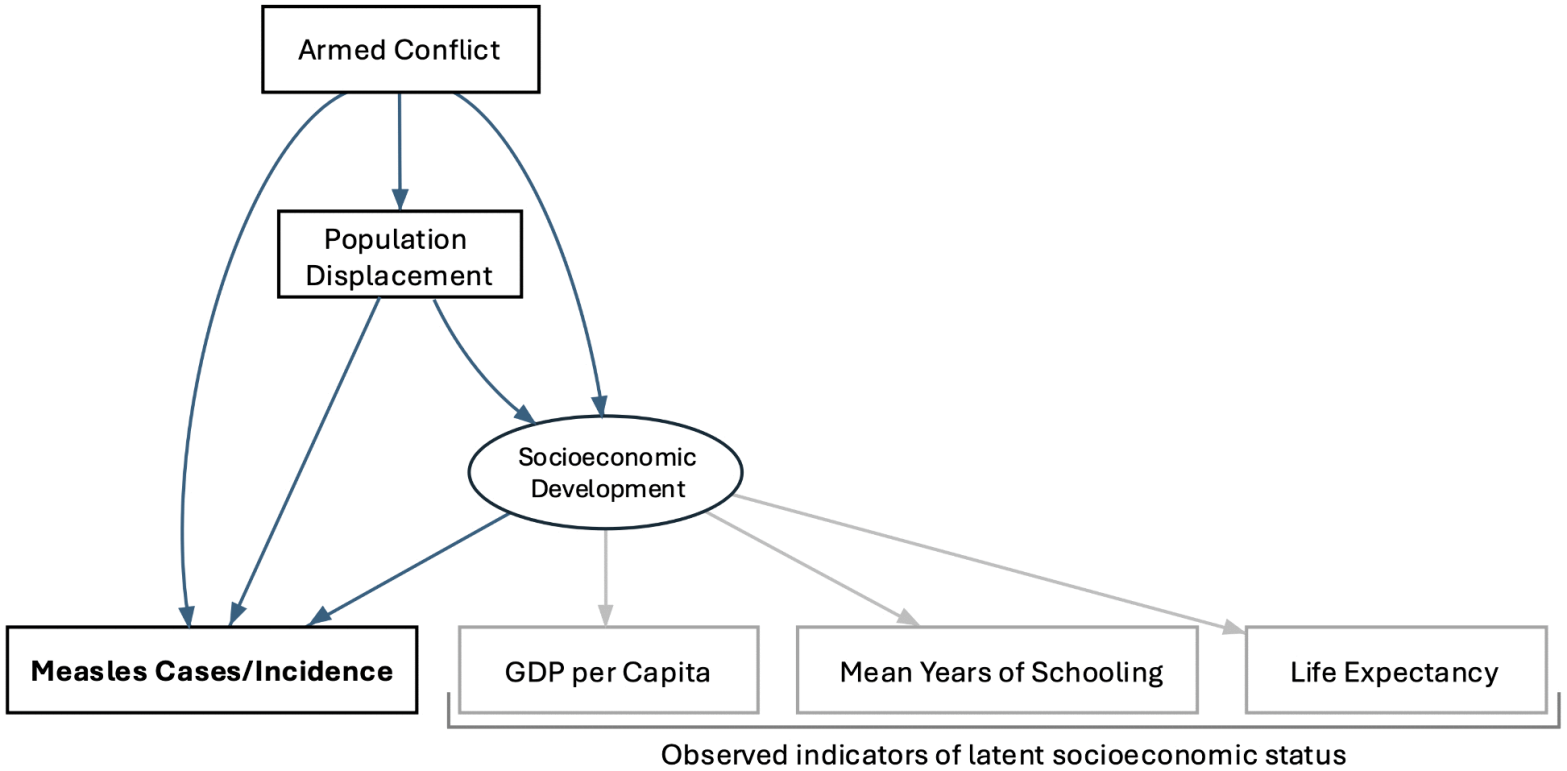
Theoretical pathways between armed conflict, population displacement, socioeconomic development, and measles cases and incidence.

**Fig 2:**
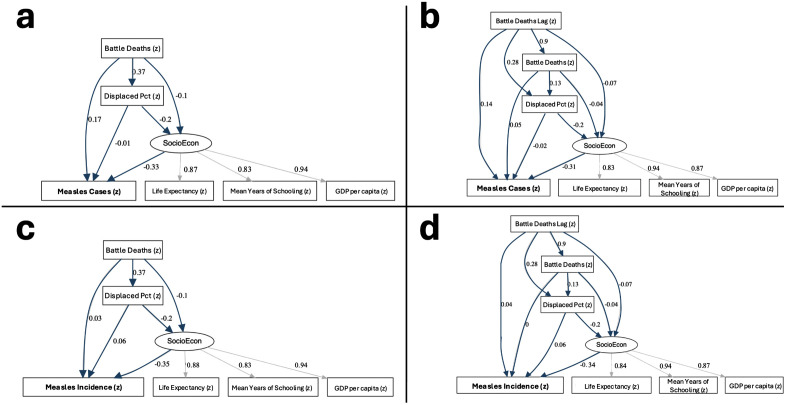
Structural equation models illustrating hypothesized pathways and standardized coefficients of the models linking armed conflict, population displacement, socioeconomic development, and measles outcomes. **Note**: panels **a–d** present structural equation models (SEMs) illustrating the hypothesized relationships among armed conflict (battle-related deaths), population displacement, socioeconomic development (life expectancy, mean years of schooling, and gross domestic product [GDP] per capita), and measles outcomes. Models A and C include only contemporaneous effects, using standardized measles cases and incidence per million population, respectively, while Models B and D extend these frameworks by incorporating 1-year lagged conflict exposure to capture delayed associations. In all models, the latent socioeconomic development construct links conflict and displacement to measles outcomes. Within each model, the regression of each endogenous variable is adjusted for all of its directly antecedent variables in the path diagram. Specifically: (i) socioeconomic development (latent, indicated by GDP per capita, life expectancy, and mean years of schooling) is adjusted for contemporaneous BRDs and population displacement (and 1-year-lagged BRDs in Models B and D); (ii) population displacement is adjusted for contemporaneous BRDs (and lagged BRDs in Models B and D); (iii) the measles outcome (cases in Models A and B; incidence per million in Models C and D) is adjusted for socioeconomic development, population displacement, and contemporaneous BRDs (and lagged BRDs in Models B and D). Values represent standardized path coefficients with 95% CIs in brackets and two-sided *p*-values from Wald tests rounded to three decimal places. Abbreviation: Socioecon = socioeconomic development.

### Data

Our dependent variables were reported measles cases and reported measles incidence (cases per 1,000,000 population), both sourced from the World Health Organization (WHO) [9]. These data are extracted annually through WHO/United Nations Children’s Fund (UNICEF) Joint Reporting Form on Immunization. Reported measles cases (standardized as described in the statistical analysis section) were the primary dependent variable, while reported measles incidence (similarly standardized) was used as an alternative outcome in our model specifications. For robustness checks, we used a secondary dataset on measles incidence from the Institute for Health Metrics and Evaluation (IHME) Global Burden of Disease (GBD) study as an alternative to WHO-sourced data [10]. We additionally employed tuberculosis (TB) incidence (cases per 100,000 population) from the World Bank as a comparator outcome to assess model robustness (S6 Fig and S4 Table) [11]. While measles reflects acute immunization gaps, TB serves as a proxy for the long-term structural and socioeconomic deterioration, such as overcrowding and malnutrition, inherent in conflict-affected settings.

Our primary independent variable was the raw count of BRDs, sourced from the Uppsala Conflict Data Program (UCDP) Georeferenced Event Dataset [12]. These data include three types of conflict events: state-based conflict, nonstate conflict, and one-sided violence, and spans 1989–2023. We aggregated BRDs resulting from all three types of conflict events at the country-year level based on the location where deaths occurred. UCDP-derived BRDs were selected as our indicator of conflict intensity because they provide a standardized, continuous measure of severity; because of this, UCDP BRDs are often cited as one of the most reliable sources of conflict data [7]. Data on the proportion of the population that was forcibly displaced were drawn from the United Nations High Commissioner for Refugees (UNHCR), which compiles estimates based on inputs from the Expert Group on Refugee and Internally Displaced Persons Statistics and multiple data sources including censuses, surveys, and administrative records [13]. UNHCR defines forcibly displaced persons as refugees, asylum seekers, or internally displaced persons. For this analysis, exposure was defined as the annual country-level total of forcibly displaced persons by country of origin, aggregating internal displacement and external cross-border outflows due to incomplete disaggregated reporting across contexts. Population data were obtained from the World Bank to calculate the percentage of the population forcibly displaced [11].

To capture socioeconomic development, we constructed a latent variable using three observed indicators: gross domestic product (GDP) per capita, mean years of schooling, and life expectancy. GDP per capita data were obtained from the World Bank [11], and mean years of schooling and life expectancy were sourced from the United Nations Development Programme (UNDP) Human Development Index (HDI) [14]. The selection of these indicators was driven by their status as standardized, internationally comparable metrics of structural development. While these indicators provide a robust measure of socioeconomic conditions and stability, we recognize they are relatively slow-moving. They were prioritized here to ensure data completeness across the longitudinal study period and to minimize missingness that often affects higher-frequency indicators in conflict-affected settings.

We additionally included mean vaccination coverage rates, derived from WHO/UNICEF Joint Estimates of National Immunization Coverage (WUENIC) [15], in some model specifications as a proxy for overall health system performance [16]. The rationale for using a mean across 10 vaccines was to capture a composite measure of health system reach and infrastructure stability, which may be more representative of general primary care resilience than any single indicator. To improve comparability across country-years, we excluded vaccines with substantial imbalance in reporting, retaining data on 10 vaccines: Bacille Calmette-Guérin (BCG); first and third doses of diphtheria, tetanus, and pertussis–containing vaccine (DTP1, DTP3); birth dose of hepatitis B vaccine (HEPB-3); third dose of Haemophilus influenzae type B vaccine (HIB3); first and second doses of measles-containing vaccine (MCV1, MCV2); third dose of pneumococcal conjugate vaccine (PCV3); third dose of polio-containing vaccine (POL3); and first dose of rubella-containing vaccine (RCV1). We used this composite mean rather than measles-specific coverage (MCV1 only) to minimize the influence of reporting volatility. By averaging across multiple delivery platforms, including birth doses and multi-contact series, the index likely provides a more stable indicator of general primary care infrastructure and cold-chain stability in conflict-affected settings.

To ensure temporal alignment across disparate sources, all variables were aggregated using a standardized calendar-year window (January 1 to December 31). While reporting systems for conflict (UCDP), displacement (UNHCR), and immunization (WUENIC) operate under different institutional mandates, each provides validated annual estimates that correspond to the same temporal unit. We acknowledge that reporting lags exist, particularly in conflict zones where data may only be finalized 12–18 months post-event, but since our SEM framework utilizes lagged variables and finalized pre-2024 UCDP conflict data, these institutional reporting delays do not introduce synchronization bias into the model’s structural pathways.

### Statistical analysis

Highly skewed variables—including GDP per capita, BRDs, measles incidence, and tuberculosis incidence—were log-transformed prior to z-score standardization. For variables requiring log transformation, which included observations with the value zero, we added 1 prior to logging to retain those data points. All continuous variables were then standardized globally across the full dataset (centered at the grand mean with a SD of 1) rather than within-country. Standardization facilitates robust estimation and comparability of model coefficients, which can be interpreted as the expected standard deviation (SD) change in the outcome associated with a one SD change in the predictor. We note that even with standardization of our variables, all analyses rely on annual national-level aggregates, which may mask substantial subnational and temporal heterogeneity in conflict, displacement, and measles transmission dynamics. Accordingly, results should be interpreted as country-level associations and not as individual-level or subnational causal effects. To assess sensitivity to the treatment of zero-valued observations, we conducted robustness analyses using inverse hyperbolic sine (IHS) transformations for all zero-inflated variables, including measles cases, measles incidence per million, and BRDs. Unlike log(x + 1), the IHS transformation accommodates zero values without adding an arbitrary constant and approximates the log transformation for larger values. All IHS-transformed variables were standardized prior to model estimation, and structural specifications were otherwise identical to the main analyses.

We first conducted fixed-effects panel regression analyses using the plm package in R to validate key associations between our variables and inform SEM specification [17]. These preliminary models examined the relationships between: 1) armed conflict (measured as cumulative annual BRDs) and population displacement; 2) armed conflict, population displacement, and national economic development (measured as logged GDP per capita); and 3) all aforementioned variables and measles incidence, and were used to assess whether the observed data are comparable with hypothesized directional associations. As detailed in Table 2, this sequence was structured according to a path-building logic designed to systematically examine the individual predictors associated with measles burden within the broader conflict-measles framework. Initial specifications (Models 1–10) evaluated the association of contemporary and lagged conflict shocks with socioeconomic development and population displacement. Subsequent models (Models 11–14) examined the temporal autocorrelation of violence and the recursive associations between displacement and BRDs. The final block (Models 15–20) estimated the total association between conflict and measles burden while systematically adjusting for socioeconomic and vaccination mediators to observe the attenuation of the direct conflict coefficient and identify the primary statistical pathways. All models used country-year as the unit of analysis and a within estimator to account for unobserved, time-invariant heterogeneity across countries.

We then applied SEM as a theory- and regression-informed exploratory framework to jointly examine multiple hypothesized pathways linking armed conflict, population displacement, socioeconomic development, and measles outcomes within a single coherent system. SEM was selected for its ability to estimate multiple direct and indirect pathways simultaneously, incorporate latent constructs, and model mediating and covarying relationships; however, causal interpretation would require strong assumptions, including correct temporal ordering, the absence of unmeasured time-varying confounding, and minimal measurement error in key indicators. These assumptions are unlikely to be fully met in this setting, given potential misclassification and misreporting of BRDs and measles cases, as well as omitted time-varying factors such as vaccination campaigns, political instability, humanitarian access, and climate-related shocks. Accordingly, consistent with ongoing debate in the literature regarding the limitations of SEM for causal attributions [18,19], we interpret our results as exploratory associations that are informative for hypothesis generation [20].

To address potential model equivalence where alternative path structures might yield identical fit indices, we prioritized a theory-driven approach to identifying pathways. The structural specification was anchored by the temporal precedence of conflict (using lagged BRDs) and informed by our preliminary fixed-effects regressions, which validated the directional associations between conflict, displacement, and socioeconomic indicators prior to SEM estimation. This ensures the model represents a plausible chain of associations supported by both longitudinal data and humanitarian theory, rather than a purely data-driven configuration. All SEM analyses were conducted in R (version 4.3.2), using the lavaan [21], semTools [22], and tidyverse packages [23], and the SEM equations are captured in the S1 Text.

We specified a latent construct for socioeconomic development, determined by three indicators: GDP per capita, life expectancy, and mean years of schooling. As shown in Fig 1, socioeconomic development was regressed on the proportion of population displaced and standardized BRDs. As reported in the Results, all three indicators loaded strongly (>0.70) on the latent construct. In the baseline model, population displacement was modeled as an endogenous mediator predicted by BRDs, and standardized measles incidence was regressed on socioeconomic development, population displacement, and BRDs to capture both direct and indirect pathways linking conflict to measles incidence.

We estimated alternative SEM specifications that incorporated a 1-year lag of BRDs as an exogenous predictor of population displacement, socioeconomic development, and measles incidence, and contemporaneous BRDs were regressed on their lagged values to account for temporal autocorrelation. Lagged BRDs were used to test the hypothesis that prior-year conflict is a stronger predictor of contemporaneous measles incidence, reflecting the time required for conflict-related service disruptions and subsequent transmission dynamics to increase measles burden. Testing lagged BRDs also mitigates simultaneity bias by ensuring that conflict exposure temporally precedes observed changes in measles incidence. Additional models testing alternative lag structures for BRDs are described in the Supporting information.

We handled missing data using full information maximum likelihood (FIML), which provides unbiased estimates when missingness is conditional on observed covariates. Diagnostic regressions confirmed that while displacement missingness is associated with conflict intensity, this is accounted for by the inclusion of conflict as a primary predictor in our structural model. To ensure robustness, we cross-validated our results using auxiliary variable SEM, Multiple Imputation by Chained Equations (MICE), and subsample analyses restricted to countries with ≥80% data completeness (see S8 Table). Latent variables were scaled using standardized factor loadings, and estimation was permitted up to 500 iterations to ensure convergence. Prior to analysis, we assessed the pattern and extent of missingness across all variables included in the models, calculating the proportion of missing values per variable, overall, and stratified by year and country. As shown in the Supporting Information, we additionally visualized missingness patterns using heatmaps and UpSet plots to identify any co-occurring or systematic missing data (S1 and S2 Figs). These steps ensured that missing values were not concentrated in specific years, countries, or variables, reducing the risk of bias in the SEM estimates.

Model fit and parameter estimates were evaluated using standardized coefficients and established fit indices, including the Root Mean Square Error of Approximation (RMSEA), Comparative Fit Index (CFI), and Tucker–Lewis Index (TLI). Model fit was considered acceptable if RMSEA < 0.08 and CFI and TLI > 0.90. We additionally reported metrics to enable comparison of model fit, including standardized root mean square residual (SRMR), Akaike Information Criterion (AIC), and Bayesian Information Criterion (BIC). Results were visualized using path diagrams generated with the lavaanPlot package [24].

### Robustness

Robustness checks included substituting IHME GBD measles incidence for WHO measles data, alternatingly adding prior-year measles cases and incidence as a predictor of contemporaneous measles burden, replacing measles incidence with tuberculosis incidence, and adding mean vaccination coverage and MCV1 coverage as an additional mediator. All models retained the same latent construct for socioeconomic development and structural pathways for population displacement and BRDs, with contemporaneous and lagged effects included where appropriate. Model fit and standardized coefficients were assessed as in the primary analysis.

### Patients and public involvement

Patients and/or the public were not involved in the design, conduct, reporting, or dissemination of this research.

### Inclusivity in global research

Additional information regarding the ethical, cultural, and scientific considerations specific to inclusivity in global research is included in the Supporting Information (S2 Checklist).

### Use of AI tools

We used Claude 4.5 to assist with generating R code to visualize results. The tool was not used to generate analytic decisions, interpret results, or draft scientific content. All AI-assisted output was reviewed and verified by the authors against primary sources and analytic outputs; the authors take full responsibility for the final manuscript.

## Results

### Descriptive analysis

The final analytical dataset included 4,632 country-year observations globally from 2000 to 2023. Table 1 presents the summary statistics for key model variables, including both unstandardized and standardized values. BRDs were highly skewed, with a median of 0 and a mean of 267 deaths per country-year. Similarly, forcible population displacement figures were marked by right skew, with a median of 0%, a mean of 1%, and a SD of 5%. Unstandardized socioeconomic development indicators varied widely: the average GDP per capita was $13,130 (SD $21,664), life expectancy was 70.0 years (SD 8.8), and mean years of schooling was 8.3 years (SD 3.3). Measles cases ranged from near-zero to over a million cases in some settings (median 16; mean 2,250; SD 12,484), underscoring substantial heterogeneity across the study period.

**Table 1:** Descriptive statistics for conflict exposure, population displacement, socioeconomic development, and measles outcomes across 193 countries, 2000–2023.

Variable	Type	N	Mean	SD	Median	IQR
Battle-related deaths	Unstandardized	4,632	267	3,735	0.00	0.00
	Standardized	4,632	0.00	1.00	–0.31	0.00
Population displacement (% pop.)	Unstandardized	4,241	0.01	0.05	0.00	0.00
	Standardized	4,241	0.00	1.00	–0.26	0.01
GDP per capita (USD)	Unstandardized	4,566	13,130	21,664	4,345	13,389
	Standardized	4,566	0.00	1.00	–0.03	1.55
Health spending (% GDP)	Unstandardized	4,359	6.25	2.88	5.64	3.83
	Standardized	4,359	0.00	1.00	–0.21	1.33
Life expectancy (yrs)	Unstandardized	4,632	70.0	8.86	71.5	12.1
	Standardized	4,632	0.00	1.00	0.17	1.37
Vaccination coverage	Unstandardized	4,613	86.4	13.1	91.2	14.7
	Standardized	4,613	0.00	1.00	0.37	1.12
Mean years of schooling	Unstandardized	4,490	8.27	3.26	8.62	5.22
	Standardized	4,490	0.00	1.00	0.11	1.60
Measles cases (WHO)	Unstandardized	4,297	2,250	12,484	16	359
	Standardized	4,297	0.00	1.00	–0.16	1.84
TB incidence (WB)	Unstandardized	4,609	133	190	52	172
	Standardized	4,609	0.00	1.00	0.03	1.64

Note: Abbreviations: GDP = Gross Domestic Product; pop = population; TB = tuberculosis; WHO = World Health Organization; WB = World Bank; yrs = years.

### Regression analysis

Fixed-effects panel regressions supported the hypothesized associations between armed conflict, population displacement, economic development, and measles cases. As shown in Table 2, higher levels of BRDs were significantly associated with increased population displacement (β = 0.35; 95% CI [0.32, 0.39]; *p* < 0.001), with similar results when using a 1-year lag of BRDs. In contrast, greater population displacement was consistently associated with lower GDP per capita (β = −0.03; 95% CI [−0.05, −0.02]; *p* < 0.001 in Model 3), indicating economic losses accompany large-scale population movements. Both BRDs and population displacement remained negatively associated with GDP per capita across all model specifications, although the magnitude of the displacement association attenuated when vaccination coverage and other covariates were included. In contrast, vaccination coverage was positively associated with GDP per capita (β = 0.14; 95% CI [0.12, 0.16]; *p* < 0.001) and inversely associated with both population displacement (β = –0.16; 95% CI [–0.2, –0.12]; *p* < 0.001) and BRDs (β = −0.06; 95% CI [−0.09, −0.03]; *p* < 0.001), suggesting that conflict and forced displacement may be associated with weaker immunization systems. In models predicting measles incidence, higher vaccination coverage was strongly protective (β = −0.10; 95% CI [−0.14, −0.06]; *p* < 0.001), whereas neither BRDs nor population displacement showed a direct association with incidence in the models which included vaccination coverage. Collectively, these findings are consistent with the hypothesized structural associations between armed conflict and measles incidence, mediated through population displacement, economic disruption, and vaccine coverage.

### Structural equation modeling

All four models demonstrated excellent model fit across all specifications (CFI = 0.991–0.996; TLI = 0.976–0.989; RMSEA = 0.046–0.062; SRMR = 0.012–0.015), supporting the hypothesized pathways that link armed conflict, population displacement, socioeconomic development, and measles cases and incidence. Standardized coefficients were reported for all pathways.

#### Measurement model for socioeconomic development.

The latent construct for socioeconomic development was well-defined, with strong loadings for GDP per capita (β = 0.94; 95% CI [0.93, 0.95]; *p* < 0.001), life expectancy (ranging from β = 0.87; 95% CI [0.86, 0.88]; *p* < 0.001; to β = 0.88; 95% CI [0.87, 0.89]; *p* < 0.001), and mean years of schooling (ranging from β = 0.83; 95% CI [0.82, 0.84]; *p* < 0.001; to β = 0.84; 95% CI [0.83, 0.85]; *p* < 0.001). These results confirm that the three indicators captured a coherent dimension of national development.

#### Structural pathways.

As shown in Fig 2, both conflict and displacement were negatively associated with socioeconomic development. As shown in Table 3, each SD increase in displaced population percentage corresponded to a 0.20 SD decline in socioeconomic development (95% CI [−0.23, −0.17]; *p* < 0.001), while a one SD increase in standardized log-transformed BRDs was associated with a 0.10 SD decline (95% CI [−0.13, −0.07]; *p* < 0.001). When prior-year conflict was included, the contemporaneous association of current-year BRDs weakened (β = −0.04; 95% CI [−0.11, 0.03]; *p* = 0.247), whereas the prior-year association persisted (β = −0.07; 95% CI [−0.14, 0.01]; *p* = 0.072), suggesting delayed socioeconomic consequences of armed conflict.

Conflict was substantially associated with displacement. Current-year BRDs were positively associated with displacement (β = 0.37; 95% CI [0.32, 0.42]; *p* < 0.001), and the lagged association remained substantial and significant (β = 0.28; 95% CI [0.17, 0.39]; *p* < 0.001). Conflict intensity showed high temporal persistence (β = 0.90; 95% CI [0.88, 0.92]; *p* < 0.001), consistent with the protracted nature of armed violence.

#### Pathways to measles outcomes.

Higher socioeconomic development was consistently associated with lower measles cases and incidence. Each SD increase in the latent socioeconomic development construct corresponded to a 0.32 to 0.34 SD reduction in standardized measles cases and a 0.34 to 0.36 SD reduction in standardized measles incidence (all *p-values* < 0.001). Direct associations of conflict with measles were small. Higher contemporaneous BRDs (per one SD increase in log-transformed deaths) were weakly associated with higher measles cases (β = 0.17; 95% CI [0.14, 0.20]; *p* < 0.001), but this association attenuated after accounting for prior-year conflict (β = 0.05; 95% CI [−0.01, 0.11]; *p* = 0.091). Lagged BRDs, however, remained positively associated with measles cases (β = 0.14; 95% CI [0.08, 0.20]; *p* < 0.001), though this association was not significant for incidence after accounting for displacement (β = 0.04; 95% CI [–0.02, 0.11]; *p* = 0.164), consistent with delayed associations between conflict and health systems or vaccination coverage.

Population displacement showed no consistent direct association with measles outcomes after controlling for socioeconomic development (β ≈ 0; not significant in most models), indicating that for the case-count outcome its influence operates primarily through the indirect pathway of socioeconomic development. For measles incidence, however, displacement retained a small but consistent association across both incidence models: in Model C (without prior-year BRDs), a one SD increase in displaced population was associated with higher standardized measles incidence per million population (β = 0.06; 95% CI [0.03, 0.10]; *p* < 0.001); and this association persisted in the fully specified Model D incorporating prior-year BRDs (β = 0.06; 95% CI [0.03, 0.09]; *p* < 0.001). This suggests that displacement may have a modest direct association with population-level transmission rates beyond its indirect association through socioeconomic decline, potentially reflecting the concentration of susceptible individuals in under-served settings.

#### Model comparisons.

Including lagged conflict terms modestly improved model fit (e.g., RMSEA = 0.046 versus 0.052 for measles case models; CFI = 0.996 versus 0.993) and better captured temporal patterns in the associations. Differences in model performance indicators (AIC = 25,564–30,586; BIC = 25,686–30,746) were relatively small, and substantive conclusions were unchanged. Decomposition of conflict associations (S13 Table) reveals that the decomposition of associations differs by outcome metric. For measles incidence (Model C), indirect structural disruptions accounted for 73.9% of the total standardized association with conflict. This was primarily mediated by socioeconomic degradation (31.3%) and a sequential pathway where displacement-driven socioeconomic decline further exacerbated transmission (23.5%). Conversely, the influence on total case volume (Model A) was predominantly direct (72.9%). These findings suggest that structural conditions are most strongly associated with sustained population-level incidence, whereas active conflict is more strongly associated with absolute case surges.

#### Robustness analysis.

Robustness analyses supported the primary finding that armed conflict is associated with direct and indirect, via socioeconomic degradation and increased displacement, increases in measles incidence. The inclusion of vaccination coverage as an additional mediator showed that while higher socioeconomic development remained positively associated with coverage, conflict and displacement were negatively associated with both socioeconomic conditions and vaccination coverage (S3 Fig and S1 Table). Similarly, incorporating prior-year measles cases and incidence as mediators identified lagged measles incidence as a strong predictor of current cases without substantially altering the indirect pathways (S4 Fig and S2 Table). While core path coefficients remained stable in direction and significance, global model fit was variable (CFI 0.902–0.988, TLI 0.804–0.968, RMSEA 0.082–0.224). Specifications incorporating IHME incidence data and autoregressive lags (S5 Fig and S2 and S3 Tables) exhibited sub-optimal global fit (RMSEA > 0.10, TLI < 0.90), likely reflecting the complex covariance structures of these alternative datasets rather than a failure of the underlying structural logic.

Our primary models utilized the maximum likelihood estimation with robust standard errors (MLR) estimator with Huber-White sandwich standard errors to account for nonnormality and heteroscedasticity. Sensitivity analyses using cluster-robust standard errors (S9 Table) yielded wider CIs but stable point estimates; the association between the socioeconomic latent construct and measles incidence remained robust across all specifications (β = −0.34; 95% CI [−0.43, −0.25]; *p* < 0.001). Results using IHS-transformed variables were highly consistent with the primary log(x + 1) specifications (S5 Table). Latent construct loadings and the magnitude of associations between conflict, displacement, and measles outcomes remained substantively unchanged, suggesting that findings are robust to alternative transformations for zero-valued observations.

Models testing reverse pathways (measles incidence to displacement and conflict) performed poorly, whereas specifications permitting correlated residuals fit the data well (S6 Table). This pattern suggests observed associations reflect shared institutional and surveillance dynamics rather than direct feedback from outbreaks to conflict processes. A first-difference SEM focusing on year-over-year changes (S10 Table) confirmed the stability of the model’s internal logic: escalated displacement was significantly associated with a contemporaneous decline in the socioeconomic latent construct (β = −0.37; 95% CI [–0.59, –0.14]; *p* = 0.012). While annual changes in measles incidence were more volatile, the persistence of the displacement-socioeconomic link suggests the model captures genuine structural dynamics rather than secular trends (S6 and S12 Tables).

Extended lag analyses (S11 Table) indicated a sustained conflict association in Model AA (incidence counts), with a significant Wald joint significance test for Lags 0–3 (χ^2^ = 17.03, *p* = 0.002). Although individual Lags 1 and 2 were nonsignificant, likely due to multicollinearity, a 3rd-year lag (β = 0.09; 95% CI [–0.041, 0.236]; *p* = 0.168) may suggest a delayed peak in measles cases. Joint significance was not maintained in the rate-based Model BB (*p* = 0.298), indicating sensitivity to outcome specification. Further, the 3-year lag structure required data truncation that may introduce survival bias. Finally, accounting for first-order autoregressive [AR [1]] serial autocorrelation, either across all endogenous variables or for the outcome alone, did not materially alter the key structural pathways (S7 Table).

## Discussion

This global longitudinal analysis shows that armed conflict is modestly associated with higher measles burden directly and more strongly associated indirectly via lower socioeconomic development. Using structural equation modeling across 193 countries from 2000 to 2023, we found that contemporaneous conflict was directly associated with higher measles cases (β = 0.17; 95% CI [0.14, 0.20]; *p* < 0.001). While this association attenuated after accounting for prior-year conflict (β = 0.05; 95% CI [−0.01, 0.11]; *p* = 0.091), lagged BRDs remained positively associated with measles cases (β = 0.14; 95% CI [0.08, 0.20]; *p* < 0.001). In both model specifications, a one SD increase in standardized log-transformed armed conflict was associated with an overall increase of about 0.20 SD in measles cases, corresponding to roughly 2,500 additional reported cases per year per every ~3,700 BRDs. For context, the mean number of reported measles cases globally from 2000 to 2023 was ~2,250 per country-year, indicating that the estimated conflict-associated increase is substantial relative to the baseline annual burden.

We found that socioeconomic development was a strong protective factor against measles burden, and that armed conflict was adversely associated with development both directly and indirectly through population displacement. Each SD increase in development corresponded to a 0.32 to 0.34 SD reduction in standardized measles cases and a 0.34 to 0.36 SD reduction in standardized measles incidence (all *p-values* < 0.001), suggesting that stronger health, education, and economic systems collectively reduce population vulnerability to infectious disease risks. These findings suggest that efforts to maintain essential infrastructure and public services during conflict may help sustain population health.

Although displacement showed no consistent direct association with measles case counts after adjusting for socioeconomic development, it retained a small direct association with measles incidence rates and likely functions more broadly as an intermediate mechanism linking conflict to reduced health service provisioning and increased outbreak risk. This interpretation aligns with prior field studies documenting how mass displacement concentrates susceptible populations, disrupts service delivery, and facilitates measles transmission in humanitarian settings [5]. Lagged conflict was further associated with greater displacement and socioeconomic losses, suggesting that the conflict-associated differences in health-related indicators may persist well beyond active fighting. Such delayed associations underscore the need for sustained surveillance, early recovery investments, and the integration of displaced populations into routine immunization systems.

Our findings are broadly consistent with the limited literature quantifying the association between armed conflict and infectious disease outcomes. Previous analyses have shown that the odds of diphtheria occurrence in WHO African Region were 41% (odds ratio [OR] = 1.41; 95% CI [1.17, 1.68]) higher in countries experiencing conflict than in those not affected [25]; and that governorates in Yemen exposed to severe air raids had a 106% higher cholera incidence rate (95% CI [59%, 169%]) than those without recent air raids [26]. Evidence for other infectious diseases remains nonexistent or mixed: a 2024 systematic review identified no studies quantifying the association between armed conflict and measles incidence [5], while a 2023 review on tuberculosis outcomes found heterogeneous effects—six studies reported increases in case notifications following the onset of conflict, whereas three studies reported decreases [27]. Our findings are also consistent with a broader literature demonstrating that armed conflict frequently disrupts health and vaccination delivery systems, with ~85% of studies reporting declines in vaccination coverage in conflict-affected settings [7].

Several limitations warrant consideration. First, national-level aggregation introduces a risk of spatial dilution; by masking localized disruptions in conflict “hotspots,” national averages likely bias our coefficients toward the null. Furthermore, conflict-driven surveillance collapse and restricted healthcare access systematically induce under-reporting, suggesting that our findings represent a conservative lower bound of the true public health burden. While UCDP BRDs are a gold-standard metric, measurement error in remote or low-intensity settings may further contribute to attenuation bias, also leading to conservative estimates.

Second, the structural impacts of conflict follow complex temporal cycles that may be obscured by annual aggregation. Our reliance on annual panel data likely introduces temporal aggregation bias by smoothing over acute, short-term interactions such as monthly outbreak spikes or localized violence, which may attenuate our estimates toward the null. While this trade-off was necessary to maintain a global scope using standardized annual indicators, 1-year lags were utilized for parsimony, and exploratory analyses (S11 Table) identified a “temporal tail” peaking at the three-year mark. This delay likely reflects the biological accumulation of an “immunity gap”—a compounding pool of susceptible children that eventually reaches a critical threshold for explosive outbreaks. The durable nature of vaccination disruptions and the logistical hurdles of post-conflict catch-up campaigns suggest that primary estimates may not capture the full, multi-year cumulative burden. We also acknowledge the risk of spurious lagged effects; if the reporting of deaths is temporally displaced (e.g., due to delayed documentation in volatile regions), the observed timing of the socioeconomic and health impacts may be artificially shifted. These factors suggest that our findings should be interpreted with caution regarding the precise timing and total magnitude of conflict-associated differences.

Third, endogeneity and unmeasured confounding remain inherent risks. Armed conflict is rarely a purely exogenous force; it often emerges from localized political instability that may independently degrade infrastructure prior to active fighting. Furthermore, our models do not account for time-varying factors such as humanitarian access or aid intensity. A contraction of humanitarian space may be jointly associated with greater outbreak risk and reduced surveillance reliability, further obscuring the estimated association. Although we focus on the pathway from conflict to health, reciprocal relationships remain possible, as large-scale outbreaks can exacerbate socioeconomic instability and fuel social grievances. Consequently, our findings should be interpreted as structural associations that align with existing humanitarian theory, rather than as definitive causal evidence of directionality.

Finally, while FIML and Multiple Imputation yielded consistent structural associations, global fit indices in supplementary models (e.g., RMSEA) were sensitive to high-variance longitudinal data and lagged variables. This necessitates caution in interpreting absolute fit, though the stability of local path coefficients across all specifications strengthens our confidence in the core associations. Future research should leverage subnational and higher-frequency data—such as satellite-derived night-lights or service delivery measures—to clarify these mechanisms and evaluate threshold effects across other VPDs.

Despite these limitations, our findings have direct implications for global health policy and humanitarian response. Our analysis suggests that efforts to sustain vaccination programs in conflict-affected areas, extend outreach to displaced populations, and bolster socioeconomic resilience may yield the greatest returns in reducing measles outbreaks in conflict settings. Multilateral coordination between WHO, UNICEF, and UNHCR is essential to ensure that emergency response mechanisms address the structural determinants of disease vulnerability alongside immediate clinical needs.

## Supporting information

S1 Fig Missingness UpSet plot. Variables included are battle-related deaths (BRDs), population displacement (%), gross domestic product (GDP) per capita, life expectancy, mean years of schooling, measles cases, and mean vaccination coverage (%).
(DOCX)

S2 Fig Missingness over time by variable. Variables shown include battle-related deaths (BRDs), population displacement (%), gross domestic product (GDP) per capita, life expectancy, mean years of schooling, measles cases, and mean vaccination coverage.
(DOCX)

S1 Text Primary model specifications (Models A–D). (DOCX)

S3 Fig Structural equation models with mean vaccination coverage as an additional mediator (Models E–H). Path diagrams show standardized coefficients for structural equation models (SEMs) incorporating mean vaccination coverage as a mediator of the association between socioeconomic development, population displacement, armed conflict, and measles outcomes. Models E and F use standardized total measles cases as the outcome; Models G and H use measles incidence per million population. Models F and H additionally include 1-year lagged battle-related deaths (BRDs) to capture delayed conflict effects.
(DOCX)

S1 Table Structural equation model results with mean vaccination coverage as an additional mediator (Models E–H), 2000–2023. Models E and F use standardized total measles cases as the outcome; Models G and H use measles incidence per million population. Models F and H additionally include 1-year lagged battle-related deaths (BRDs) to capture delayed conflict effects. AIC = Akaike Information Criterion; BIC = Bayesian Information Criterion; BRDs = Battle-related deaths; CFI = Comparative Fit Index; TLI = Tucker–Lewis Index; RMSEA = Root Mean Square Error of Approximation; SRMR = Standardized Root Mean Square Residual.
(DOCX)

S4 Fig Structural equation models incorporating lagged measles outcomes as predictors (Models I–L). Models I and J use standardized total measles cases as the outcome (with lagged measles cases as a covariate); Models K and L use measles incidence per million population (with lagged incidence as a covariate). Models J and L additionally include 1-year lagged battle-related deaths (BRDs).
(DOCX)

S2 Table Structural equation model results incorporating lagged measles outcomes as predictors (Models I–L), 2000–2023. Models I and J use standardized total measles cases as the outcome (with lagged measles cases as a covariate); Models K and L use measles incidence per million population (with lagged incidence as a covariate). Models J and L additionally include 1-year lagged battle-related deaths (BRDs). AIC = Akaike Information Criterion; BIC = Bayesian Information Criterion; BRDs = Battle-related deaths; CFI = Comparative Fit Index; TLI = Tucker–Lewis Index; RMSEA = Root Mean Square Error of Approximation; SRMR = Standardized Root Mean Square Residual.
(DOCX)

S5 Fig Structural equation models using Institute for Health Metrics and Evaluation (IHME) Global Burden of Disease (GBD) measles incidence data (Models M–P). Path diagrams show standardized coefficients for structural equation models (SEMs) estimated using measles incidence data from the Institute for Health Metrics and Evaluation (IHME) Global Burden of Disease (GBD) study as an alternative to World Health Organization (WHO) measles data. Models M and N use IHME measles incidence counts without and with lagged battle-related deaths (BRDs), respectively. Models O and P additionally incorporate lagged measles incidence as a predictor.
(DOCX)

S3 Table Structural equation model results using Institute for Health Metrics and Evaluation (IHME) Global Burden of Disease (GBD) measles incidence data (Models M–P), 2000–2023. Models M and N use Institute for Health Metrics and Evaluation (IHME) Global Burden of Disease (GBD) measles incidence counts without and with lagged battle-related deaths (BRDs), respectively. Models O and P additionally incorporate lagged measles incidence as a predictor. AIC = Akaike Information Criterion; BIC = Bayesian Information Criterion; BRDs = Battle-related deaths; CFI = Comparative Fit Index; IHME = Institute for Health Metrics and Evaluation; TLI = Tucker–Lewis Index; RMSEA = Root Mean Square Error of Approximation; SRMR = Standardized Root Mean Square Residual.
(DOCX)

S6 Fig Structural equation models using tuberculosis incidence as a comparator outcome (Models Q–R). Path diagrams show standardized coefficients for structural equation models (SEMs) substituting tuberculosis (TB) incidence per 100,000 population (sourced from the World Bank) for measles incidence as the outcome variable. Model Q includes contemporaneous battle-related deaths (BRDs) only; Model R additionally incorporates 1-year lagged BRDs.
(DOCX)

S4 Table Structural equation model results using tuberculosis incidence as a comparator outcome (Models Q–R), 2000–2023. Structural equation models (SEMs) estimated standardized effects substituting tuberculosis (TB) incidence per 100,000 population (sourced from the World Bank) for measles incidence as the outcome variable. Model Q includes contemporaneous battle-related deaths (BRDs) only; Model R additionally incorporates 1-year lagged BRDs. AIC = Akaike Information Criterion; BIC = Bayesian Information Criterion; BRDs = Battle-related deaths; CFI = Comparative Fit Index; TB = Tuberculosis; TLI = Tucker–Lewis Index; RMSEA = Root Mean Square Error of Approximation; SRMR = Standardized Root Mean Square Residual.
(DOCX)

S5 Table Structural equation model results using Inverse Hyperbolic Sine (IHS) transformations in place of log(x + 1) transformations (Models S–V), 2000–2023. Models S and T use standardized total measles cases as the outcome; Models U and V use measles incidence per million population. Models U and V additionally include 1-year lagged battle-related deaths (BRDs). AIC = Akaike Information Criterion; BIC = Bayesian Information Criterion; BRDs = Battle-related deaths; CFI = Comparative Fit Index; TLI = Tucker–Lewis Index; RMSEA = Root Mean Square Error of Approximation; SRMR = Standardized Root Mean Square Residual.
(DOCX)

S6 Table Structural equation model results testing bidirectional and correlated-error specifications, 2000–2023. AIC = Akaike Information Criterion; BIC = Bayesian Information Criterion; BRDs = Battle-related deaths; CFI = Comparative Fit Index; TLI = Tucker–Lewis Index; RMSEA = Root Mean Square Error of Approximation; SRMR = Standardized Root Mean Square Residual.
(DOCX)

S7 Table Structural equation model results with explicit modeling of first-order autoregressive [AR(1)] serial dependence, 2000–2023. The full first-order autoregressive [AR(1)] model accounts for first-order serial dependence by including autoregressive paths for both exposure and outcome variables, whereas the outcome-only model restricts these paths to the dependent variable alone. AIC = Akaike Information Criterion; BIC = Bayesian Information Criterion; BRDs = Battle-related deaths; CFI = Comparative Fit Index; TLI = Tucker–Lewis Index; RMSEA = Root Mean Square Error of Approximation; SRMR = Standardized Root Mean Square Residual.
(DOCX)

S8 Table Structural path coefficients under alternative missing data specifications (Model A), 2000–2023. FIML = Full Information Maximum Likelihood.
(DOCX)

S9 Table Structural equation model results with cluster-robust standard errors adjusted for within-country correlation (Models W-Z), 2000–2023. Models W and X use standardized total measles cases as the outcome; Models Y and Z use measles incidence per million population. Models X and Z additionally include 1-year lagged battle-related deaths (BRDs). AIC = Akaike Information Criterion; BIC = Bayesian Information Criterion; BRDs = Battle-related deaths; CFI = Comparative Fit Index; TLI = Tucker–Lewis Index; RMSEA = Root Mean Square Error of Approximation; SE = Standard Error; SRMR = Standardized Root Mean Square Residual.
(DOCX)

S10 Table First-difference structural equation model results examining year-over-year changes in armed conflict, population displacement, socioeconomic development, and measles incidence, 2001–2023. CFI = Comparative Fit Index; RMSEA = Root Mean Square Error of Approximation; SRMR = Standardized Root Mean Square Residual.
(DOCX)

S11 Table Extended lag analysis: distributed-lag structural equation model results examining conflict effects on measles outcomes over a 3-year temporal horizon (Models AA and BB), 2003–2023. AIC = Akaike Information Criterion; BIC = Bayesian Information Criterion; BRDs = Battle-related deaths; CFI = Comparative Fit Index; TLI = Tucker–Lewis Index; RMSEA = Root Mean Square Error of Approximation; SE = Standard Error; SRMR = Standardized Root Mean Square Residual.
(DOCX)

S12 Table Parallel mediation analysis disaggregating socioeconomic development into economic level (gross domestic product [GDP] per capita) and human capital (life expectancy and mean years of schooling), 2000–2023. CI = confidence interval; GDP = gross domestic product.
(DOCX)

S13 Table Hierarchical decomposition of the total effect of armed conflict on measles burden into direct and indirect pathways (Models A–D), 2000–2023. Models A and B use standardized total measles cases as the outcome; Models C and D use measles incidence per million population. Models B and D additionally include 1-year lagged battle-related deaths (BRDs). BRD = Battle-related deaths; Disp = displacement; SE = socioeconomic development; Socioecon = socioeconomic development.
(DOCX)

S1 Checklist STROBE checklist—checklist of items that should be included in reports of cross-sectional studies. Reproduced under the Creative Commons Attribution 4.0 International License (CC BY 4.0). Available from: https://www.strobe-statement.org/.
(DOCX)

S2 Checklist Inclusivity in global research checklist. (DOCX)

## Abbreviations

AIC: Akaike Information Criterion
BCG: Bacille Calmette-Guérin
BIC: Bayesian Information Criterion
BRDs: battle-related deaths
CFI: Comparative Fit Index
CI: Confidence Interval
DV: dependent variables
FIML: full information maximum likelihood
GDP: gross domestic product
GBD: Global Health Burden of Disease
HDI: Human Development Index
IHS: inverse hyperbolic sine
IHME: Institute for Health Metrics and Evaluation
MICE: Multiple Imputation by Chained Equations
OR: odds ratio
RMSEA: Root Mean Square Error of Approximation
SD: standard deviation
SEMs: structural equation models
TLI: Tucker–Lewis Index
UCDP: Uppsala Conflict Data Program
UNDP: United Nations Development Programme
UNHCR: United Nations High Commissioner for Refugees
VPD: vaccine-preventable disease
WHO: World Health Organization.
