# Supplementary material for: Association of armed conflict and global measles cases: A structural equation modeling analysis of 193 countries from 2000 to 2023
Source: PLoS Med. 2026 Jun 25;23(6):e1004819. doi: 10.1371/journal.pmed.1004819 (PMC13298743; doi:10.1371/journal.pmed.1004819)
Supplement: S1 Fig — Variables included are battle-related deaths (BRDs), population displacement (%), gross domestic product (GDP) per capita, life expectancy, mean years of schooling, measles cases, and mean vaccination coverage (%). (DOCX) [file pmed.1004819.s001.docx]

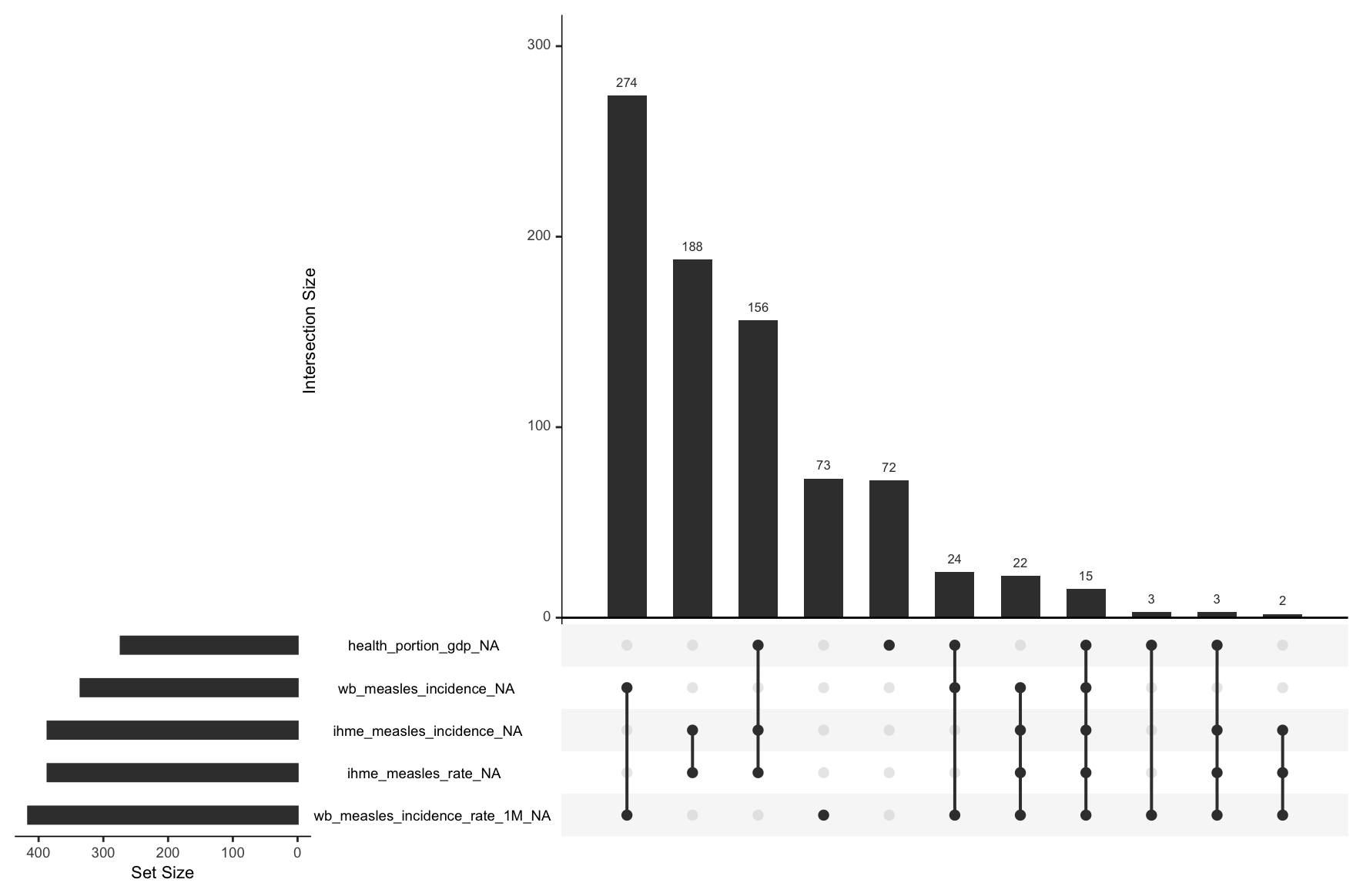
 S1 Fig. Missingness UpSet plot.

**Note:** The UpSet plot displays co-occurring patterns of missing data across key model variables. Variables included are battle-related deaths (BRDs), population displacement (%), gross domestic product (GDP) per capita, life expectancy, mean years of schooling, measles cases, and mean vaccination coverage (%).
