# Supplementary material for: Association of armed conflict and global measles cases: A structural equation modeling analysis of 193 countries from 2000 to 2023
Source: PLoS Med. 2026 Jun 25;23(6):e1004819. doi: 10.1371/journal.pmed.1004819 (PMC13298743; doi:10.1371/journal.pmed.1004819)
Supplement: S2 Fig — Variables shown include battle-related deaths (BRDs), population displacement (%), gross domestic product (GDP) per capita, life expectancy, mean years of schooling, measles cases, and mean vaccination coverage. (DOCX) [file pmed.1004819.s002.docx]

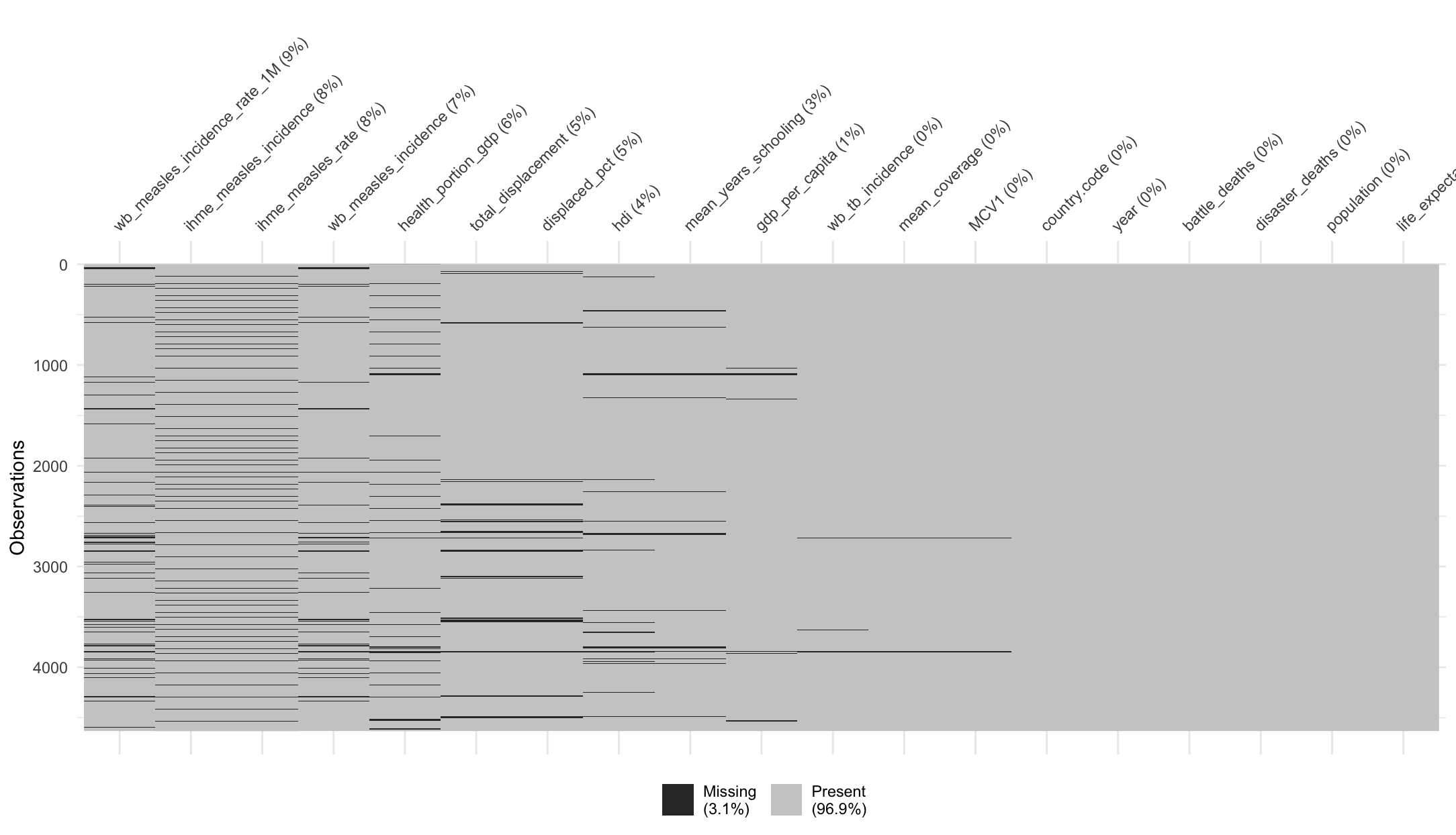


S2 Fig. Missingness over time by variable.

**Note:** The longitudinal plot displays the proportion of country-level observations with missing data for each key model variable across the study period (2000-2023). Variables shown include battle-related deaths (BRDs), population displacement (%), gross domestic product (GDP) per capita, life expectancy, mean years of schooling, measles cases, and mean vaccination coverage.
