# Supplementary material for: Association of armed conflict and global measles cases: A structural equation modeling analysis of 193 countries from 2000 to 2023
Source: PLoS Med. 2026 Jun 25;23(6):e1004819. doi: 10.1371/journal.pmed.1004819 (PMC13298743; doi:10.1371/journal.pmed.1004819)
Supplement: S1 Text — (DOCX) [file pmed.1004819.s003.docx]

S1 Text. Primary model specifications (Models A–D).

**Measurement Model (Defining the Latent Socioeconomic Variable** $\boldsymbol{\eta}_{\boldsymbol{SE}}$**)**

$$GDP_{z}=\lambda_{1}\eta_{SE}+\epsilon_{1}$$

$$LifeExp_{z}=\lambda_{2}\eta_{SE}+\epsilon_{2}$$

$$Schooling_{z}=\lambda_{3}\eta_{SE}+\epsilon_{3}$$

**Structural Equations for Models A and C**

*Note that Model A uses measles cases (*$\boldsymbol{Y}_{\boldsymbol{cases}}$*); Model C uses Measles Rate (*$\boldsymbol{Y}_{\boldsymbol{rate}}$*). The structure is otherwise identical.*

$$\left( 1 \right) Displaced_{z}=\gamma_{1}BattleDeaths_{z}+\zeta_{1}$$

$$\left( 2 \right) \eta_{SE}=\gamma_{2}Displaced_{z}+\gamma_{3}BattleDeaths_{z}+\zeta_{2}$$

$$\left( 3 \right) Y_{measles}=\beta_{1}\eta_{SE}+\gamma_{4}BattleDeaths_{z}+\gamma_{5}Displaced_{z}+\zeta_{3}$$

**Structural Equations for Models B and D (Lagged)**

*These models include the autoregressive path for conflict and lagged predictors. Model B uses measles cases (*$\boldsymbol{Y}_{\boldsymbol{cases}}$*); Model D uses Measles Rate (*$\boldsymbol{Y}_{\boldsymbol{rate}}$*).*

$$\left( 1 \right)BattleDeaths_{t}=\rho_{1}BattleDeaths_{t-1}+\zeta_{0}$$

$$\left( 2 \right) Displaced_{z}=\gamma_{1}BattleDeaths_{t}+\gamma_{2}BattleDeaths_{t-1}+\zeta_{1}$$

$$\left( 3 \right) \eta_{SE}=\gamma_{3}Displaced_{z}+\gamma_{4}BattleDeaths_{t}+\gamma_{5}BattleDeaths_{t-1}+\zeta_{2}$$

$$\left( 4 \right) Y_{measles}=\beta_{1}\eta_{SE}+\gamma_{6}BattleDeaths_{t}+\gamma_{7}Displaced_{z}+\gamma_{8}BattleDeaths_{t-1}+\zeta_{3}$$

**Variable Definition Key**

Where:

$\boldsymbol{\eta}_{\boldsymbol{SE}}$​: Latent Socioeconomic Status (standardized)

$\boldsymbol{Y}_{\boldsymbol{cases}}$**:** Outcome variable (standardized cases for models A/B; incidence rate for models C/D)

λ: Factor loadings

γ: Regression coefficients for exogenous predictors

β: Regression coefficient for the latent variable

ρ: Autoregressive coefficient

ϵ: Measurement error terms

ζ: Structural disturbance (residual) terms

t and t−1: Represent current year and previous year, respectively.
