# Supplementary material for: Association of armed conflict and global measles cases: A structural equation modeling analysis of 193 countries from 2000 to 2023
Source: PLoS Med. 2026 Jun 25;23(6):e1004819. doi: 10.1371/journal.pmed.1004819 (PMC13298743; doi:10.1371/journal.pmed.1004819)
Supplement: S3 Fig — Path diagrams show standardized coefficients for structural equation models (SEMs) incorporating mean vaccination coverage as a mediator of the association between socioeconomic development, population displacement, armed conflict, and measles outcomes. Models E and F use standardized total measles cases as the outcome; Models G and H use measles incidence per million population. Models F and H additionally include 1-year lagged battle-related deaths (BRDs) to capture delayed conflict effects. (DOCX) [file pmed.1004819.s004.docx]

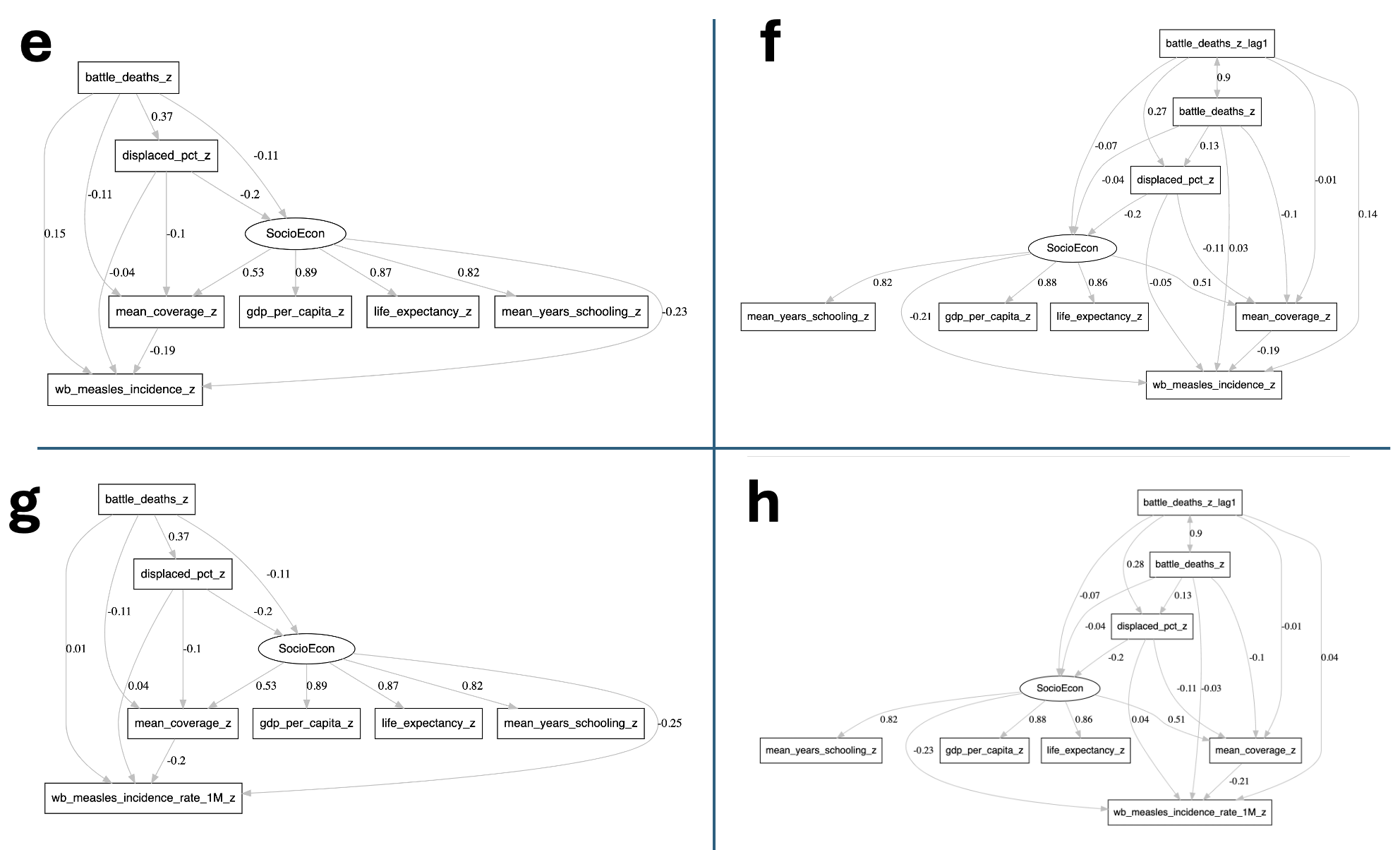


S3 Fig. Structural equation models with mean vaccination coverage as an additional mediator (Models E–H).

**Note*:*** Path diagrams show standardized coefficients for structural equation models (SEMs) incorporating mean vaccination coverage as a mediator of the association between socioeconomic development, population displacement, armed conflict, and measles outcomes. Models E and F use standardized total measles cases as the outcome; Models G and H use measles incidence per million population. Models F and H additionally include one-year lagged battle-related deaths (BRDs) to capture delayed conflict effects. Socioeconomic development is modeled as a latent construct defined by gross domestic product (GDP) per capita, life expectancy, and mean years of schooling.
