# Supplementary material for: Association of armed conflict and global measles cases: A structural equation modeling analysis of 193 countries from 2000 to 2023
Source: PLoS Med. 2026 Jun 25;23(6):e1004819. doi: 10.1371/journal.pmed.1004819 (PMC13298743; doi:10.1371/journal.pmed.1004819)
Supplement: S1 Table — Models E and F use standardized total measles cases as the outcome; Models G and H use measles incidence per million population. Models F and H additionally include 1-year lagged battle-related deaths (BRDs) to capture delayed conflict effects. AIC = Akaike Information Criterion; BIC = Bayesian Information Criterion; BRDs = Battle-related deaths; CFI = Comparative Fit Index; TLI = Tucker–Lewis Index; RMSEA = Root Mean Square Error of Approximation; SRMR = Standardized Root Mean Square Residual. (DOCX) [file pmed.1004819.s005.docx]

S1 Table. Structural equation model results with mean vaccination coverage as an additional mediator (Models E–H), 2000–2023.

| Effect | Model E | Model F | Model G | Model H |
| --- | --- | --- | --- | --- |
| GDP per capita → Socioeconomic development | 0.92 [0.91, 0.93]*** | 0.92 [0.91, 0.93]*** | 0.92 [0.91, 0.92]*** | 0.92 [0.91, 0.93]*** |
| Life expectancy → Socioeconomic development | 0.89 [0.88, 0.90]*** | 0.89 [0.88, 0.90]*** | 0.90 [0.89, 0.91]*** | 0.89 [0.88, 0.90]*** |
| Mean years of schooling → Socioeconomic development | 0.84 [0.83, 0.85]*** | 0.84 [0.83, 0.85]*** | 0.84 [0.83, 0.85]*** | 0.84 [0.83, 0.85]*** |
| Population displacement (%) → Socioeconomic development | -0.19 [-0.23, -0.16]*** | -0.19 [-0.23, -0.16]*** | -0.19 [-0.23, -0.16]*** | -0.19 [-0.23, -0.16]*** |
| BRDs → Socioeconomic development | -0.10 [-0.13, -0.07]*** | -0.04 [-0.11, 0.03] | -0.10 [-0.13, -0.07]*** | -0.04 [-0.11, 0.03] |
| Socioeconomic development → Mean vaccination coverage | 0.54 [0.52, 0.57]*** | 0.53 [0.51, 0.56]*** | 0.54 [0.52, 0.57]*** | 0.53 [0.51, 0.56]*** |
| Population displacement (%) → Mean vaccination coverage | -0.10 [-0.13, -0.07]*** | -0.11 [-0.14, -0.08]*** | -0.10 [-0.13, -0.07]*** | -0.11 [-0.14, -0.08]*** |
| BRDs → Mean vaccination coverage | -0.11 [-0.13, -0.08]*** | -0.10 [-0.17, -0.03]** | -0.11 [-0.13, -0.08]*** | -0.10 [-0.17, -0.03]** |
| BRDs → Population displacement (%) | 0.37 [0.32, 0.42]*** | 0.13 [0.03, 0.24]* | 0.37 [0.32, 0.42]*** | 0.13 [0.03, 0.24]* |
| Socioeconomic development → Measles cases | -0.24 [-0.27, -0.20]*** | -0.22 [-0.25, -0.19]*** | NA | NA |
| BRDs → Measles cases | 0.15 [0.12, 0.18]*** | 0.03 [-0.03, 0.09] | NA | NA |
| Population displacement (%) → Measles cases | -0.04 [-0.06, -0.01]** | -0.05 [-0.07, -0.02]*** | NA | NA |
| Mean vaccination coverage → Measles cases | -0.19 [-0.23, -0.15]*** | -0.19 [-0.23, -0.16]*** | NA | NA |
| BRDs (1-year lag) → Socioeconomic development | NA | -0.07 [-0.14, 0.01]. | NA | -0.07 [-0.14, 0.01]. |
| BRDs (1-year lag) → Mean vaccination coverage | NA | -0.01 [-0.07, 0.06] | NA | -0.01 [-0.07, 0.06] |
| BRDs (1-year lag) → Population displacement (%) | NA | 0.28 [0.17, 0.39]*** | NA | 0.28 [0.17, 0.39]*** |
| BRDs (1-year lag) → Measles cases | NA | 0.14 [0.08, 0.20]*** | NA | NA |
| Socioeconomic development → Measles incidence per million | NA | NA | -0.25 [-0.29, -0.22]*** | -0.24 [-0.27, -0.20]*** |
| BRDs → Measles incidence per million | NA | NA | 0.01 [-0.01, 0.04] | -0.03 [-0.09, 0.04] |
| Population displacement (%) → Measles incidence per million | NA | NA | 0.04 [0.01, 0.07]* | 0.04 [0.00, 0.07]* |
| Mean vaccination coverage → Measles incidence per million | NA | NA | -0.20 [-0.24, -0.16]*** | -0.21 [-0.25, -0.17]*** |
| BRDs (1-year lag) → Measles incidence per million | NA | NA | NA | 0.05 [-0.02, 0.11] |
| CFI | 0.96 | 0.96 | 0.96 | 0.96 |
| TLI | 0.90 | 0.90 | 0.90 | 0.90 |
| RMSEA | 0.12 | 0.11 | 0.12 | 0.10 |
| SRMR | 0.02 | 0.02 | 0.02 | 0.02 |
| AIC | 36,613.32 | 34,611.59 | 36,463.70 | 34454.23 |
| BIC | 36,774.34 | 34,797.14 | 36,624.72 | 34639.78 |

**Note:** Structural equation models (SEMs) estimated standardized effects. Models E and F use standardized total measles cases as the outcome; Models G and H use measles incidence per million population. Models F and H additionally include one-year lagged battle-related deaths (BRDs) to capture delayed conflict effects. Within each model, the regression of each endogenous variable is adjusted for all of its directly antecedent variables in the path diagram. Specifically: (i) socioeconomic development (latent, indicated by GDP per capita, life expectancy, and mean years of schooling) is adjusted for contemporaneous BRDs and population displacement (and one-year-lagged BRDs in Models F and H); (ii) population displacement is adjusted for contemporaneous BRDs (and lagged BRDs in Models F and H); (iii) the measles outcome (cases in Models E and F; incidence per million in Models G and H) is adjusted for socioeconomic development, population displacement, and contemporaneous BRDs (and lagged BRDs in Models F and H). Values represent standardized path coefficients with 95% confidence intervals in brackets and two-sided p-values from Wald tests rounded to three decimal places. AIC = Akaike Information Criterion; BIC = Bayesian Information Criterion; BRDs = battle-related deaths; CFI = Comparative Fit Index; TLI = Tucker-Lewis Index; RMSEA = Root Mean Square Error of Approximation; SRMR = Standardized Root Mean Square Residual.
