# Supplementary material for: Association of armed conflict and global measles cases: A structural equation modeling analysis of 193 countries from 2000 to 2023
Source: PLoS Med. 2026 Jun 25;23(6):e1004819. doi: 10.1371/journal.pmed.1004819 (PMC13298743; doi:10.1371/journal.pmed.1004819)
Supplement: S4 Fig — Models I and J use standardized total measles cases as the outcome (with lagged measles cases as a covariate); Models K and L use measles incidence per million population (with lagged incidence as a covariate). Models J and L additionally include 1-year lagged battle-related deaths (BRDs). (DOCX) [file pmed.1004819.s006.docx]

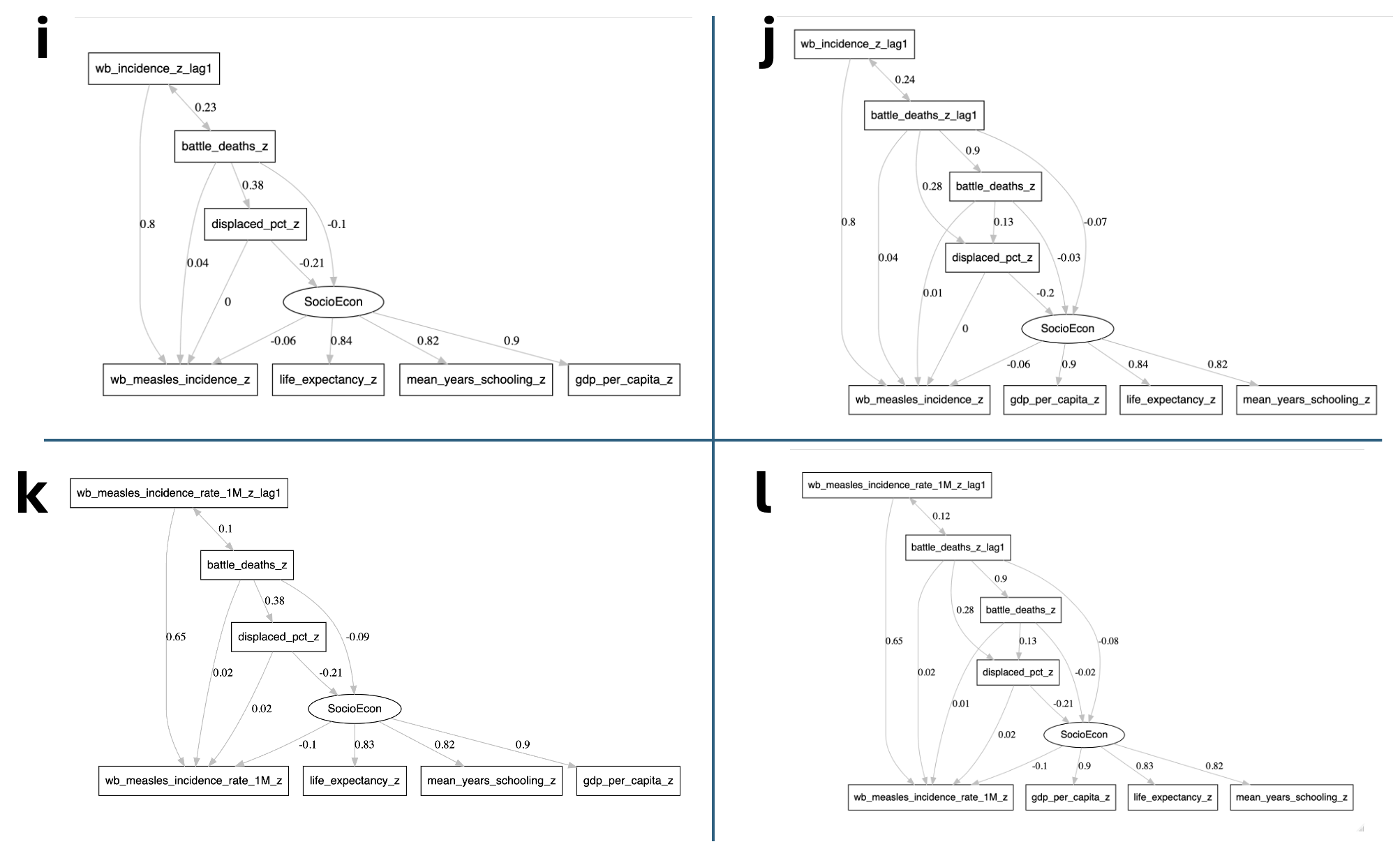


S4 Fig. Structural equation models incorporating lagged measles outcomes as predictors (Models I–L).

**Note:** Path diagrams show standardized coefficients for structural equation models (SEMs) that include prior-year measles burden as an additional predictor to adjust for temporal autocorrelation in disease outcomes. Models I and J use standardized total measles cases as the outcome (with lagged measles cases as a covariate); Models K and L use measles incidence per million population (with lagged incidence as a covariate). Models J and L additionally include one-year lagged battle-related deaths (BRDs). Socioeconomic development is modeled as a latent construct defined by gross domestic product (GDP) per capita, life expectancy, and mean years of schooling.
