# Supplementary material for: Association of armed conflict and global measles cases: A structural equation modeling analysis of 193 countries from 2000 to 2023
Source: PLoS Med. 2026 Jun 25;23(6):e1004819. doi: 10.1371/journal.pmed.1004819 (PMC13298743; doi:10.1371/journal.pmed.1004819)
Supplement: S5 Fig — Path diagrams show standardized coefficients for structural equation models (SEMs) estimated using measles incidence data from the Institute for Health Metrics and Evaluation (IHME) Global Burden of Disease (GBD) study as an alternative to World Health Organization (WHO) measles data. Models M and N use IHME measles incidence counts without and with lagged battle-related deaths (BRDs), respectively. Models O and P additionally incorporate lagged measles incidence as a predictor. (DOCX) [file pmed.1004819.s008.docx]

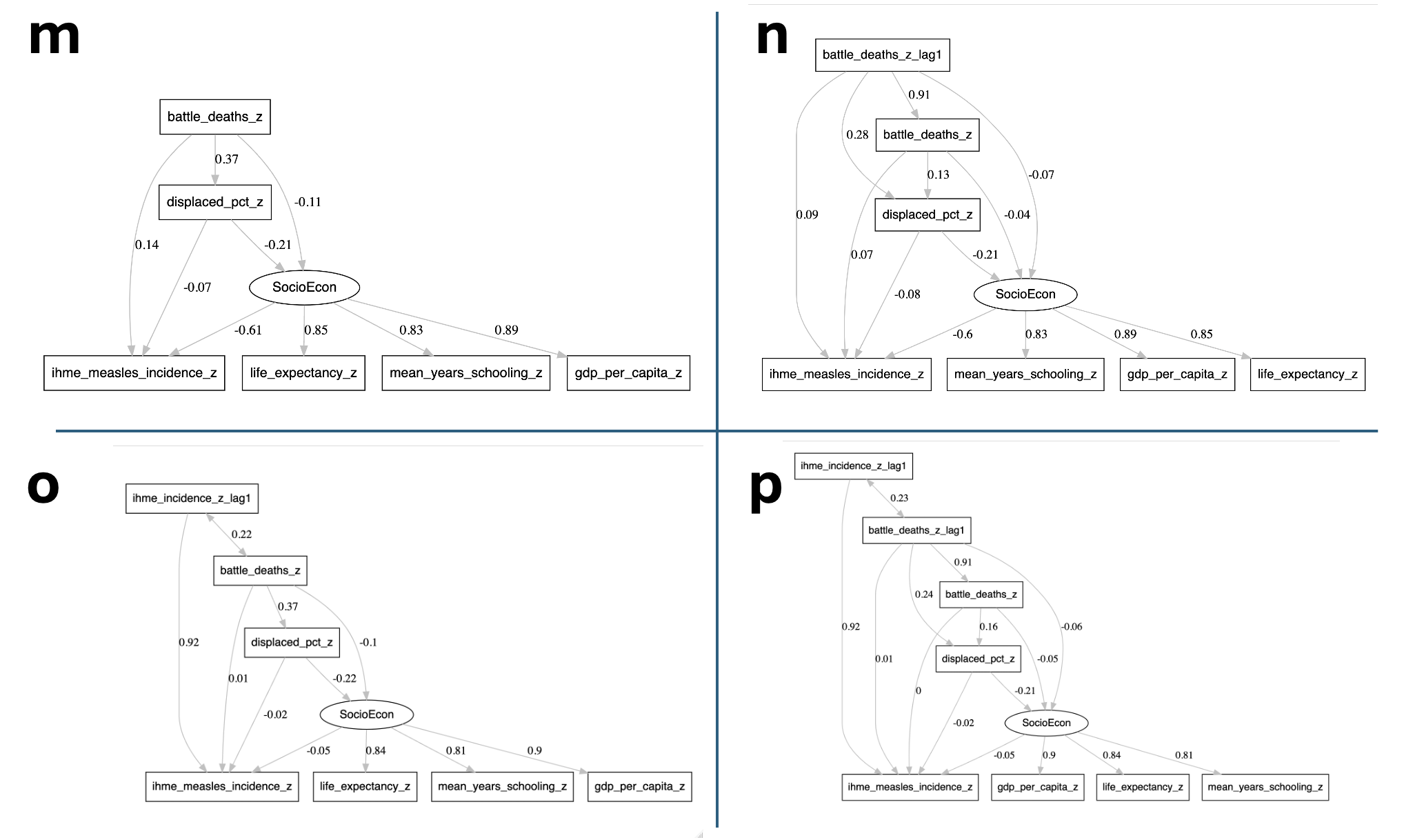


S5 Fig. Structural equation models using Institute for Health Metrics and Evaluation (IHME) Global Burden of Disease (GBD) measles incidence data (Models M–P).

**Note**: Path diagrams show standardized coefficients for structural equation models (SEMs) estimated using measles incidence data from the Institute for Health Metrics and Evaluation (IHME) Global Burden of Disease (GBD) study as an alternative to World Health Organization (WHO) measles data. Models M and N use IHME measles incidence counts without and with lagged battle-related deaths (BRDs), respectively. Models O and P additionally incorporate lagged measles incidence as a predictor. Socioeconomic development is modeled as a latent construct defined by gross domestic product (GDP) per capita, life expectancy, and mean years of schooling.
