# Supplementary material for: Association of armed conflict and global measles cases: A structural equation modeling analysis of 193 countries from 2000 to 2023
Source: PLoS Med. 2026 Jun 25;23(6):e1004819. doi: 10.1371/journal.pmed.1004819 (PMC13298743; doi:10.1371/journal.pmed.1004819)
Supplement: S6 Fig — Path diagrams show standardized coefficients for structural equation models (SEMs) substituting tuberculosis (TB) incidence per 100,000 population (sourced from the World Bank) for measles incidence as the outcome variable. Model Q includes contemporaneous battle-related deaths (BRDs) only; Model R additionally incorporates 1-year lagged BRDs. (DOCX) [file pmed.1004819.s010.docx]

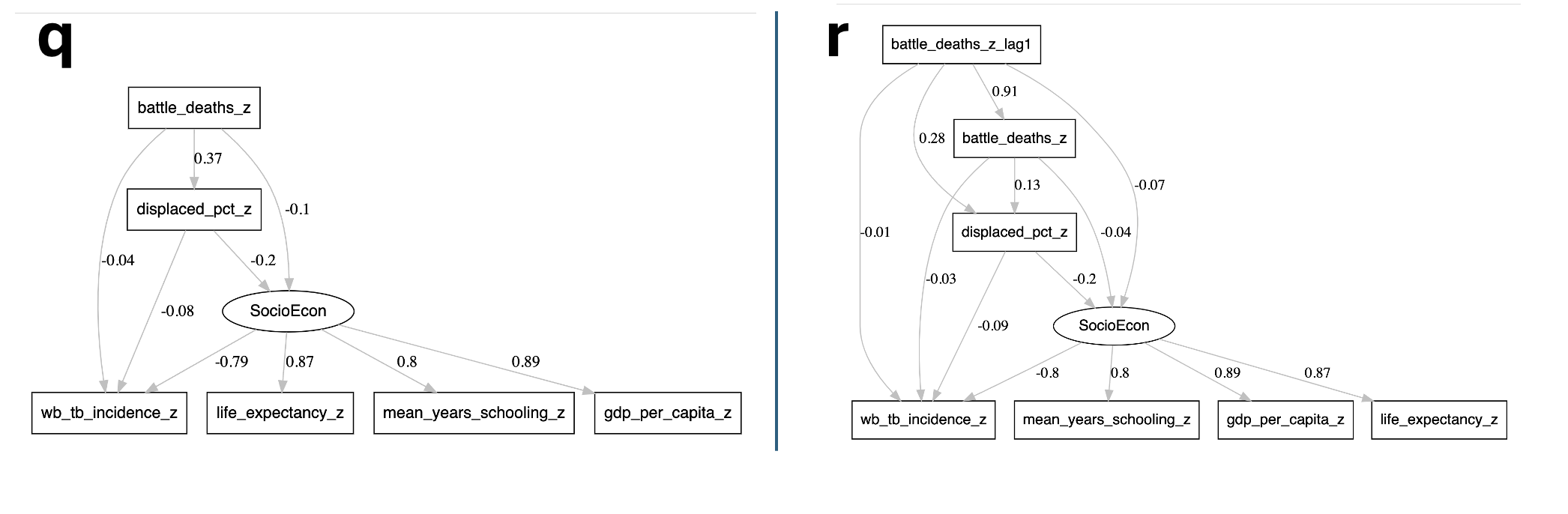


S6 Fig. Structural equation models using tuberculosis incidence as a comparator outcome (Models Q–R).

**Note:** Path diagrams show standardized coefficients for structural equation models (SEMs) substituting tuberculosis (TB) incidence per 100,000 population (sourced from the World Bank) for measles incidence as the outcome variable. Model Q includes contemporaneous battle-related deaths (BRDs) only; Model R additionally incorporates one-year lagged BRDs. Socioeconomic development is modeled as a latent construct defined by gross domestic product (GDP) per capita, life expectancy, and mean years of schooling. These models assess whether the indirect pathways linking conflict and displacement to infectious disease burden extend beyond measles to a disease with different transmission dynamics.
