# Supplementary material for: Association of armed conflict and global measles cases: A structural equation modeling analysis of 193 countries from 2000 to 2023
Source: PLoS Med. 2026 Jun 25;23(6):e1004819. doi: 10.1371/journal.pmed.1004819 (PMC13298743; doi:10.1371/journal.pmed.1004819)
Supplement: S4 Table — Structural equation models (SEMs) estimated standardized effects substituting tuberculosis (TB) incidence per 100,000 population (sourced from the World Bank) for measles incidence as the outcome variable. Model Q includes contemporaneous battle-related deaths (BRDs) only; Model R additionally incorporates 1-year lagged BRDs. AIC = Akaike Information Criterion; BIC = Bayesian Information Criterion; BRDs = Battle-related deaths; CFI = Comparative Fit Index; TB = Tuberculosis; TLI = Tucker–Lewis Index; RMSEA = Root Mean Square Error of Approximation; SRMR = Standardized Root Mean Square Residual. (DOCX) [file pmed.1004819.s011.docx]

S4 Table. Structural equation model results using tuberculosis incidence as a comparator outcome (Models Q–R), 2000–2023.

| Effect | Model Q | Model R |
| --- | --- | --- |
| GDP per capita → Socioeconomic development | 0.92 [0.91, 0.93]*** | 0.92 [0.91, 0.93]*** |
| Life expectancy → Socioeconomic development | 0.90 [0.89, 0.91]*** | 0.90 [0.89, 0.91]*** |
| Mean years of schooling → Socioeconomic development | 0.82 [0.81, 0.83]*** | 0.82 [0.81, 0.83]*** |
| Population displacement (%) → Socioeconomic development | -0.20 [-0.23, -0.16]*** | -0.19 [-0.23, -0.16]*** |
| BRDs → Socioeconomic development | -0.10 [-0.13, -0.07]*** | -0.04 [-0.11, 0.03] |
| BRDs → Population displacement (%) | 0.37 [0.32, 0.42]*** | 0.13 [0.03, 0.24]* |
| Socioeconomic development → TB incidence | -0.82 [-0.83, -0.81]*** | -0.82 [-0.83, -0.81]*** |
| BRDs → TB incidence | -0.04 [-0.05, -0.02]*** | -0.03 [-0.07, 0.01] |
| Population displacement (%) → TB incidence | -0.08 [-0.10, -0.06]*** | -0.08 [-0.10, -0.07]*** |
| BRDs (1-year lag) → Socioeconomic development | NA | -0.06 [-0.13, 0.01]. |
| BRDs (1-year lag) → Population displacement (%) | NA | 0.28 [0.17, 0.39]*** |
| BRDs (1-year lag) → BRDs | NA | 0.90 [0.88, 0.92]*** |
| BRDs (1-year lag) → TB incidence | NA | -0.01 [-0.05, 0.03] |
| CFI | 0.98 | 0.983 |
| TLI | 0.94 | 0.955 |
| RMSEA | 0.12 | 0.103 |
| SRMR | 0.02 | 0.02 |
| AIC | 23,175.94 | 27,019.55 |
| BIC | 23,298.31 | 27,179.50 |

**Note**: Structural equation models (SEMs) estimated standardized effects substituting tuberculosis (TB) incidence per 100,000 population (sourced from the World Bank) for measles incidence as the outcome variable. Model Q includes contemporaneous battle-related deaths (BRDs) only; Model R additionally incorporates one-year lagged BRDs. The large standardized coefficient between Socioeconomic Status and TB incidence (β=−0.82) reflects the high sensitivity of tuberculosis to structural determinants of health, including housing quality, nutritional status, and healthcare access. In contrast to measles, which is driven by discrete immunization events, TB is widely recognized as a "disease of poverty" with incidence rates that closely track national development indices. Furthermore, as a latent variable model, the structural paths in this SEM are corrected for measurement error in the indicators (gross domestic product [GDP], life expectancy, schooling), which typically results in larger coefficients than observed in standard regression models. We acknowledge that this estimate may also encompass unmeasured confounding from factors highly correlated with development, such as HIV prevalence and urban population density. AIC = Akaike Information Criterion; BIC = Bayesian Information Criterion; BRDs = battle-related deaths; CFI = Comparative Fit Index; TB = Tuberculosis; TLI = Tucker-Lewis Index; RMSEA = Root Mean Square Error of Approximation; SRMR = Standardized Root Mean Square Residual.
