# Supplementary material for: Association of armed conflict and global measles cases: A structural equation modeling analysis of 193 countries from 2000 to 2023
Source: PLoS Med. 2026 Jun 25;23(6):e1004819. doi: 10.1371/journal.pmed.1004819 (PMC13298743; doi:10.1371/journal.pmed.1004819)
Supplement: S5 Table — Models S and T use standardized total measles cases as the outcome; Models U and V use measles incidence per million population. Models U and V additionally include 1-year lagged battle-related deaths (BRDs). AIC = Akaike Information Criterion; BIC = Bayesian Information Criterion; BRDs = Battle-related deaths; CFI = Comparative Fit Index; TLI = Tucker–Lewis Index; RMSEA = Root Mean Square Error of Approximation; SRMR = Standardized Root Mean Square Residual. (DOCX) [file pmed.1004819.s012.docx]

S5 Table. Structural equation model results using Inverse Hyperbolic Sine (IHS) transformations in place of log(x+1) transformations (Models S–V), 2000–2023.

| Effect | Model S | Model T | Model U | Model V |
| --- | --- | --- | --- | --- |
| GDP per capita (IHS) → Socioeconomic development | 0.94 [0.93, 0.95]*** | 0.94 [0.93, 0.95]*** | 0.94 [0.93, 0.94]*** | 0.94 [0.93, 0.95]*** |
| Life expectancy → Socioeconomic development | 0.87 [0.86, 0.88]*** | 0.87 [0.86, 0.88]*** | 0.88 [0.87, 0.89]*** | 0.87 [0.86, 0.89]*** |
| Mean years of schooling → Socioeconomic development | 0.83 [0.82, 0.84]*** | 0.83 [0.82, 0.84]*** | 0.83 [0.82, 0.85]*** | 0.84 [0.83, 0.85]*** |
| Population displacement (%) → Socioeconomic development | -0.20 [-0.23, -0.17]*** | -0.20 [-0.23, -0.17]*** | -0.20 [-0.23, -0.17]*** | -0.20 [-0.23, -0.17]*** |
| BRDs, IHS-transformed → Socioeconomic development | -0.10 [-0.13, -0.07]*** | -0.04 [-0.11, 0.03] | -0.10 [-0.13, -0.07]*** | -0.04 [-0.11, 0.03] |
| BRDs, IHS-transformed → Population displacement (%) | 0.36 [0.31, 0.41]*** | 0.14 [0.04, 0.24]** | 0.36 [0.31, 0.41]*** | 0.14 [0.04, 0.24]** |
| Socioeconomic development → Measles cases (IHS-transformed) | -0.33 [-0.36, -0.31]*** | -0.32 [-0.34, -0.29]*** | NA | NA |
| BRDs, IHS-transformed → Measles cases (IHS-transformed) | 0.17 [0.14, 0.20]*** | 0.05 [-0.01, 0.11]. | NA | NA |
| Population displacement (%) → Measles cases (IHS-transformed) | -0.01 [-0.03, 0.01] | -0.02 [-0.04, 0.01] | NA | NA |
| BRDs, IHS-transformed (1-year lag) → Socioeconomic development | NA | -0.07 [-0.14, 0.00]. | NA | -0.07 [-0.14, 0.00]. |
| BRDs, IHS-transformed (1-year lag) → Population displacement (%) | NA | 0.27 [0.17, 0.37]*** | NA | 0.27 [0.17, 0.37]*** |
| BRDs, IHS-transformed (1-year lag) → BRDs (IHS-transformed) | NA | 0.90 [0.88, 0.92]*** | NA | 0.90 [0.88, 0.92]*** |
| BRDs, IHS-transformed (1-year lag) → Measles cases (IHS-transformed) | NA | 0.14 [0.08, 0.19]*** | NA | NA |
| Socioeconomic development → Measles incidence per million (IHS-transformed) | NA | NA | -0.35 [-0.38, -0.32]*** | -0.34 [-0.37, -0.30]*** |
| BRDs, IHS-transformed → Measles incidence per million (IHS-transformed) | NA | NA | 0.04 [0.01, 0.06]** | -0.01 [-0.07, 0.06] |
| Population displacement (%) → Measles incidence per million (IHS-transformed) | NA | NA | 0.06 [0.03, 0.10]*** | 0.06 [0.03, 0.09]*** |
| BRDs, IHS-transformed (1-year lag) → Measles incidence per million (IHS-transformed) | NA | NA | NA | 0.05 [-0.01, 0.11] |
| cfi | 0.993 | 0.996 | 0.991 | 0.994 |
| tli | 0.983 | 0.989 | 0.976 | 0.984 |
| rmsea | 0.052 | 0.047 | 0.062 | 0.056 |
| srmr | 0.012 | 0.014 | 0.014 | 0.015 |
| aic | 52387.407 | 55160.747 | 52251.705 | 55025.217 |
| bic | 52509.782 | 55320.702 | 52374.079 | 55185.172 |

**Note:** Structural equation models (SEMs) estimated standardized effects. The authors note that this analysis was added in response to peer review, and was therefore data-driven rather than planned prospectively. Models S and T use standardized total measles cases as the outcome; Models U and V use measles incidence per million population. Models U and V additionally include one-year lagged battle-related deaths (BRDs). Socioeconomic development is modeled as a latent construct defined by gross domestic product (GDP) per capita, life expectancy, and mean years of schooling. Values represent standardized path coefficients with 95% confidence intervals in brackets. Asterisks denote statistical significance (^ = *p-value* < 0.10, * = p-value < 0.05, ** = ***p-value* <** 0.01, *** = ***p-value* <** 0.001). AIC = Akaike Information Criterion; BIC = Bayesian Information Criterion; BRDs = battle-related deaths; CFI = Comparative Fit Index; TLI = Tucker-Lewis Index; RMSEA = Root Mean Square Error of Approximation; SRMR = Standardized Root Mean Square Residual.
