# Supplementary material for: Association of armed conflict and global measles cases: A structural equation modeling analysis of 193 countries from 2000 to 2023
Source: PLoS Med. 2026 Jun 25;23(6):e1004819. doi: 10.1371/journal.pmed.1004819 (PMC13298743; doi:10.1371/journal.pmed.1004819)
Supplement: S6 Table — AIC = Akaike Information Criterion; BIC = Bayesian Information Criterion; BRDs = Battle-related deaths; CFI = Comparative Fit Index; TLI = Tucker–Lewis Index; RMSEA = Root Mean Square Error of Approximation; SRMR = Standardized Root Mean Square Residual. (DOCX) [file pmed.1004819.s013.docx]

S6 Table. Structural equation model results testing bidirectional and correlated-error specifications, 2000–2023.

| Effect | Model with Bidirectionality Lag | Model with Correlated Errors |
| --- | --- | --- |
| GDP per capita → Socioeconomic development | 0.94 [0.93, 0.95]*** | 0.94 [0.93, 0.95]*** |
| Life expectancy → Socioeconomic development | 0.87 [0.86, 0.88]*** | 0.87 [0.86, 0.88]*** |
| Mean years of schooling → Socioeconomic development | 0.84 [0.83, 0.85]*** | 0.83 [0.82, 0.84]*** |
| Population displacement (%) → Socioeconomic development | -0.20 [-0.23, -0.17]*** | -0.20 [-0.23, -0.17]*** |
| BRDs → Socioeconomic development | -0.09 [-0.13, -0.06]*** | -0.10 [-0.13, -0.07]*** |
| BRDs → Population displacement (%) | 0.38 [0.33, 0.43]*** | 0.37 [0.32, 0.42]*** |
| Socioeconomic development → Measles cases | -0.34 [-0.37, -0.31]*** | -0.34 [-0.37, -0.31]*** |
| BRDs → Measles cases | 0.17 [0.14, 0.21]*** | 0.21 [0.11, 0.30]*** |
| Population displacement (%) → Measles cases | -0.02 [-0.04, 0.01] | -0.11 [-0.34, 0.13] |
| Measles cases (1-year lag) → Population displacement (%) | 0.04 [0.02, 0.07]** | NA |
| Measles cases (1-year lag) → BRDs | 0.22 [0.19, 0.25]*** | NA |
| CFI | 0.698 | 0.993 |
| TLI | 0.365 | 0.98 |
| RMSEA | 0.333 | 0.057 |
| SRMR | 0.156 | 0.012 |
| AIC | 58509.048 | 52346.416 |
| BIC | 58654.491 | 52475.231 |

**Note:** Structural equation models (SEMs) estimated standardized effects. The model with bidirectional lags assesses reciprocal temporal influences between variables over time, while the model with correlated errors accounts for unmeasured shared variance between contemporaneous error terms. Values represent standardized path coefficients with 95% confidence intervals in brackets. Asterisks denote statistical significance (^ = *p-value* < 0.10, * = p-value < 0.05, ** = ***p-value* <** 0.01, *** = ***p-value* <** 0.001). Socioeconomic development is a latent construct defined by gross domestic product (GDP) per capita, life expectancy, and mean years of schooling. AIC = Akaike Information Criterion; BIC = Bayesian Information Criterion; BRDs = battle-related deaths; CFI = Comparative Fit Index; TLI = Tucker-Lewis Index; RMSEA = Root Mean Square Error of Approximation; SRMR = Standardized Root Mean Square Residual.
