# Supplementary material for: Association of armed conflict and global measles cases: A structural equation modeling analysis of 193 countries from 2000 to 2023
Source: PLoS Med. 2026 Jun 25;23(6):e1004819. doi: 10.1371/journal.pmed.1004819 (PMC13298743; doi:10.1371/journal.pmed.1004819)
Supplement: S7 Table — The full first-order autoregressive [AR(1)] model accounts for first-order serial dependence by including autoregressive paths for both exposure and outcome variables, whereas the outcome-only model restricts these paths to the dependent variable alone. AIC = Akaike Information Criterion; BIC = Bayesian Information Criterion; BRDs = Battle-related deaths; CFI = Comparative Fit Index; TLI = Tucker–Lewis Index; RMSEA = Root Mean Square Error of Approximation; SRMR = Standardized Root Mean Square Residual. (DOCX) [file pmed.1004819.s014.docx]

S7 Table. Structural equation model results with explicit modeling of first-order autoregressive [AR(1)] serial dependence, 2000–2023.

| Effect | Full AR(1) Model | Outcome-Only AR(1) Model |
| --- | --- | --- |
| GDP per capita → Socioeconomic development | 0.94 [0.93, 0.95]*** | 0.94 [0.93, 0.95]*** |
| Life expectancy → Socioeconomic development | 0.87 [0.86, 0.88]*** | 0.88 [0.86, 0.89]*** |
| Mean years of schooling → Socioeconomic development | 0.83 [0.82, 0.84]*** | 0.84 [0.83, 0.85]*** |
| Population displacement (%) → Socioeconomic development | -0.20 [-0.23, -0.17]*** | -0.20 [-0.25, -0.16]*** |
| BRDs → Socioeconomic development | -0.10 [-0.13, -0.07]*** | -0.09 [-0.13, -0.06]*** |
| BRDs → Population displacement (%) | 0.06 [0.02, 0.09]** | 0.06 [0.02, 0.09]** |
| Socioeconomic development → Measles cases | -0.07 [-0.09, -0.05]*** | -0.06 [-0.08, -0.04]*** |
| BRDs → Measles cases | 0.04 [0.02, 0.06]*** | 0.04 [0.02, 0.06]*** |
| Population displacement (%) → Measles cases | 0.00 [-0.02, 0.02] | 0.00 [-0.02, 0.02] |
| BRDs (1-year lag) → BRDs | 0.37 [0.03, 0.70]* | 0.90 [0.88, 0.92]*** |
| Population displacement, 1-year lag (%) → Population displacement (%) | 0.00 [-0.14, 0.14] | 0.94 [0.89, 0.98]*** |
| Measles cases (1-year lag) → Measles cases | 0.58 [0.56, 0.59]*** | 0.14 [0.11, 0.16]*** |
| CFI | 0.957 | 0.977 |
| TLI | 0.926 | 0.964 |
| RMSEA | 0.123 | 0.088 |
| SRMR | 0.153 | 0.114 |
| AIC | 54839.301 | 50777.64 |
| BIC | 55051.845 | 50948.98 |

**Note:** Structural equation models (SEMs) estimated standardized effects. The authors note that this analysis was added in response to peer review, and was therefore data-driven rather than planned prospectively. The full first-order autoregressive [AR(1)] model accounts for first-order serial dependence by including autoregressive paths for both exposure and outcome variables, whereas the outcome-only model restricts these paths to the dependent variable alone. Values represent standardized path coefficients with 95% confidence intervals in brackets. Asterisks denote statistical significance (^ = *p-value* < 0.10, * = p-value < 0.05, ** = ***p-value* <** 0.01, *** = ***p-value* <** 0.001). Socioeconomic development is a latent construct defined by gross domestic product (GDP) per capita, life expectancy, and mean years of schooling. AIC = Akaike Information Criterion; BIC = Bayesian Information Criterion; BRDs = battle-related deaths; CFI = Comparative Fit Index; TLI = Tucker-Lewis Index; RMSEA = Root Mean Square Error of Approximation; SRMR = Standardized Root Mean Square Residual.
