# Supplementary material for: Association of armed conflict and global measles cases: A structural equation modeling analysis of 193 countries from 2000 to 2023
Source: PLoS Med. 2026 Jun 25;23(6):e1004819. doi: 10.1371/journal.pmed.1004819 (PMC13298743; doi:10.1371/journal.pmed.1004819)
Supplement: S8 Table — FIML = Full Information Maximum Likelihood. (DOCX) [file pmed.1004819.s015.docx]

S8 Table. Structural path coefficients under alternative missing data specifications (Model A), 2000–2023.

| **Path** | **Primary (FIML)** | **Auxiliary Var** | **High-Quality Subset** | **Multiple Imputation** |
| --- | --- | --- | --- | --- |
| **SocioEcon ~ Displacement** | -0.20*** | -0.21*** | -0.18*** | -0.19*** |
| **SocioEcon ~ Battle Deaths** | -0.10*** | -0.10*** | -0.10*** | -0.09*** |
| **Measles ~ SocioEcon** | -0.34*** | -0.34*** | -0.34*** | -0.36*** |
| **Measles ~ Battle Deaths** | 0.18*** | 0.18*** | 0.17*** | 0.17*** |
| *Missingness Treatment* | *FIML* | *FIML + Aux* | *Complete Cases* | *MICE (m=5)* |

**Note:** Primary (FIML) utilizes Full Information Maximum Likelihood (FIML) estimation to maximize the likelihood function for all available data, assuming data are Missing at Random (MAR) conditional on observed covariates. Auxiliary Variable structural equation modeling (SEM) incorporates human development index (HDI) and total population to further satisfy the MAR assumption and mitigate non-reporting bias. High-Quality Subset restricts the analysis to countries with ≥80% data completeness to assess sensitivity to data uncertainty. Multiple Imputation pools estimates across m=5 datasets generated via Multiple Imputation by Chained Equations (MICE) using Predictive Mean Matching (PMM). All coefficients are standardized. SocioEcon represents a latent construct defined by gross domestic product (GDP) per capita, mean years of schooling, and life expectancy. Significance levels: * p<0.05, ** p<0.01, *** p<0.001.
