# Supplementary material for: Association of armed conflict and global measles cases: A structural equation modeling analysis of 193 countries from 2000 to 2023
Source: PLoS Med. 2026 Jun 25;23(6):e1004819. doi: 10.1371/journal.pmed.1004819 (PMC13298743; doi:10.1371/journal.pmed.1004819)
Supplement: S9 Table — Models W and X use standardized total measles cases as the outcome; Models Y and Z use measles incidence per million population. Models X and Z additionally include 1-year lagged battle-related deaths (BRDs). AIC = Akaike Information Criterion; BIC = Bayesian Information Criterion; BRDs = Battle-related deaths; CFI = Comparative Fit Index; TLI = Tucker–Lewis Index; RMSEA = Root Mean Square Error of Approximation; SE = Standard Error; SRMR = Standardized Root Mean Square Residual. (DOCX) [file pmed.1004819.s016.docx]

S9 Table. Structural equation model results with cluster-robust standard errors adjusted for within-country correlation (Models W-Z), 2000–2023.

| Effect | W (Model A with Cluster-Robust SEs) | X (Model B with Cluster-Robust SEs) | Y (Model C with Cluster-Robust SEs) | Z (Model D with Cluster-Robust SEs) |
| --- | --- | --- | --- | --- |
| GDP per capita → Socioeconomic development | 0.94 [0.91, 0.97]*** | 0.94 [0.91, 0.97]*** | 0.94 [0.90, 0.97]*** | 0.94 [0.91, 0.97]*** |
| Life expectancy → Socioeconomic development | 0.87 [0.84, 0.91]*** | 0.87 [0.83, 0.91]*** | 0.88 [0.84, 0.92]*** | 0.87 [0.84, 0.91]*** |
| Mean years of schooling → Socioeconomic development | 0.83 [0.79, 0.88]*** | 0.83 [0.79, 0.88]*** | 0.83 [0.79, 0.88]*** | 0.84 [0.79, 0.88]*** |
| Population displacement (%) → Socioeconomic development | -0.20 [-0.31, -0.10]*** | -0.20 [-0.31, -0.09]*** | -0.20 [-0.31, -0.10]*** | -0.20 [-0.31, -0.09]*** |
| BRDs → Socioeconomic development | -0.10 [-0.22, 0.02]. | -0.04 [-0.11, 0.03] | -0.10 [-0.22, 0.02]. | -0.04 [-0.11, 0.03] |
| BRDs → Population displacement (%) | 0.37 [0.20, 0.54]*** | 0.13 [0.05, 0.22]** | 0.37 [0.20, 0.54]*** | 0.13 [0.05, 0.22]** |
| Socioeconomic development → Measles cases | -0.34 [-0.43, -0.25]*** | -0.32 [-0.42, -0.23]*** | NA | NA |
| BRDs → Measles cases | 0.17 [0.08, 0.27]*** | 0.05 [-0.01, 0.11] | NA | NA |
| Population displacement (%) → Measles cases | -0.01 [-0.07, 0.05] | -0.02 [-0.09, 0.05] | NA | NA |
| BRDs (1-year lag) → Socioeconomic development | NA | -0.07 [-0.14, 0.01]. | NA | -0.07 [-0.14, 0.01]. |
| BRDs (1-year lag) → Population displacement (%) | NA | 0.28 [0.15, 0.41]*** | NA | 0.28 [0.15, 0.41]*** |
| BRDs (1-year lag) → BRDs | NA | 0.90 [0.87, 0.94]*** | NA | 0.90 [0.87, 0.94]*** |
| BRDs (1-year lag) → Measles cases | NA | 0.14 [0.07, 0.21]*** | NA | NA |
| Socioeconomic development → Measles incidence per million | NA | NA | -0.36 [-0.44, -0.28]*** | -0.34 [-0.42, -0.26]*** |
| BRDs → Measles incidence per million | NA | NA | 0.03 [-0.03, 0.10] | -0.00 [-0.06, 0.06] |
| Population displacement (%) → Measles incidence per million | NA | NA | 0.06 [-0.00, 0.13]. | 0.06 [-0.01, 0.13]. |
| BRDs (1-year lag) → Measles incidence per million | NA | NA | NA | 0.04 [-0.02, 0.10] |
| CFI | 0.993 | 0.996 | 0.991 | 0.994 |
| TLI | 0.983 | 0.989 | 0.976 | 0.984 |
| RMSEA | 0.052 | 0.047 | 0.062 | 0.056 |
| SRMR | 0.012 | 0.014 | 0.014 | 0.015 |
| AIC | 52344.416 | 54974.25 | 52218.541 | 54842.276 |
| BIC | 52466.79 | 55134.204 | 52340.915 | 55002.23 |

**Note:** Structural equation models (SEMs) estimated standardized effects with cluster-robust standard errors (SEs). The authors note that this analysis was added in response to peer review, and was therefore data-driven rather than planned prospectively. Models W and X use standardized total measles cases as the outcome; Models Y and Z use measles incidence per million population. Models X and Z additionally include one-year lagged battle-related deaths (BRDs). Socioeconomic development is modeled as a latent construct defined by gross domestic product (GDP) per capita, life expectancy, and mean years of schooling. Values represent standardized path coefficients with 95% confidence intervals in brackets. Asterisks denote statistical significance (^ = *p-value* < 0.10, * = p-value < 0.05, ** = ***p-value* <** 0.01, *** = ***p-value* <** 0.001). AIC = Akaike Information Criterion; BIC = Bayesian Information Criterion; BRDs = battle-related deaths; CFI = Comparative Fit Index; TLI = Tucker-Lewis Index; RMSEA = Root Mean Square Error of Approximation; SE = Standard Error; SRMR = Standardized Root Mean Square Residual.
