# Supplementary material for: Association of armed conflict and global measles cases: A structural equation modeling analysis of 193 countries from 2000 to 2023
Source: PLoS Med. 2026 Jun 25;23(6):e1004819. doi: 10.1371/journal.pmed.1004819 (PMC13298743; doi:10.1371/journal.pmed.1004819)
Supplement: S10 Table — CFI = Comparative Fit Index; RMSEA = Root Mean Square Error of Approximation; SRMR = Standardized Root Mean Square Residual. (DOCX) [file pmed.1004819.s017.docx]

S10 Table. First-difference structural equation model results examining year-over-year changes in armed conflict, population displacement, socioeconomic development, and measles incidence, 2001–2023.

| **Path** | **Standardized β** | **z-value** | **p-value** |
| --- | --- | --- | --- |
| $\Delta$Displacement $\to$ $\Delta$SocioEcon | -0.37 | -2.52 | 0.012 |
| $\boldsymbol{\Delta}$Battle Deaths $\boldsymbol{\to}\boldsymbol{\Delta}$Displacement | 0.04 | 1.12 | 0.263 |
| $\boldsymbol{\Delta}$SocioEcon $\boldsymbol{\to}$ $\boldsymbol{\Delta}$Measles Incidence | 0.04 | 0.60 | 0.547 |
| $\boldsymbol{\Delta}$Displacement $\boldsymbol{\to}\boldsymbol{\Delta}$Measles Incidence | 0.04 | 1.67 | 0.095 |
| **Model Fit Indices** | **Value** |  |  |
| RMSEA (Robust) | $0.024$ |  |  |
| SRMR | $0.013$ |  |  |
| CFI (Robust) | $0.855$ |  |  |

**Note**: First-difference structural equation models (SEMs) estimate the standardized effects of year-over-year changes in variables using cluster-robust standard errors. Socioeconomic development is modeled as a latent construct defined by gross domestic product (GDP) per capita, life expectancy, and mean years of schooling. Values represent standardized path coefficients (β), z-values, and p-values. The authors note that this analysis was added in response to peer review, and was therefore data-driven rather than planned prospectively. CFI = Comparative Fit Index; RMSEA = Root Mean Square Error of Approximation; SRMR = Standardized Root Mean Square Residual.
