# Supplementary material for: Association of armed conflict and global measles cases: A structural equation modeling analysis of 193 countries from 2000 to 2023
Source: PLoS Med. 2026 Jun 25;23(6):e1004819. doi: 10.1371/journal.pmed.1004819 (PMC13298743; doi:10.1371/journal.pmed.1004819)
Supplement: S11 Table — AIC = Akaike Information Criterion; BIC = Bayesian Information Criterion; BRDs = Battle-related deaths; CFI = Comparative Fit Index; TLI = Tucker–Lewis Index; RMSEA = Root Mean Square Error of Approximation; SE = Standard Error; SRMR = Standardized Root Mean Square Residual. (DOCX) [file pmed.1004819.s018.docx]

S11 Table. Extended lag analysis: distributed lag structural equation model results examining conflict effects on measles outcomes over a 3-year temporal horizon (Models AA and BB), 2003–2023.

| Effect | AA (Measles Count) | BB (Measles Incidence) |
| --- | --- | --- |
| GDP per capita → Socioeconomic development | 0.94 [0.94, 0.95]*** | 0.94 [0.93, 0.95]*** |
| Life expectancy → Socioeconomic development | 0.87 [0.86, 0.88]*** | 0.87 [0.86, 0.88]*** |
| Mean years of schooling → Socioeconomic development | 0.84 [0.83, 0.85]*** | 0.84 [0.83, 0.85]*** |
| Population displacement (%) → Socioeconomic development | -0.21 [-0.24, -0.17]*** | -0.21 [-0.24, -0.17]*** |
| BRDs → Socioeconomic development | -0.04 [-0.12, 0.04] | -0.04 [-0.12, 0.04] |
| BRDs (1-year lag) → Socioeconomic development | -0.01 [-0.10, 0.09] | -0.01 [-0.10, 0.09] |
| BRDs (2-year lag) → Socioeconomic development | -0.00 [-0.09, 0.09] | -0.00 [-0.09, 0.09] |
| BRDs (3-year lag) → Socioeconomic development | -0.06 [-0.14, 0.02] | -0.06 [-0.14, 0.02] |
| BRDs → Population displacement (%) | 0.07 [-0.06, 0.20] | 0.07 [-0.06, 0.20] |
| BRDs (1-year lag) → Population displacement (%) | 0.17 [0.02, 0.31]* | 0.17 [0.02, 0.31]* |
| BRDs (2-year lag) → Population displacement (%) | 0.11 [-0.00, 0.22]. | 0.11 [-0.00, 0.22]. |
| BRDs (3-year lag) → Population displacement (%) | 0.10 [-0.04, 0.24] | 0.10 [-0.04, 0.24] |
| Socioeconomic development → Measles cases | -0.29 [-0.32, -0.26]*** | NA |
| BRDs → Measles cases | 0.03 [-0.04, 0.09] | NA |
| BRDs (1-year lag) → Measles cases | 0.07 [-0.00, 0.14]. | NA |
| BRDs (2-year lag) → Measles cases | 0.01 [-0.06, 0.08] | NA |
| BRDs (3-year lag) → Measles cases | 0.09 [0.03, 0.15]** | NA |
| Socioeconomic development → Measles incidence per million | NA | -0.32 [-0.35, -0.29]*** |
| BRDs → Measles incidence per million | NA | -0.00 [-0.07, 0.07] |
| BRDs (1-year lag) → Measles incidence per million | NA | 0.04 [-0.03, 0.11] |
| BRDs (2-year lag) → Measles incidence per million | NA | 0.01 [-0.06, 0.08] |
| BRDs (3-year lag) → Measles incidence per million | NA | 0.03 [-0.03, 0.09] |
| CFI | 0.992 | 0.987 |
| TLI | 0.982 | 0.97 |
| RMSEA | 0.039 | 0.05 |
| SRMR | 0.015 | 0.018 |
| AIC | 45195.073 | 45048.048 |
| BIC | 45365.368 | 45218.343 |
| Wald p-value | 0.002 | 0.298 |

**Note:** Structural equation models (SEMs) estimated standardized effects. Values represent standardized path coefficients with 95% confidence intervals in brackets. Asterisks denote statistical significance (^ = *p-value* < 0.10, * = p-value < 0.05, ** = ***p-value* <** 0.01, *** = ***p-value* <** 0.001). Socioeconomic development is a latent construct defined by gross domestic product (GDP) per capita, life expectancy, and mean years of schooling. $\mathbf{Wald Test (}\boldsymbol{BD}_{\boldsymbol{t}\boldsymbol{0}} \mathbf{to} \boldsymbol{BD}_{\boldsymbol{t-3}}\mathbf{):}\mathbf{x}^{\mathbf{2}}(4)=17.03, p =0.0019 (Model AA);\mathbf{x}^{\mathbf{2}}(4)=4.90, p=0.2979 (Model BB).$ The authors note that this analysis was added in response to peer review, and was therefore data-driven rather than planned prospectively. AIC = Akaike Information Criterion; BIC = Bayesian Information Criterion; BRDs = battle-related deaths; CFI = Comparative Fit Index; TLI = Tucker-Lewis Index; RMSEA = Root Mean Square Error of Approximation; SE = Standard Error; SRMR = Standardized Root Mean Square Residual.
