# Supplementary material for: Association of armed conflict and global measles cases: A structural equation modeling analysis of 193 countries from 2000 to 2023
Source: PLoS Med. 2026 Jun 25;23(6):e1004819. doi: 10.1371/journal.pmed.1004819 (PMC13298743; doi:10.1371/journal.pmed.1004819)
Supplement: S12 Table — CI = confidence interval; GDP = gross domestic product. (DOCX) [file pmed.1004819.s019.docx]

S12 Table. Parallel mediation analysis disaggregating socioeconomic development into economic level (gross domestic product [GDP] per capita) and human capital (life expectancy and mean years of schooling), 2000–2023.

| **Path** | **Standardized Estimate [95% CI]** | **P-value** |
| --- | --- | --- |
| **Direct Effects on Measles** |  |  |
| Economic Level (GDP) $\boldsymbol{\to}$ Measles | -0.18 [-0.35, -0.01] | 0.038 |
| Human Capital $\boldsymbol{\to}$Measles | -0.18 [-0.36, 0.00] | 0.050 |
| Battle Deaths $\boldsymbol{\to}$ Measles | 0.17 [0.07, 0.27] | 0.001 |
| Displacement (%) $\boldsymbol{\to}$Measles | -0.01 [-0.07, 0.04] | 0.665 |
| **Determinants of Economic/Human Capital** |  |  |
| Displacement $\boldsymbol{\to}$Economic Level | -0.22 [-0.38, -0.06] | 0.009 |
| Displacement $\boldsymbol{\to}$Human Capital | -0.17 [-0.37, 0.03] | 0.080 |

**Note**: This sensitivity analysis tests the distinct mediating effects of wealth versus human development. Socioeconomic status is disaggregated into Economic Level (observed Gross Domestic Product [GDP]) and Human Capital (a latent construct of life expectancy and schooling). Both dimensions exert nearly identical protective effects against measles (Standardized β=−0.18 for both; p≤0.05). The significant direct effect of battle deaths (β=0.17, p=0.001) suggests violence impacts transmission through pathways beyond socioeconomic degradation. Standardized coefficients are reported with robust standard errors clustered by country. A second-order latent model was not implemented due to mathematical under-identification with only three available indicators. The authors note that this analysis was added in response to peer review, and was therefore data-driven rather than planned prospectively. CI = confidence interval; GDP = gross domestic product.
