# Supplementary material for: Association of armed conflict and global measles cases: A structural equation modeling analysis of 193 countries from 2000 to 2023
Source: PLoS Med. 2026 Jun 25;23(6):e1004819. doi: 10.1371/journal.pmed.1004819 (PMC13298743; doi:10.1371/journal.pmed.1004819)
Supplement: S13 Table — Models A and B use standardized total measles cases as the outcome; Models C and D use measles incidence per million population. Models B and D additionally include 1-year lagged battle-related deaths (BRDs). BRD = Battle-related deaths; Disp = displacement; SE = socioeconomic development; Socioecon = socioeconomic development. (DOCX) [file pmed.1004819.s020.docx]

S13 Table. Hierarchical decomposition of the total effect of armed conflict on measles burden into direct and indirect pathways (Models A–D), 2000–2023.

| **Pathway / Model** | **Model A (Cases)** | **Model B (Cases+Lags)** | **Model C (Rate)** | **Model D (Rate+Lags)** |
| --- | --- | --- | --- | --- |
| **Direct Effect** (BRD $\boldsymbol{\to}$ Measles) | 0.170 (72.9%) | 0.190 (73.4%) | 0.030 (26.1%) | 0.06 (42.6%) |
| **Indirect via Socioecon** | 0.034 (15.2%) | 0.035 (13.5%) | 0.036 (31.3%) | 0.037 (26.2%) |
| **Indirect via Displacement** | 0.004 (1.7%) | 0.008 (3.1%) | 0.022 (19.1%) | 0.016 (11.3%) |
| **Compound** (Disp$\boldsymbol{\to}$SE$\boldsymbol{\to}$Measles) | 0.025 (10.7%) | 0.026 (10.0%) | 0.027 (23.5%) | 0.028 (19.9%) |
| **Total Effect (Sum)** | **0.233 (100%)** | **0.259 (100%)** | **0.115 (100%)** | **0.141 (100%)** |

**Note**: Models A and B use standardized total measles cases as the outcome; Models C and D use measles incidence per million population. Models B and D additionally include one-year lagged battle-related deaths (BRDs). Contribution percentages are calculated based on the absolute standardized magnitude of each pathway relative to the total estimated influence. Socioeconomic development is a latent construct defined by gross domestic product (GDP) per capita, life expectancy, and mean years of schooling. The authors note that this analysis was added in response to peer review, and was therefore data-driven rather than planned prospectively. BRD = battle-related deaths; Disp = displacement; SE = socioeconomic development; Socioecon = socioeconomic development.
