# Supplementary material for: Association of armed conflict and global measles cases: A structural equation modeling analysis of 193 countries from 2000 to 2023
Source: PLoS Med. 2026 Jun 25;23(6):e1004819. doi: 10.1371/journal.pmed.1004819 (PMC13298743; doi:10.1371/journal.pmed.1004819)
Supplement: S1 Checklist — Reproduced under the Creative Commons Attribution 4.0 International License (CC BY 4.0). Available from: https://www.strobe-statement.org/. (DOCX) [file pmed.1004819.s021.docx]

**STROBE Statement — Checklist of items that should be included in reports of cross-sectional studies.** Reproduced under the Creative Commons Attribution 4.0 International License (CC BY 4.0). Available from: <https://www.strobe-statement.org/>

*Manuscript: Association of armed conflict and global measles cases: A structural equation modeling analysis of 193 countries from 2000 to 2023.*

|  | **Item No** | **Recommendation** | **Location in manuscript** |
| --- | --- | --- | --- |
| **Title and abstract** | 1 | (a) Indicate the study's design with a commonly used term in the title or the abstract | Title; Abstract (Methods and findings) |
|  |  | (b) Provide in the abstract an informative and balanced summary of what was done and what was found | Abstract (Background, Methods and findings, Conclusions) |
| **Introduction** | | | |
| **Background/rationale** | 2 | Explain the scientific background and rationale for the investigation being reported | Introduction (Paras 1-2) |
| **Objectives** | 3 | State specific objectives, including any prespecified hypotheses | Introduction (Para 3); Methods (Hypotheses and analytical plan, Para 1) |
| **Methods** | | | |
| **Study design** | 4 | Present key elements of study design early in the paper | Methods (Para 1) |
| **Setting** | 5 | Describe the setting, locations, and relevant dates, including periods of recruitment, exposure, follow-up, and data collection | Methods (Para 1); Methods (Data, Paras 1–5) |
| **Participants** | 6 | (a) Give the eligibility criteria, and the sources and methods of selection of participants | Methods (Para 1); Methods (Data, Paras 1–5) |
| **Variables** | 7 | Clearly define all outcomes, exposures, predictors, potential confounders, and effect modifiers. Give diagnostic criteria, if applicable | Methods (Data, Paras 1–5); Methods (Statistical analysis, Paras 5–6) |
| **Data sources / measurement** | 8* | For each variable of interest, give sources of data and details of methods of assessment (measurement). Describe comparability of assessment methods if there is more than one group | Methods (Data, Paras 1–5) |
| **Bias** | 9 | Describe any efforts to address potential sources of bias | Methods (Statistical analysis, Paras 2–4 and 7); Methods (Robustness, Para 1); Discussion (Paras 5–8) |
| **Study size** | 10 | Explain how the study size was arrived at | Methods, (Para 1); Results (Descriptive analysis, Para 1) |
| **Quantitative variables** | 11 | Explain how quantitative variables were handled in the analyses. If applicable, describe which groupings were chosen and why | Methods (Statistical analysis, Para 1) |
| **Statistical methods** | 12 | (a) Describe all statistical methods, including those used to control for confounding | Methods (Statistical analysis, Paras 2–8); Methods (Robustness, Para 1) |
|  |  | (b) Describe any methods used to examine subgroups and interactions | NA (no subgroup or interaction analyses were analyzed outside fixed-effects). |
|  |  | (c) Explain how missing data were addressed | Methods (Statistical analysis, Para 7) |
|  |  | (d) If applicable, describe analytical methods taking account of sampling strategy | NA (population-level country-year data rather than a survey sample) |
|  |  | (e) Describe any sensitivity analyses | Methods (Robustness, Para 1); Methods (Statistical analysis Paras 1 and 7) |
| **Results** | | | |
| **Participants** | 13* | (a) Report numbers of individuals at each stage of study—e.g., numbers potentially eligible, examined for eligibility, confirmed eligible, included in the study, completing follow-up, and analysed | Results (Descriptive analysis, Para 1) |
|  |  | (b) Give reasons for non-participation at each stage | Methods, (Para 1); Methods (Statistical analysis, Para 7) |
|  |  | (c) Consider use of a flow diagram | NA |
| **Descriptive data** | 14* | (a) Give characteristics of study participants (e.g., demographic, clinical, social) and information on exposures and potential confounders | Results (Descriptive analysis, Para 1) |
|  |  | (b) Indicate number of participants with missing data for each variable of interest | Results (Table 1) |
| **Outcome data** | 15* | Report numbers of outcome events or summary measures | Results (Descriptive analysis, Para 1) Results (Table 1) |
| **Main results** | 16 | (a) Give unadjusted estimates and, if applicable, confounder-adjusted estimates and their precision (e.g., 95% confidence interval). Make clear which confounders were adjusted for and why they were included | Results (Table 2); Results (Table 3); Results (Regression analysis, Para 1); Results (Structural equation modeling, Paras 1-9) |
|  |  | (b) Report category boundaries when continuous variables were categorized | NA (all continuous variables analyzed as continuous following transformation and z-score standardization) |
|  |  | (c) If relevant, consider translating estimates of relative risk into absolute risk for a meaningful time period | Abstract (Methods and findings, Para 1); Discussion (Para 1) |
| **Other analyses** | 17 | Report other analyses done—e.g., analyses of subgroups and interactions, and sensitivity analyses | Results (Robustness analysis, Paras 1–4); Supplementary Information (S1–S13 Tables; S3–S6 Figs) |
| **Discussion** | | | |
| **Key results** | 18 | Summarise key results with reference to study objectives | Discussion (Paras 1–2) |
| **Limitations** | 19 | Discuss limitations of the study, taking into account sources of potential bias or imprecision. Discuss both direction and magnitude of any potential bias | Discussion (Paras 5–8) |
| **Interpretation** | 20 | Give a cautious overall interpretation of results considering objectives, limitations, multiplicity of analyses, results from similar studies, and other relevant evidence | Discussion, (Paras 2–4 and 9) |
| **Generalisability** | 21 | Discuss the generalisability (external validity) of the study results | Discussion (Paras 5–6 and 9) |
| **Other information** | | | |
| **Funding** | 22 | Give the source of funding and the role of the funders for the present study and, if applicable, for the original study on which the present article is based | Financial Disclosure / Funding statement; Acknowledgments |

** Give information separately for exposed and unexposed groups.*
