# Supplementary material for: Association of armed conflict and global measles cases: A structural equation modeling analysis of 193 countries from 2000 to 2023
Source: PLoS Med. 2026 Jun 25;23(6):e1004819. doi: 10.1371/journal.pmed.1004819 (PMC13298743; doi:10.1371/journal.pmed.1004819)
Supplement: Attachment — Submitted filename: 20260131_PLOS Medicine_Response to Reviewers_Clean.docx [file pmed.1004819.s025.docx]

Dear Reviewers,

We would like to express our sincere gratitude for the time and intellectual rigor you dedicated to reviewing our manuscript. Your critiques, particularly regarding the nuances of causal inference, the risks of bias, and the necessity of accounting for temporal autocorrelation, have been instrumental in strengthening the paper.

In response to your feedback, we have reframed our work as an exploratory structural analysis rather than a definitive causal one. We have also added a suite of sensitivity analyses (including IHS transformations, AR(1) modeling, and Multiple Imputation) to ensure that our findings are as robust as they are theoretically grounded. We believe the revised manuscript is much more nuanced and statistically sound thanks to your guidance.

-----------------------------------------------------------
Comments from the reviewers:

Reviewer #1: Major Comments
1) The authors should clarify why SEM was preferred over more robust causal inference methods (e.g., marginal structural models, difference-in-differences with staggered adoption, dynamic panel models), given the potential for time-varying confounding and reciprocal causation. **-> We thank the reviewer for this comment. In response, we added the following rationale to the Introduction: “We used SEM as a theory-informed, exploratory framework to characterize the relative strength and interdependence of multiple hypothesized pathways linking armed conflict, displacement, socioeconomic conditions, and measles burden over time, rather than to identify causal effects. More robust causal inference approaches were not feasible given the absence of well-defined treatment onset, substantial cross-national heterogeneity, and the likelihood of reciprocal and time-varying processes that limit credible causal identification in global country-year data”** **(Lines 70-76).**

2) The manuscript states that fixed-effects regression was used to "inform SEM specification" and that SEM is used for "exploratory analysis," but the rationale for combining these two frameworks is not clearly articulated. **-> Thank you for this comment. The fixed-effect regressions served to screen robust within-country associations for individual links, while the SEM was used to integrate these links into a simultaneous system to estimate the total and indirect associations through the latent socioeconomic construct. To this end, we added the following language to the Methods: “…and were used to assess whether the observed data are compatible with hypothesized directional associations… As detailed in Table 2, this sequence was structured according to a path-building logic designed to systematically isolate the individual drivers within the broader conflict-measles framework.” (Lines 167-170).**

3) Although the authors include lagged BRDs to address simultaneity, the approach does not adequately resolve issues of temporal ordering or unmeasured confounding. SEM with contemporaneous and one-year lagged variables cannot establish causal direction in a setting with complex time-varying processes such as conflict, displacement, and health system deterioration. **-> We acknowledge the reviewer’s concern regarding the limitations of SEM models. In response to this and other reviewer comments, we have attempted to remove all causal language from the manuscript, instead framing our results as structural associations. While the inclusion of lagged battle-related deaths (BRDs) helps mitigate immediate simultaneity bias, we recognize it does not fully resolve the risks of unmeasured confounding or reverse causality (e.g., health system collapse potentially exacerbating social instability). We have added a section to the Discussion to explicitly qualify these temporal limitations and characterize our model as a test of theoretically grounded pathways rather than a definitive causal inference: “Third, endogeneity and unmeasured confounding remain inherent risks. Armed conflict is rarely a purely exogenous force; it often emerges from localized political instability that may independently degrade infrastructure prior to active fighting. Furthermore, our models do not account for time-varying factors such as humanitarian access or aid intensity. A contraction of humanitarian space may simultaneously increase outbreak risk and decrease surveillance reliability, further obscuring the true impact. Although we focus on the pathway from conflict to health, reciprocal relationships remain possible, as large-scale outbreaks can exacerbate socioeconomic instability and fuel social grievances. Consequently, our findings should be interpreted as structural associations that align with existing humanitarian theory, rather than as definitive causal evidence of directionality.” (Lines 479-488).**

4) The manuscript should clearly state the assumptions required for causal interpretation and explicitly discuss potential violations, including measurement error in BRDs and measles data, and omitted time-varying confounders (e.g., vaccination campaigns, political instability, drought/famine). **-> Thank you for this comment. We added the following language to the Methods: “SEM was selected for its ability to estimate multiple direct and indirect pathways simultaneously, incorporate latent constructs, and model mediating and covarying relationships; however, causal interpretation would require strong assumptions, including correct temporal ordering, the absence of unmeasured time-varying confounding, and minimal measurement error in key indicators. These assumptions are unlikely to be fully met in this setting given potential misclassification and misreporting of BRDs and measles cases, as well as omitted time-varying factors such as vaccination campaigns, political instability, humanitarian access, and climate-related shocks. Accordingly, consistent with ongoing debate in the literature regarding the limitations of SEM for causal attributions (18)(19), we interpret our results as exploratory associations that are informative for hypothesis generation (20).” (Lines 181-191).**

5) The analysis relies entirely on annual national aggregates, introducing substantial risk of ecological bias. Measles transmission dynamics and displacement events occur at far more granular temporal and spatial scales. The Methods should acknowledge that country-year averages may mask significant heterogeneity, and results should not be interpreted as individual-level or subnational causal effects. **-> Thank you for the opportunity to discuss the risk of ecological bias. To that end, we added the following language to the Methods: “We note that even with standardization of our variables, all analyses rely on annual national-level aggregates, which may mask substantial subnational and temporal heterogeneity in conflict, displacement, and measles transmission dynamics. Accordingly, results should be interpreted as country-level associations and not as individual-level or subnational causal effects” (Lines 151-154). We also added additional text to the Discussion in our reflection on limitations: “national-level aggregation introduces a risk of spatial dilution; by masking localized disruptions in conflict "hotspots," national averages likely bias our coefficients toward the null” (Lines 461-463).**

6 Although the latent construct loads strongly on GDP per capita, schooling, and life expectancy, these are slow-moving structural variables that may not capture short-term socioeconomic disruptions resulting from conflict. The authors should discuss whether faster-moving indicators (e.g., health expenditure, poverty headcount, night-lights data, service-delivery measures) would better reflect conflict-related socioeconomic decline. **-> We thank the reviewer for this insightful comment. We agree that while GDP, schooling, and life expectancy are robust, slow-moving indicators of development, they may lag behind the immediate, acute socioeconomic shocks caused by conflict. Our choice was primarily guided by the need for longitudinal data consistency and minimizing missingness across the study period. We also conceptualize GDP, schooling, and life expectancy not just as economic indicators, but as markers of structural resilience. While they are slow-moving, they represent the baseline stability of the health and social systems that determine a population’s vulnerability to a measles outbreak during conflict. In response, we added the following text to the Methods, “The selection of these indicators was driven by their status as standardized, internationally comparable metrics of structural development. While these indicators provide a robust measure of socioeconomic conditions and stability, we recognize they are relatively slow-moving. They were prioritized here to ensure data completeness across the longitudinal study period and to minimize missingness that often affects higher-frequency indicators in conflict-affected settings” (Lines 122-127). In the Discussion’s limitation section, we included the language, “Future research should leverage subnational and higher-frequency data—such as satellite-derived night-lights or service-delivery measures—to clarify these mechanisms and evaluate threshold effects across other vaccine-preventable diseases.” (Lines 493-495).**

7) Variables containing zeros were transformed using log(x+1) prior to standardisation. Adding 1 can distort the distribution when zeros represent true absence versus under-reporting. The authors should justify this choice and consider sensitivity analyses using alternative transformations (e.g., inverse hyperbolic sine). For measles in particular, zero values may reflect surveillance gaps rather than true zero incidence. **-> We thank the reviewer for this important point. In the main analyses, variables with zero values were transformed using log(x+1) to stabilize variance and reduce right skew while retaining observations with zero values, a common approach in cross-national epidemiologic analyses where zeros may reflect either true absence or measurement limitations. We agree, however, that adding a constant can differentially affect distributions when zeros arise from heterogeneous surveillance capacity rather than true zero incidence, particularly for measles outcomes. To address this concern, we conducted additional sensitivity analyses using inverse hyperbolic sine (IHS) transformations for all zero-inflated variables, including measles cases/incidence and battle-related deaths (S5 Table). The IHS transformation accommodates zero values without requiring an arbitrary constant and closely approximates the log transformation at higher values. Results from the IHS models closely mirrored the main findings: latent socioeconomic status loadings were unchanged, associations between socioeconomic status and measles outcomes remained strong and negative, and the direction, magnitude, and statistical significance of pathways involving conflict intensity and displacement were substantively consistent across specifications. Model fit indices were also nearly identical. These findings indicate that our conclusions are likely not sensitive to the choice of transformation for zero-inflated variables and appear to remain robust under an alternative approach that explicitly addresses the reviewer’s concern regarding true zeros versus under-reporting. We have added this sensitivity analysis and corresponding discussion to the Supplementary Materials and Methods section, respectively.**

- **We added the following text to the Methods section: “To assess sensitivity to the treatment of zero-valued observations, we conducted robustness analyses using inverse hyperbolic sine (IHS) transformations for all zero-inflated variables, including measles cases, measles incidence per million, and battle-related deaths. Unlike log(x+1), the IHS transformation accommodates zero values without adding an arbitrary constant and approximates the log transformation for larger values. All IHS-transformed variables were standardized prior to model estimation, and structural specifications were otherwise identical to the main analyses” (Lines 155-161).**
- **We also added the following text to the Results section under robustness analyses: “Results from models using IHS-transformed variables were highly consistent with the primary log(x+1) specifications (S5 Table). Estimated loadings for the latent socioeconomic status construct, as well as the direction and magnitude of associations linking socioeconomic status, conflict intensity, displacement, and measles outcomes, remained substantively unchanged, with nearly identical model fit indices. These findings indicate that the main conclusions are robust to alternative transformations that explicitly accommodate zero-valued observations” (Lines 394-398).**

8) Although FIML was used, more detail is needed on the extent and structure of missingness for each variable (beyond the heatmap in Figure S1), whether the Missing at Random (MAR) assumption is plausible, and whether alternative approaches (e.g., pattern-mixture models, multiple imputation) were considered. Given heterogeneous data quality across countries, the MAR assumption may not hold. **-> We agree that the validity of the Missing at Random (MAR) assumption is critical, especially in humanitarian research. We conducted additional tests to check whether the MAR assumption likely holds for the purposes of our study. We began by conducting diagnostic logistic regressions predicting missingness for displacement and development indicators, which revealed that while data gaps are significantly associated with conflict intensity (OR=1.19 per log-unit increase in BRDs), they were not significantly predicted by the outcome variable (measles cases) after conditioning on conflict. Because FIML provides unbiased parameter estimates when the variables that predict missingness are included in the structural model, our approach remains consistent under a MAR mechanism where missingness is ignorable conditional on observed conflict and socioeconomic indicators.**

- **To rigorously test the robustness of our findings against potential violations of the MAR assumption, we performed three additional sensitivity analyses (now summarized in Table S8): (1) Auxiliary Variable SEM: We incorporated population size and HDI as auxiliary variables to further inform the FIML likelihood and reduce potential non-reporting bias; (2) Data Quality Subsampling: We re-estimated the model restricted to a "high-quality" subsample of countries with ≥80% data completeness for conflict and displacement indicators; and (3) Multiple Imputation: We re-estimated the primary model using Multiple Imputation by Chained Equations (MICE) with Predictive Mean Matching (m=5). As shown in Table S8, the structural pathways remained stable in magnitude, direction, and significance across all four distinct frameworks. For instance, the association between the socioeconomic latent construct and measles incidence remained consistent (range: −0.34 to −0.36; p<0.001) whether using FIML, MICE, or complete-case subsampling. This high degree of convergence suggests that our findings are not artifacts of missing-data structures.**
- **We therefore added the following text to the Methods: “To ensure robustness, we cross-validated our results using auxiliary variable SEM, Multiple Imputation by Chained Equations (MICE), and subsample analyses restricted to countries with ≥80% data completeness (see S8 Table). Latent variables were scaled using standardized factor loadings, and estimation was permitted up to 500 iterations to ensure convergence. Prior to analysis, we assessed the pattern and extent of missingness across all variables included in the models, calculating the proportion of missing values per variable, overall, and stratified by year and country. As shown in the Supplementary Materials, we additionally visualized missingness patterns using heatmaps and UpSet plots to identify any co-occurring or systematic missing data. These steps ensured that missing values were not concentrated in specific years, countries, or variables, reducing the risk of bias in the SEM estimates.” (Lines 218-228).**

9) The manuscript uses UCDP battle-related death counts aggregated at country-year level. UCDP notes that these estimates may be incomplete for low-intensity conflicts or remote settings. The authors should discuss how such measurement error may bias coefficients toward the null or produce spurious lagged effects. **-> We appreciate this critical observation regarding the limitations of the UCDP GED dataset. We acknowledge that battle-related deaths are often subject to under-reporting, particularly in low-intensity conflicts or geographically remote areas where media and NGO access is restricted. As the reviewer notes, such measurement error is likely to result in attenuation bias (bias toward the null), potentially leading to an underestimate of the true association between conflict intensity and the health outcomes studied. Regarding spurious lagged effects, we agree that this would specifically require the temporal misclassification of events (i.e., deaths occurring in year t being recorded in year t+1). While UCDP employs a rigorous backdating methodology to minimize this, the “noise” introduced by delayed reporting cycles in conflict zones remains a possibility. We have updated the Limitations section to distinguish between these two risks: attenuation due to under-counting and potential temporal bias due to reporting lags as follows: “Further, conflict-driven surveillance collapse and restricted healthcare access systematically induce under-reporting, suggesting that our findings represent a conservative lower bound of the true public health burden. While UCDP BRDs are a gold-standard metric, measurement error in remote or low-intensity settings may further contribute to attenuation bias, also leading to conservative estimates... We also acknowledge the risk of spurious lagged effects; if the reporting of deaths is temporally displaced (e.g., due to delayed documentation in volatile regions), the observed timing of the socioeconomic and health impacts may be artificially shifted. These factors suggest that our findings should be interpreted with caution regarding the precise timing and total magnitude of conflict effects.” (Lines 463-467 and 474-478).**

10) The analytic model assumes a unidirectional pathway: conflict → displacement → socioeconomic development → measles. However, outbreaks themselves may influence displacement patterns or prompt changes in surveillance activities. The authors should either justify ignoring this bidirectionality or explore its implications. **-> We thank the reviewer for highlighting the possibility of bidirectional relationships. To evaluate this concern, we conducted additional robustness analyses allowing for lagged reverse pathways from measles incidence to subsequent displacement and conflict intensity. While lagged measles incidence was positively associated with later displacement and battle-related deaths, models specifying these feedback pathways exhibited very poor overall fit (CFI = 0.70; RMSEA = 0.33), suggesting that such bidirectional dynamics are not well supported by the data. We therefore estimated alternative specifications allowing contemporaneous correlated residuals between measles incidence and conflict/displacement, capturing shared shocks such as surveillance disruption or institutional collapse without imposing direct causal feedback. These models demonstrated excellent fit (CFI = 0.99; RMSEA = 0.06) and preserved the magnitude and direction of all primary pathways. Together, these results suggest that while measles outbreaks co-occur with conflict-related processes, the dominant structural pathway runs from conflict and displacement to socioeconomic deterioration and measles risk, rather than the reverse.**

- **We therefore added the following text to the Results section in the robustness analysis section, “Models testing reverse pathways (measles incidence to displacement and conflict) performed poorly, whereas specifications permitting correlated residuals fit the data well (S6 Table). This pattern suggests observed associations reflect shared institutional and surveillance dynamics rather than direct feedback from outbreaks to conflict processes.” (Lines 399-402).**
- **We also added the following caveat to the limitation section within the Discussion: “Although we focus on the pathway from conflict to health, reciprocal relationships remain possible, as large-scale outbreaks can exacerbate socioeconomic instability and fuel social grievances. Consequently, our findings should be interpreted as structural associations that align with existing humanitarian theory, rather than as definitive causal evidence of directionality.” (Lines 484-488).**

11) Several robustness models report RMSEA values >0.10 and TLI <0.90 (e.g., Supplementary S1, S2, and S3 Tables). The manuscript should comment on these poorer fits and appropriately qualify interpretation of those models. **-> We thank the reviewer for this observation. We acknowledge that while our primary models demonstrate good fit, certain robustness specifications—particularly those incorporating high-frequency measles lags (Table S2) or alternative IHME data (Table S3)—exhibit sub-optimal global fit indices (RMSEA>0.10 and TLI<0.90). As the reviewer suggests, we have updated the Results and Discussion to qualify these findings. We have clarified that these robustness checks were intended to assess the stability of direction and significance for our core paths (e.g., Conflict → Socioeconomic Development → Measles) rather than to propose a perfectly specified alternative model. In all specifications, despite variations in global fit, the core indirect pathways remained consistent. We have also noted that RMSEA is known to be sensitive to sample size and model complexity in longitudinal country-year data, which may contribute to these higher values.**

- **To that end, we added the following text to the Results: “While core path coefficients remained stable in direction and significance, global model fit was variable (CFI 0.902–0.988, TLI 0.804–0.968, RMSEA 0.082–0.224). Specifications incorporating IHME incidence data and auto-regressive lags (Tables S2 and S3) exhibited sub-optimal global fit (RMSEA > 0.10, TLI < 0.90), likely reflecting the complex covariance structures of these alternative datasets rather than a failure of the underlying structural logic.” (Lines 384-389).**
- **We also added the following text to the Discussion: “Finally, while FIML and Multiple Imputation yielded consistent structural associations, global fit indices in supplementary models (e.g., RMSEA) were sensitive to high-variance longitudinal data and lagged variables. This necessitates caution in interpreting absolute fit, though the stability of local path coefficients across all specifications strengthens our confidence in the core associations.” (Lines 489-493).**

12) With a 24-year panel, serial autocorrelation in predictors and outcomes is likely. Failure to diagnose and correct for this (e.g., via GLS, Newey-West, AR(1) structures) may bias results. SEM does not automatically adjust for autocorrelation unless explicitly specified. **-> We agree that serial autocorrelation is a concern in long panel data. In response, we conducted additional robustness analyses explicitly modeling AR(1) temporal dependence within the SEM framework using both structural lags and residual covariance between adjacent time points. We estimated two specifications: (1) a full AR(1) model allowing lagged dependence and residual correlation for conflict, displacement, and measles incidence; and (2) a more parsimonious model allowing AR(1) dependence for the outcome only. Across both specifications, all key structural relationships remain substantively unchanged in magnitude, direction, and statistical significance. In particular, conflict intensity and displacement continue to exert independent negative effects on socioeconomic development, and both socioeconomic conditions and conflict intensity remain significant predictors of measles incidence. Model fit statistics strongly favor the outcome-only AR(1) specification, suggesting that while temporal dependence is present—especially for conflict processes—over-parameterizing AR structures may degrade model performance. These findings indicate that our conclusions are not driven by unmodeled serial correlation and are robust to explicit correction for temporal dependence.**

- **We therefore added the S7 Table with these results and added the following text to the Results section: “Finally, accounting for AR(1) serial autocorrelation, either across all endogenous variables or for the outcome alone, did not materially alter the key structural pathways (S7 Table).” (Lines 414-416).**

Minor Comments
13) Clarify whether continuous variables were standardised within-country or globally, as this affects interpretability and comparability. **-> All continuous variables were standardized globally across the entire longitudinal dataset. This approach was chosen to preserve the relative differences in development and conflict intensity between countries, ensuring that the latent construct and path coefficients reflect cross-national variations in addition to temporal changes. We have added the following text to the Methods: “All continuous variables were then standardized globally across the full dataset (centered at the grand mean with a standard deviation of 1) rather than within-country.” (Lines 147-148).**

14) The SEM equations are not presented. Providing explicit structural equations would enhance transparency. **-> We agree that providing the formal mathematical specification of the model enhances transparency and reproducibility. We have added the structural equations to the "S1 Text" (Statistical Appendix). These equations explicitly define the measurement model for our latent socioeconomic construct and the system of simultaneous regression equations used to estimate the direct and indirect pathways between conflict, displacement, and measles outcomes. We also included the following line in the Methods “…and the SEM equations are captured in the S1 text.” (Line 192).**

15) Although low-reporting vaccines were excluded, the rationale for averaging across heterogeneous vaccine types should be explained more clearly. **-> We appreciate this request for clarification. We utilized the mean coverage of these ten vaccines as a composite proxy for overall health system strength and primary healthcare reach. Averaging across these heterogeneous vaccines provides a more stable indicator of a country's immunization infrastructure than any single vaccine could alone, as it accounts for varying challenges in the cold chain, rural outreach, and maternal-child health contact points. Furthermore, using a mean prevents issues of multicollinearity that would arise if multiple highly-correlated vaccines were included individually in the SEM. Importantly, to account for the specific biological relationship with our outcome, we also ran specifications using only MCV1 coverage to ensure that the broader "health system" proxy was not masking measles-specific immunity effects.**

- **We also added the following text to the Methods section: “The rationale for using a mean across ten vaccines was to capture a composite measure of health system reach and infrastructure stability, which may be more representative of general primary care resilience than any single indicator. To improve comparability across country-years, we excluded vaccines with substantial imbalance in reporting, retaining data on ten vaccines… While this vaccination mean serves as a broader structural proxy, we also utilized MCV1 coverage independently in separate model specifications to isolate the specific association of measles-containing vaccine coverage with measles incidence” (Lines 130-133 and 139-141).**

16) The justification for using tuberculosis incidence as a comparator outcome is limited. TB transmission dynamics differ markedly from measles; further explanation is warranted. **-> We agree that the transmission dynamics of tuberculosis (TB) and measles differ significantly. However, TB was selected not as a biological analog, but as a system-level comparator to evaluate the robustness of our structural model. While measles is a highly contagious, acute viral infection that serves as a “canary in the coal mine” for rapid health system disruption, TB is a chronic bacterial infection that reflects longer-term socioeconomic deterioration, overcrowding, and sustained breakdowns in diagnostic and treatment infrastructure. By testing the model against TB, we aimed to determine if the identified pathways, specifically the link between conflict-driven socioeconomic decline and infectious disease, held true for a pathogen with a vastly different epidemiological profile. This strengthens the argument that conflict acts as a general catalyst for infectious disease resurgence through structural degradation, rather than being a phenomenon unique to vaccine-preventable viral outbreaks. In reviewing our manuscript, we realized that we had not done an adequate job of communicating this rationale behind selecting TB. We therefore added the following text to the Methods section: “We additionally employed tuberculosis (TB) incidence (cases per 100,000 population) from the World Bank as a comparator outcome to assess model robustness (11). While measles reflects acute immunization gaps, TB serves as a proxy for the long-term structural and socioeconomic deterioration, such as overcrowding and malnutrition, inherent in conflict-affected settings.” (Lines 96-100).**

17) The very high temporal persistence of BRDs (β ≈ 0.90) suggests potential non-stationarity. The authors should consider differencing or detrending. **-> We thank the reviewer for this comment regarding temporal persistence and potential non-stationarity. To address this, we conducted a sensitivity analysis using first-differenced variables (ΔX=X[t] – X[t−1]), which focuses purely on year-over-year changes and eliminates stochastic trends. As shown in the new Table S10, the structural core of our model remains robust to this highly conservative transformation. Specifically, an escalation in displacement significantly predicts a contemporaneous decline in the socioeconomic latent construct (β=−0.37, p=0.012), even when removing all level-based information. While the path from socioeconomic change to measles incidence became non-significant (likely due to the high volatility and measurement error inherent in year-on-year measles reporting) the stability of the intermediate structural pathways suggests that our primary results are not artifacts of spurious non-stationary trends. This analysis strongly suggests that conflict-driven shocks in displacement directly drive socioeconomic deterioration. We therefore added the following lines to the Results section: “A first-difference SEM focusing on year-over-year changes (S10 Table) confirmed the stability of the model’s internal logic: escalated displacement was significantly associated with a contemporaneous decline in the socioeconomic latent construct (β=−0.37,p=0.012). While annual changes in measles incidence were more volatile, the persistence of the displacement-socioeconomic link suggests the model captures genuine structural dynamics rather than secular trends.”** **(Lines 402-407).**

18) Some standardised coefficients are unexpectedly large (e.g., socioeconomic development → TB incidence). The authors should discuss plausibility and potential unmeasured confounding. **-> We appreciate the reviewer’s attention to the magnitude of the structural coefficients in our comparator models. The large standardized coefficient between Socioeconomic Status (ηSE ) and TB incidence (β=−0.82) is epidemiologically plausible. Tuberculosis is uniquely sensitive to the structural determinants of health; its transmission and progression are fundamentally driven by overcrowding, malnutrition, and limited access to primary care, factors directly proxied by our latent construct’s indicators (GDP, life expectancy, and schooling). In global health literature, socioeconomic indices often explain a vast majority of the variance in TB incidence, unlike measles, which is more dependent on specific immunization program performance. We therefore added the following note to the S4 Table: “The large standardized coefficient between Socioeconomic Status and TB incidence (β=−0.82) reflects the high sensitivity of tuberculosis to structural determinants of health, including housing quality, nutritional status, and healthcare access. In contrast to measles, which is driven by discrete immunization events, TB is widely recognized as a "disease of poverty" with incidence rates that closely track national development indices. Furthermore, as a latent variable model, the structural paths in this SEM are corrected for measurement error in the indicators (GDP, life expectancy, schooling), which typically results in larger coefficients than observed in standard regression models. We acknowledge that this estimate may also encompass unmeasured confounding from factors highly correlated with development, such as HIV prevalence and urban population density.” (Lines 689-698).**

19) The Methods state that "robust SEs using MLE" were used, but it is unclear whether standard errors were clustered by country. **-> We thank the reviewer for this suggestion. We wish to clarify that our primary models utilized *lavaan’s* MLR estimator, which provides Huber-White "sandwich" standard errors. This approach is robust to violations of multivariate normality and heteroscedasticity, ensuring a high baseline of statistical rigor even before additional clustering is applied. Furthermore, the use of FIML helps ensure that the standard errors are estimated using all available data points, maintaining power and reducing bias under the MAR assumption. However, we recognize that in longitudinal panel data, clustering by the geographic unit (ISO3 code) provides an additional, more conservative layer of inference. To address this, we have re-estimated all models with cluster-robust standard errors (S9 Table). We find that our point estimates remain virtually identical, confirming the structural stability of the model. While the confidence intervals widen under this more conservative threshold (rendering some secondary contemporaneous paths marginal) the core structural pathways, particularly the link between socioeconomic degradation and measles incidence, remain highly significant (p<0.001). We have updated the manuscript to reflect these nuances.**

- **We added the following note to the Results section: “Our primary models utilized the MLR estimator with Huber-White sandwich standard errors, providing robustness against non-normality and heteroscedasticity. To further evaluate the impact of within-country temporal correlation, we conducted a sensitivity analysis using cluster-robust standard errors (Table S9). While this more conservative approach yielded wider confidence intervals, the point estimates remained stable. Notably, the association between the socioeconomic latent construct and measles incidence remained robust across all specifications (Model A β: −0.34 [95% CI: −0.43, −0.25], p<0.001).” (Lines 390-394).**

20) Path diagrams (Figures 1-2) could be improved in clarity, including label readability, arrow thickness, and clearer depiction of latent variables. **-> Thank you for this suggestion. We have revised Figures 1–2 to improve clarity and readability by increasing label size and arrow thickness. In addition, in Figure 1 we have more clearly indicated the latent socioeconomic status construct by explicitly labeling the observed indicators as components of the socioeconomic development latent variable. These changes are intended to make the model structure and interpretation more transparent.**


Reviewer #2: This study focuses on the association between armed conflict and measles burden. The topic is practically relevant to both public health and global development. The methodological design (fixed-effects panel regression + SEM) is well-suited for multi-pathway analysis of longitudinal panel data. However, improvements are needed regarding the causal inference and practical interpretation of effect sizes.

Limitations & Recommendations:
1.Despite the inclusion of lagged terms, the SEM framework still hinges on the a priori causal pathway assumption of conflict-displacement-socioeconomic development. While longitudinal panel SEM can account for time trends, it cannot fully mitigate confounding from bidirectional causality—for instance, measles outbreaks may further erode socioeconomic systems, which could in turn amplify conflict risk. This should be discussed in the limitations section. **-> We agree with the reviewer that, even with longitudinal panel SEM and lagged specifications, causal directionality cannot be definitively established and that residual bidirectional dynamics may persist. To assess this concern empirically, we estimated alternative models allowing lagged reverse pathways from measles incidence to subsequent displacement and conflict intensity (S6 Table). Although lagged measles incidence was positively associated with later displacement and conflict, these bidirectional specifications exhibited very poor overall fit (CFI = 0.70; RMSEA = 0.33), suggesting that such feedback pathways are not well supported by the data. We further estimated models allowing contemporaneous correlated residuals between measles incidence and conflict/displacement, capturing shared institutional or surveillance shocks without imposing direct causal feedback; these models demonstrated excellent fit (CFI = 0.99; RMSEA = 0.06) and preserved the magnitude and direction of all primary pathways (S6 Table).** **Together, these results suggest that while measles outbreaks co-occur with conflict-related processes, the dominant structural pathway runs from conflict and displacement to socioeconomic deterioration and measles risk, rather than the reverse. Nonetheless, we acknowledge that panel SEM cannot fully eliminate confounding from longer-term or unmeasured reciprocal processes (for example, indirect effects of outbreaks on institutional capacity or socioeconomic conditions that may reinforce conflict risk over extended horizons).**

- **We therefore added the following text to the Results section in the robustness analysis section, “Models testing reverse pathways (measles incidence to displacement and conflict) performed poorly, whereas specifications permitting correlated residuals fit the data well (S6 Table). This pattern suggests observed associations reflect shared institutional and surveillance dynamics rather than direct feedback from outbreaks to conflict processes.” (Lines 399-402).**
- **We also added the following caveat to the limitation section within the Discussion: “Although we focus on the pathway from conflict to health, reciprocal relationships remain possible, as large-scale outbreaks can exacerbate socioeconomic instability and fuel social grievances. Consequently, our findings should be interpreted as structural associations that align with existing humanitarian theory, rather than as definitive causal evidence of directionality.” (Lines 484-488).**

2. The study applies log-transformation followed by standardization to highly skewed variables (e.g., GDP, BRDs). Although log-transformation improves distribution, it compresses differences between extreme values (e.g., the gap between large-scale and small-scale conflict BRDs is reduced after log-transformation), potentially underestimating the impact of severe conflicts on measles. **-> We thank the reviewer for raising this important concern regarding the potential compression of extreme values following log-transformation and standardization, particularly for highly skewed variables such as GDP and battle-related deaths. In the primary analyses, log-transformation was used to stabilize variance and limit undue influence of extreme observations in a cross-national setting characterized by substantial heterogeneity in scale. We agree, however, that log-transformation can attenuate differences between extreme values and could, in principle, understate the influence of very high-intensity conflicts. To evaluate the sensitivity of our findings to this issue, we conducted additional robustness analyses using inverse hyperbolic sine (IHS) transformations for all highly skewed and zero-inflated variables, including GDP and battle-related deaths (S5 Table). Unlike log(x+1), the IHS transformation preserves relative differences at higher values while accommodating zero observations without the addition of an arbitrary constant. Results from the IHS-based models closely mirrored those from the primary specifications: the direction, magnitude, and statistical significance of pathways linking conflict intensity, displacement, socioeconomic status, and measles outcomes were substantively unchanged, and model fit indices were nearly identical. These findings suggest that the main conclusions are not driven by compression of extreme values under log-transformation and are robust to alternative transformations that better preserve variation at the upper tail. We have clarified this point in the Methods and robustness Results sections:**

- **We added the following text to the Methods section: “To assess sensitivity to the treatment of zero-valued observations, we conducted robustness analyses using inverse hyperbolic sine (IHS) transformations for all zero-inflated variables, including measles cases, measles incidence per million, and battle-related deaths. Unlike log(x+1), the IHS transformation accommodates zero values without adding an arbitrary constant and approximates the log transformation for larger values. All IHS-transformed variables were standardized prior to model estimation, and structural specifications were otherwise identical to the main analyses” (Lines 155-161).**
- **We also added the following text to the Results section under robustness analyses: “Results from models using IHS-transformed variables were highly consistent with the primary log(x+1) specifications (S5 Table). Estimated loadings for the latent socioeconomic status construct, as well as the direction and magnitude of associations linking socioeconomic status, conflict intensity, displacement, and measles outcomes, remained substantively unchanged, with nearly identical model fit indices. These findings indicate that the main conclusions are robust to alternative transformations that explicitly accommodate zero-valued observations” (Lines 394-398).**

3. The study only tests a 1-year lag term, but conflict impacts on measles may have longer lags (e.g., susceptible populations peak 2 years after vaccine coverage disruptions). It is recommended to add models with 2-year and 3-year lag terms to analyze the "temporal attenuation effect" of conflict impacts and clarify the duration of conflict's sustained influence on measles burden. **-> We have addressed the request for longer lags by estimating Models E and F (Table S11). To account for the potential multicollinearity introduced by including multiple highly correlated lags of battle deaths, we performed a Wald joint significance test for the battle death coefficients (t0 through t−3). In Model E (Measles Incidence), though the temporal persistence of conflict can inflate the standard errors of individual lags, the Wald test confirms that the 3-year history of battle deaths is jointly significant (χ2=17.03,df=4,p=0.0019). This provides robust evidence for the “temporal attenuation” effect: conflict has a sustained, multi-year impact on measles burden that, in this 3-year lag test, peaks at the 3-year mark. Interestingly, this joint significance was not observed in the rate-based Model F (p=0.298), suggesting that the structural association of conflict may be most clearly captured when examining the total volume of cases. These results confirm the sustained influence of conflict while justifying our use of the more parsimonious 0–1 year lag structure in the primary models to avoid the methodological risks of data thinning and multicollinearity. To capture the results of this new test, we added the following text to the results Section: “Extended lag analyses (S11 Table) indicated a sustained conflict association in Model AA (incidence counts), with a significant Wald joint significance test for Lags 0–3 (χ2=17.03,p=0.002). Although individual Lags 1 and 2 were non-significant, likely due to multicollinearity, a significant 3rd-year lag (β=0.09,p=0.003) may suggest a delayed peak in measles cases. Joint significance was not maintained in the rate-based Model BB (p=0.298), indicating sensitivity to outcome specification. Further, the 3-year lag structure required data truncation that may introduce survival bias.” (Lines 408-416). Overall, we greatly appreciate the reviewer’s comment, and believe this is an area for future research.**

4. Enhance the discriminability of latent variables: The study treats "socioeconomic development" as a single latent variable, but "GDP," "life expectancy," and "mean years of schooling" may correspond to distinct subdimensions (e.g., "economic level," "health level," "educational level"). It is recommended to test a second-order latent variable model: split these three indicators into two sub-latent variables ("economic development" and "human capital"), then aggregate them into "socioeconomic development" to more precisely capture the mediating effects of different dimensions on measles. **-> We thank the reviewer for the suggestion to enhance the discriminability of the socioeconomic dimensions. While we agree that these represent distinct subdimensions (Economic vs. Human Capital), to our knowledge, a formal second-order latent model is likely weakened and potentially even under-identified with only three indicators, as each sub-latent variable requires at least two unique indicators to be statistically stable. To address the reviewer’s intent, we performed a Multidimensional Mediation Analysis (S12 Table). In this model, we split the original latent construct into two parallel mediators: (1) Economic Level (observed GDP) and (2) Human Capital (a latent construct comprising life expectancy and schooling). This approach allows us to precisely capture the distinct mediating effects of each dimension. The results demonstrate that both dimensions have significant and nearly identical impacts on measles incidence (Standardized β=−0.18,p=0.038 for Economic Level; β=−0.18,p=0.050 for Human Capital). This suggests that while these subdimensions are conceptually distinct, they contribute equally to the structural environment that suppresses measles transmission. This analysis confirms the robustness of our primary model while providing the granular insight into mediating pathways requested by the reviewer.**

5. Clarify the hierarchical contribution of mediating pathways: The current results note that "the indirect effect is larger than the direct effect," but quantifying the contribution ratio of each pathway would strengthen interpretation: **-> We thank the reviewer for this suggestion. We agree that quantifying the relative weight of each pathway provides a clearer hierarchy of the mechanisms driving measles outbreaks. We have performed a formal effect decomposition for all four primary models (A–D). As shown in the new S13 Table, we find a distinct shift in hierarchy depending on the outcome metric. For total cases (Models A-B), the impact of battle-related deaths is primarily direct (73%), likely reflecting immediate shocks to health infrastructure and vaccination services. For incidence rates (Models C-D), the impact is predominantly mediated (up to 74%). Within these mediated pathways, the degradation of socioeconomic status, both directly and as a consequence of displacement (the "compound pathway"), represents the primary driver, accounting for over 50% of the total effect in Model C.**

- **We have updated the Results section and included S13 Table in the Supplementary Materials to provide these specific quantification ratios; we specifically added the following text to the Results section: “Decomposition of conflict effects (S13 Table) reveals that the mechanisms of impact differ by outcome metric. For measles incidence (Model C), indirect structural disruptions accounted for 73.9% of conflict’s total effect. This was primarily mediated by socioeconomic degradation (31.3%) and a sequential pathway where displacement-driven socioeconomic decline further exacerbated transmission (23.5%). Conversely, the influence on total case volume (Model A) was predominantly direct (72.9%). These findings suggest that while structural collapse drives sustained population-level incidence, active conflict serves as the primary catalyst for absolute case surges.” (Lines 354-361).**

6. In Table 2, the table notes should indicate the meaning of p-value symbols (e.g., *, **, ***). Additionally, it is not clear how Models 1-20 were constructed or how they differ from each other. For transparency and reproducibility, the manuscript should specify the dependent and independent variables included in each model—either in the table header, a footnote, or an appendix. A short description explaining the conceptual logic behind the model sequence (e.g., stepwise addition of predictors or theoretical blocks) would greatly improve readability. **-> We thank the reviewer for this constructive feedback on Table 2. We have revised the table notes to define all p-value symbols and have significantly expanded the description of our modeling strategy to ensure reproducibility.**

- **We added the following notes under the table: “All models include country and year fixed effects with robust standard errors clustered at the country level. Dependent Variables (DV): Models 1–5 (DV: GDP per Capita); Models 6–10 (DV: Population Displacement %); Models 11–14 (DV: Battle-related Deaths); Models 15–20 (DV: Measles Cases). Significance Levels: *p < 0.05, **p < 0.01, ***p < 0.001. Values in parentheses represent 95% Confidence Intervals. BRDs = Battle-related deaths (logged).” (Lines 288-291)**
- **We also added the following text to the Methods: “As detailed in Table 2, this sequence was structured according to a path-building logic designed to systematically isolate the individual drivers within the broader conflict-measles framework. Initial specifications (Models 1–10) evaluated the influence of contemporary and lagged conflict shocks on socioeconomic development and population displacement. Subsequent models (Models 11–14) examined the temporal auto-correlation of violence and the recursive associations between displacement and battle-related deaths. The final block (Models 15–20) estimated the total association between conflict and measles burden while systematically adjusting for socioeconomic and vaccination mediators to observe the attenuation of the direct conflict coefficient and identify the primary mechanisms of transmission.” (Lines 168-177).**

7.Table 3 contains numerous pathway coefficients but does not distinguish between "direct pathways / indirect pathways / total effects." It is recommended to add a column or labels: Insert a new "Path Type" column in Table 3, labeling each pathway as "Direct," "Indirect," or "Latent Loading." **-> We thank the reviewer for this suggestion. We have added the following path types to Table 3: latent loading, indirect (mediation), direct (autoregressive), and direct. We concur that this substantially strengthens how interpretable the table is.**


Reviewer #3: This is an interesting study of a very important and under-researched area, aiming to explore the linkage between armed conflict on measles outcomes worldwide through both direct and indirect pathways. The authors analyze data from 193 countries across 24 years, and apply a rigorous analytic approach to explore causality including a robust approach to data missingness.

Major comments
1.    Would be good to discuss unmeasured confounders that vary with time and that influence both conflict and health outcomes like measles and aren't addressed by the models: humanitarian access/activity, political instability etc. Conflict is often not an external force but emerges locally, and relationships between conflict/displacement/economics and public health are reciprocal and not only in a single causation pathway to health outcomes. **-> We thank the reviewer for this insightful comment. We agree that conflict is often endogenous to the socio-political systems we are studying and that unmeasured time-varying factors (particularly humanitarian access and political instability) play a critical role. We have revised the Discussion to explicitly acknowledge these complexities. Specifically, we now discuss how conflict often emerges from pre-existing institutional fragility that independently affects health, and how the relationship between outbreaks and instability may be reciprocal. We also highlight that the lack of data on humanitarian access remains a significant unmeasured confounder that could lead to an underestimation of the reported effects.**

- **We added the following sentences to our Discussion: “Third, endogeneity and unmeasured confounding remain inherent risks. Armed conflict is rarely a purely exogenous force; it often emerges from localized political instability that may independently degrade infrastructure prior to active fighting. Furthermore, our models do not account for time-varying factors such as humanitarian access or aid intensity. A contraction of humanitarian space may simultaneously increase outbreak risk and decrease surveillance reliability, further obscuring the true impact. Although we focus on the pathway from conflict to health, reciprocal relationships remain possible, as large-scale outbreaks can exacerbate socioeconomic instability and fuel social grievances. Consequently, our findings should be interpreted as structural associations that align with existing humanitarian theory, rather than as definitive causal evidence of directionality.” (Lines 479-488).**

2.    Is a 1-year lag the best approach, when often the factors in the model evolve over years, as does conflict. Also, a lot of the actual processes (both covariates and the conflict outcome) involved are sub-national and local, whereas the data are at the national level. Consider an expanded discussion of this in limitations. **-> We thank the reviewer for these important considerations regarding the temporal and spatial resolution of our data. Regarding the temporal structure, we agree that the health consequences of conflict are often protracted. As detailed in our response to Reviewer 2 and the new S11 Table, we conducted sensitivity analyses using 2-year and 3-year lags. These exploratory models suggest that the impact of conflict on measles case volume may peak at the 3rd year, likely reflecting the time required for a "susceptibility gap" to accumulate following vaccination disruptions.**

- **We have maintained the 1-year lag in our primary models to maximize sample size and avoid multicollinearity, but we have expanded our limitations to discuss these longer-term "temporal tails." We specifically note: “While 1-year lags were utilized for parsimony, exploratory analyses (Table S11) identified a "temporal tail" peaking at the three-year mark. This delay likely reflects the biological accumulation of an "immunity gap"—a compounding pool of susceptible children that eventually reaches a critical threshold for explosive outbreaks. The durable nature of vaccination disruptions and the logistical hurdles of post-conflict catch-up campaigns suggest that primary estimates may not capture the full, multi-year cumulative burden. We also acknowledge the risk of spurious lagged effects; if the reporting of deaths is temporally displaced (e.g., due to delayed documentation in volatile regions), the observed timing of the socioeconomic and health impacts may be artificially shifted. These factors suggest that our findings should be interpreted with caution regarding the precise timing and total magnitude of conflict effects.” (Lines 468-478).**
- **Regarding spatial scale, we acknowledge that national-level aggregation is a significant limitation. Conflict is inherently localized, and its impact on infrastructure or displacement may be devastating in specific districts while appearing negligible in national-level averages. We have expanded our Discussion to clarify that this spatial dilution likely biases our estimates toward the null, meaning our findings represent a conservative, lower-bound estimate of the true local impact, noting: “First, national-level aggregation introduces a risk of spatial dilution; by masking localized disruptions in conflict "hotspots," national averages likely bias our coefficients toward the null. Further, conflict-driven surveillance collapse and restricted healthcare access systematically induce under-reporting, suggesting that our findings represent a conservative lower bound of the true public health burden.” (Lines 461-467).**

  3.    The authors do acknowledge this ("measurement error and incomplete surveillance could bias associations to the null"), but it would be worth highlighting that conflict is itself a potential cause of under-reporting by disrupting surveillance systems, limiting healthcare access etc, which systematically could lead to significant under-estimation, even using a one-year lag. **-> We agree with the reviewer that conflict-driven disruption of surveillance systems introduces a systematic downward bias. We have revised the limitations section to clarify that conflict is not only a driver of disease but also a driver of "data invisibility." We added the language: “Further, conflict-driven surveillance collapse and restricted healthcare access systematically induce under-reporting, suggesting that our findings represent a conservative lower bound of the true public health burden. While UCDP BRDs are a gold-standard metric, measurement error in remote or low-intensity settings may further contribute to attenuation bias, also leading to conservative estimates.” (Lines 463-467).**

  4.    This study focused on the immediate and short-term impact of conflict, but conflict might have its greatest impact on measles incidence several years in the future. Conflict disrupts routine vaccination programs in a durable way because systems take time to rebuild, leading to accumulation of immunity gaps and a large pool of susceptible children that create conditions for outbreaks in the future. Catch up vaccination campaigns are challenging to implement in post-conflict conditions and immunity gaps may persist. Displacement usually continues long after conflict ends. The authors implicitly mention longer term impacts via weakened health systems and the delayed effects implied by the lagged variables, but it would be valuable to explicitly mention the mechanism leading to prolonged and compounding risk of measles over multiple years, which was not examined by this report. **-> We thank the reviewer for this insightful comment regarding the multi-year compounding of measles risk. We agree that the most profound health impacts of conflict may occur several years after the onset of violence due to the durable disruption of routine immunization. This creates an "immunity gap"—a compounding pool of susceptible children that eventually reaches a critical threshold for explosive outbreaks. We have revised our Discussion to explicitly describe this biological mechanism and have linked it to our exploratory findings of a peak impact at the three-year lag mark. We added the following text: “While 1-year lags were utilized for parsimony, exploratory analyses (Table S11) identified a "temporal tail" peaking at the three-year mark. This delay likely reflects the biological accumulation of an "immunity gap"—a compounding pool of susceptible children that eventually reaches a critical threshold for explosive outbreaks. The durable nature of vaccination disruptions and the logistical hurdles of post-conflict catch-up campaigns suggest that primary estimates may not capture the full, multi-year cumulative burden. We also acknowledge the risk of spurious lagged effects; if the reporting of deaths is temporally displaced (e.g., due to delayed documentation in volatile regions), the observed timing of the socioeconomic and health impacts may be artificially shifted. These factors suggest that our findings should be interpreted with caution regarding the precise timing and total magnitude of conflict effects.” (Lines 468-478).**

  5.    Are there other ways to measure/quantify armed conflict other than BRDs, and why was BRDs chosen as the best measure for this study? **-> We thank the reviewer for this question regarding our choice of conflict metric. While other measures exist, such as conflict event counts (frequency), duration (persistence), or binary indicators (occurrence), we utilized battle-related deaths (BRDs) from the UCDP as our primary predictor because it serves as the most reliable proxy for conflict intensity and severity. BRDs capture the lethal scale of violence, which is more directly linked to the sudden disruption of health infrastructure and the subsequent displacement of populations than event counts alone. Furthermore, BRDs provide a continuous, ratio-scale variable that is well-suited for structural equation modeling. We have added a brief justification for this choice to the Methods section. We added the text, “UCDP-derived BRDs were selected as our indicator of conflict intensity because it provides a standardized, continuous measure of severity; because of this, UCDP BRDs are often cited as one of the most reliable sources of conflict data (7).” (Lines 105-108).**

  6.    Generally, it would be good to reduce causal language given the limitations in assigning causality because of above comments) ("is associated with higher" instead of "increases") **-> We fully agree with the reviewer’s caution regarding causal language. We have conducted a comprehensive revision of the manuscript to replace causal terminology (including "increases," "leads to," and "impacts") with more appropriate associational language (e.g., "is associated with," "is linked to," "corresponds with"). We have also re-framed our structural equation model as an analysis of "structural associations" and theoretically grounded pathways rather than a definitive test of directionality. These changes are reflected throughout the Abstract, Methods, Results, and Discussion sections.**

  7.    It's not clear to me if "Displacement" refers to internal displacement, external displacement or if inflow of refugees is also included - please specify. **-> We thank the reviewer for identifying this need for clarification. Our measure of "Displacement" refers to the total number of individuals forcibly displaced from a given country, including both internally displaced persons (IDPs) and refugees/asylum seekers who have fled across international borders (external displacement). We focused on displacement by country of origin to capture the total population upheaval caused by localized conflict. Consequently, while it includes those displaced internally and those who have left the country, it does not include the inflow of refugees from other nations unless they were displaced from within the study country's own borders. We have updated the Methods section to explicitly define these components, noting: “For this analysis, exposure was defined as the annual country-level total of forcibly displaced persons by country of origin, aggregating internal displacement and external cross-border outflows due to incomplete disaggregated reporting across contexts. Population data were obtained from the World Bank to calculate the percentage of the population forcibly displaced (11).” (Lines 113-117).**
