# Supplementary material for: Association of armed conflict and global measles cases: A structural equation modeling analysis of 193 countries from 2000 to 2023
Source: PLoS Med. 2026 Jun 25;23(6):e1004819. doi: 10.1371/journal.pmed.1004819 (PMC13298743; doi:10.1371/journal.pmed.1004819)
Supplement: Attachment — Submitted filename: 20260419_PLOS Medicine_Response to Reviewers_Clean.docx [file pmed.1004819.s026.docx]

Reviewer #1: Overall, the authors have addressed my major methodological and statistical concerns in a thorough and constructive manner. In particular, they have clarified the rationale for using SEM as an exploratory, theory-informed framework; substantially strengthened the discussion of causal assumptions and limitations; implemented multiple robustness analyses (including AR(1) specifications, lag structures, first-difference models, alternative transformations, multiple imputation, and clustering adjustments); and appropriately qualified interpretation in light of model fit indices and ecological design constraints. 

I have one remaining minor clarification request. While vaccination coverage is incorporated as a mediator in several robustness models, the rationale for averaging across heterogeneous vaccine types could be explained more explicitly in the Methods section, including why this aggregation is appropriate for the conceptual framework and whether alternative specifications (e.g., measles-specific coverage only) were considered. This does not materially affect the main conclusions but would improve clarity. **-> We have clarified this rationale in the Methods section. For background, we intended for our vaccine composite to serve as a structural indicator of health service delivery. While we considered using MCV1-only coverage, we chose the aggregated measure because it is less sensitive to the noise of individual vaccine reporting; we therefore assessed that mean coverage would serve as a better indicator for our intended purpose. We amended our text to read: “We used this composite mean rather than measles-specific coverage (MCV1 only) to minimize the influence of reporting volatility. By averaging across multiple delivery platforms, including birth doses and multi-contact series, the index likely provides a more stable indicator of general primary care infrastructure and cold-chain stability in conflict-affected settings.” (lines 185-188)**


Reviewer #2: The manuscript has been substantially strengthened in revision, and I appreciate the authors' careful engagement with prior methodological concerns. I have only a few additional points for clarification that I believe would further enhance the rigor and interpretability of the study.

1.The analysis relies on annual panel data. Given that both conflict events and measles outbreaks may exhibit important short-term dynamics, it would be helpful if the authors could briefly discuss whether annual aggregation might obscure shorter-term conflict-disease interactions or introduce temporal aggregation bias. **-> We agree that annual aggregation is a limitation when studying a disease with rapid transmission dynamics like measles. We have expanded on the study limitations in Discussion: “Second, the structural impacts of conflict follow complex temporal cycles that may be obscured by annual aggregation. Our reliance on annual panel data likely introduces temporal aggregation bias by smoothing over acute, short-term interactions, such as monthly outbreak spikes or localized violence, which may attenuate our estimates toward the null. While this trade-off was necessary to maintain a global scope using standardized annual indicators, 1-year lags were utilized for parsimony, and exploratory analyses (Table S11) identified a "temporal tail" peaking at the three-year mark.” (Lines 547-553)**

2.The conflict, displacement, and measles datasets are likely derived from different reporting systems with potentially distinct time-stamping conventions. Could the authors clarify how temporal alignment across these sources was handled? A brief discussion of potential reporting lags, synchronization issues, or measurement timing discrepancies would be valuable, particularly given the lagged path structure in the SEM framework. **-> Thank you for this comment. We have added the following paragraph to the Methods section for clarification: “To ensure temporal alignment across disparate sources, all variables were aggregated using a standardized calendar-year window (January 1 to December 31). While reporting systems for conflict (UCDP), displacement (UNHCR), and immunization (WUENIC) operate under different institutional mandates, each provides validated annual estimates that correspond to the same temporal unit. We acknowledge that reporting lags exist, particularly in conflict zones where data may only be finalized 12–18 months post-event, but since our SEM framework utilizes lagged variables and finalized pre-2024 UCDP conflict data, these institutional reporting delays do not introduce synchronization bias into the model’s structural pathways.” (Lines 189-196).**

3.Structural equation models can sometimes be subject to model equivalence, where alternative path structures may fit the data comparably well. Beyond the reverse-path specification already examined, it would be useful if the authors could briefly comment on how they assessed the plausibility or relative uniqueness of their specified model structure. **-> We thank the reviewer for the opportunity to include a comment on how model structure was assessed. We added a paragraph to the Methods addressing model equivalence by grounding our structural specification in temporal precedence and theoretical primacy. By employing longitudinal panel data with lagged conflict variables, we attempted to establish a clear directional sequence that mitigates the risk of equivalent models often found in cross-sectional SEMs. Further, our SEM was not chosen via post-hoc fit-index optimization; rather, it was informed by a series of preliminary fixed-effects regressions that validated each specific path. To that end, we added the following text: “To address potential model equivalence where alternative path structures might yield identical fit indices, we prioritized a theory-driven approach to identifying pathways. The structural specification was anchored by the temporal precedence of conflict (using lagged BRDs) and informed by our preliminary fixed-effects regressions, which validated the directional associations between conflict, displacement, and socioeconomic indicators prior to SEM estimation. This ensures the model represents a plausible causal chain supported by both longitudinal data and humanitarian theory, rather than a purely data-driven configuration.” (Lines 247-253)**


Reviewer #3: This is an interesting study that tries to understand whether and how armed conflict contributes to measles burden worldwide using 23 years of country data and an SEM framework. It is an interesting and plausible premise, and the authors appropriately frame it as an exploratory or structural study and have appropriate caveats about causal inference. Some minor clarifications and corrections would be needed to prepare it for publication. The revision addresses the reviewer comments well

Specific comments and suggestions:

1) The abstract references displacement as the pathway to measles without clarifying it is mostly through its impact on socioeconomic development - could be rephrased to avoid reader confusion about the causative pathway **->** **Thank you for chance to make this change and improve the clarity of our abstract. We modified the abstract to read: “…and each SD increase in displaced population corresponded with a 0.20 SD decline in socioeconomic development (95% CI [−0.23, −0.17]; p < 0.001), which was the primary pathway by which displacement was associated with measles.” (Lines 40-43)**

2) In Table 1, measles cases are attributed to World Bank as the source, but the methods reference WHO as the source (more likely and appropriate). Please clarify **-> Thank you for this catch! Table 1 incorrectly specified that the information was from the World Bank; we have changed this to clarify that the data is from the World Health Organization, as stated in the Methods. (Lines 332-333)**

3) Figure 2 is not legible at all due to low resolution. **-> Our apologies; it looks like there was an issue with how Figure 2 rendered in the reviewer copy. We have attached a copy below in the original definition.**

4) Consider changing the title from "The effect.." to "the association" to be more consistent with the appropriately non-causal framing of the findings. **-> We have changed the title to “Association of armed conflict and global measles cases: a structural equation modeling analysis of 193 countries from 2000 to 2023”**
