# Supplementary material for: Association of armed conflict and global measles cases: A structural equation modeling analysis of 193 countries from 2000 to 2023
Source: PLoS Med. 2026 Jun 25;23(6):e1004819. doi: 10.1371/journal.pmed.1004819 (PMC13298743; doi:10.1371/journal.pmed.1004819)
Supplement: Attachment — Submitted filename: 20260530_Response to Editor_Final.docx [file pmed.1004819.s027.docx]

GENERAL

* Please disclose any use of AI tools and technologies to the study or article content in the Methods, including the name(s) of any tool(s) used and a description of their use. Please see our guidelines for more information: <https://urldefense.proofpoint.com/v2/url?u=https-3A__journals.plos.org_plosmedicine_s_ethical-2Dpublishing-2Dpractice-23loc-2Dartificial-2Dintelligence-2Dtools-2Dand-2Dtechnologies&d=DwIBaQ&c=slrrB7dE8n7gBJbeO0g-IQ&r=XGzUN6S7CV-sOjf7f4TPQQ&m=HS6wnEOVBprgCfBUHOMsfhvepu7R8FqbshmTEZIcsMQno58ccralfYCFhKwkZR_y&s=j99jmFc8wFMexlWXfySAe-o0AKtBQ7qPFitvi6VB2II&e=> .

**->** **We added a “Use of AI tools” section to the methodology, specifying that “We used Claude 4.5 to assist with generating R code to visualize results. The tool was not used to generate analytic decisions, interpret results, or draft scientific content. All AI-assisted output was reviewed and verified by the authors against primary sources and analytic outputs; the authors take full responsibility for the final manuscript.” (lines 350-354)**

* Please review your text for claims of novelty or primacy (e.g. 'novel') and remove this language. **-> We made edits throughout the paper to remove any claims of novelty or primacy.**

CODE AVAILABILITY

* Please ensure that your code is shared in a way that follows best practice and facilitates reproducibility and reuse. Because Github depositions can be readily changed or deleted, we request that you to make a permanent DOI'd copy (e.g. in Zenodo) and provide the URL in the Data Availability statement. **-> We added our code to Zenodo and included a new data availability section in the Methods: “All R code used to clean the data, fit the fixed-effects panel regressions, and estimate the structural equation models is permanently archived in Zenodo (DOI: 10.5281/zenodo.20299687). All underlying data are publicly available from the original sources cited in the Methods.” (Lines 356-359)**

FIGURES

* When a p value is given, please specify the statistical test used to determine it in the legend. **-> We have specified in the Table notes (Table 2 and 3) that the p-values are derived from two-sided Wald tests (Lines 407-408 and 477-478).**

* Please provide the unadjusted comparisons as well as the adjusted comparisons in all relevant Tables **-> We have added this language to the notes in Tables 2 and 3 (Lines 403-405 and 479-481) and specified that: “The first model in each block (Models 1, 6, 11, and 15) presents the unadjusted bivariate association between the primary exposure and outcome; subsequent models within each block progressively add covariates as listed in the column headers and are therefore adjusted estimates. Adjustment variables across the full table comprise: country fixed effects, year fixed effects, contemporaneous and one-year-lagged battle-related deaths (logged, standardized), population displacement (% of population, standardized), GDP per capita (logged, standardized), and mean vaccination coverage across ten vaccines (standardized).”**

* Please specify the variables controlled for in all relevant Tables **-> We have added this language to the notes in Tables 2 and 3 (Lines 403-408 and 472-477).**

ACKNOWLEDGMENTS

* Please include an Acknowledgments section in your manuscript. -> **We have done so: “The authors gratefully acknowledge the data custodians at the World Health Organization, the Uppsala Conflict Data Program, the United Nations High Commissioner for Refugees, the World Bank, the United Nations Development Programme, and the Institute for Health Metrics and Evaluation for making these data publicly available. The views expressed are those of the authors and do not necessarily represent those of the data sources.” (Lines 646-651)**

OBSERVATIONAL, COHORT, CROSS-SECTIONAL, AND CASE CONTROL STUDIES

*Please ensure that the study is reported according to the STROBE guideline, and include the completed STROBE checklist as Supporting Information.  Please add the following statement, or similar, to the Methods: ""This study is reported as per the Strengthening the Reporting of Observational Studies in Epidemiology (STROBE) guideline (S1 Checklist)."" **-> We have added the requested language and completed a STROBE checklist, attached to this resubmission, “This study is reported as per the Strenghtening the Reporting of Observational Studies in Epidemiology (STROBE) guidelines (S1 Checklist)” (Lines 131-132)**

The STROBE guideline can be found here: <https://urldefense.proofpoint.com/v2/url?u=http-3A__www.equator-2Dnetwork.org_reporting-2Dguidelines_strobe_&d=DwIBaQ&c=slrrB7dE8n7gBJbeO0g-IQ&r=XGzUN6S7CV-sOjf7f4TPQQ&m=HS6wnEOVBprgCfBUHOMsfhvepu7R8FqbshmTEZIcsMQno58ccralfYCFhKwkZR_y&s=mYz-1hA9ZDW0aNJYN3xZ0Z1hJV06gVwD9uoT9hNSJ1A&e=>

When completing the checklist, please use section and paragraph numbers, rather than page numbers.

* Did your study have a prospective protocol or analysis plan? Please state this (either way) early in the Methods section. **-> We have added the following text: “The hypotheses, primary structural equation models (Models A–D), and fixed-effects regression strategy were specified before data analysis began as part of the project's conceptual development, although no formal prospective protocol document was registered. The robustness analyses presented in the Supporting Information were planned in part prospectively and in part in response to peer-review comments. Analyses added in response to peer review, and therefore identified as data-driven, comprise: the inverse hyperbolic sine transformations (S5 Table); the first-order AR(1) specifications (S7 Table); the cluster-robust standard errors (S9 Table); the first-difference SEM (S10 Table); the extended distributed-lag analysis (S11 Table); the parallel mediation analysis (S12 Table); and the hierarchical decomposition of total effects (S13 Table). These additions did not alter the substantive conclusions of the primary analyses.” (Lines 132-142).**

a) If a prospective analysis plan (from your funding proposal, IRB or other ethics committee submission, study protocol, or other planning document written before analyzing the data) was used in designing the study, please include the relevant prospectively written document with your revised manuscript as a Supporting Information file to be published alongside your study, and cite it in the Methods section. A legend for this file should be included at the end of your manuscript. 

b) If no such document exists, please make sure that the Methods section transparently describes when analyses were planned, and when/why any data-driven changes to analyses took place.  

c) In either case, changes in the analysis-- including those made in response to peer review comments-- should be identified as such in the Methods section of the paper, with rationale.

* Your study is observational and therefore causality cannot be inferred. Please remove language that implies causality and refer to associations instead. **-> We have attempted to remove all language that implies causality (e.g., “effect”, “impact”, “causally”).**

* For all observational studies, in the manuscript text, please indicate: (1) the specific hypotheses you intended to test, (2) the analytical methods by which you planned to test them, (3) the analyses you actually performed, and (4) when reported analyses differ from those that were planned, transparent explanations for differences that affect the reliability of the study's results. If a reported analysis was performed based on an interesting but unanticipated pattern in the data, please be clear that the analysis was data-driven. **-> We have added the following text to the Methods in a new “Hypotheses and analytical plan” section: “We tested three a priori hypotheses: (H1) higher annual battle-related deaths within a country are associated with greater contemporaneous measles burden (cases and incidence per million population), adjusting for population displacement and socioeconomic development; (H2) higher one-year-lagged battle-related deaths are associated with a greater contemporaneous measles burden, capturing delayed disruption of health and immunization systems; and (H3) the association between armed conflict and measles burden is partly mediated by indirect pathways operating through population displacement and a latent construct of socioeconomic development. The prespecified analytical strategy to test these hypotheses comprised (i) descriptive summaries of all variables; (ii) fixed-effects panel regressions (Models 1-20, Table 2) to characterize the bivariate and progressively adjusted associations between conflict, displacement, socioeconomic indicators, and measles cases; and (iii) four prespecified structural equation models (Models A-D, Table 3, and Fig 2) to estimate direct and indirect path coefficients within a single coherent system. The analyses performed correspond to this plan with the additions noted above (S5, S9, S10, S11, S12, and S13 Tables) introduced as supplementary robustness analyses in response to peer-review comments. Where reported analyses differ from the prespecified plan, this is identified in the relevant subsection of the Results, Robustness analysis subsection, or footnotes to the corresponding table or figure.” (Lines 144-161).**
