# Supplementary material for: Association of armed conflict and global measles cases: A structural equation modeling analysis of 193 countries from 2000 to 2023
Source: PLoS Med. 2026 Jun 25;23(6):e1004819. doi: 10.1371/journal.pmed.1004819 (PMC13298743; doi:10.1371/journal.pmed.1004819)
Supplement: Peer review history

# Editor report: Decision Letter 0

**DOI:** 10.1371/journal.pmed.1004819.r001

**Authors:** Andreia Cunha

5 Nov 2025

Dear Dr Tozan,

Thank you for submitting your manuscript entitled "The Effect of Armed Conflict on Global Measles Cases: A Structural Equation Modeling Analysis of 193 Countries from 2000 to 2023" for consideration by PLOS Medicine.

Your manuscript has now been evaluated by the PLOS Medicine editorial staff, and I am writing to let you know that we would like to send your submission out for external peer review.

However, before we can send your manuscript to reviewers, we need you to complete your submission by providing the metadata that is required for full assessment. To this end, please login to Editorial Manager where you will find the paper in the 'Submissions Needing Revisions' folder on your homepage. Please click 'Revise Submission' from the Action Links and complete all additional questions in the submission questionnaire.

For clinical studies, please upload a copy of your trial study protocol as a supporting information file. The study protocol should be the version submitted for approval to the institutional review board or ethics committee, should include any amendments to the study protocol, as well as the date of their approval by the institutional review or ethics committee. Please also detail any deviations from the study protocol in the Methods section of your manuscript. The editors will consider the protocol and study conduct prior to a final decision for external review.

Please re-submit your manuscript within two working days, i.e. by Nov 07 2025 11:59PM.

Login to Editorial Manager here: https://www.editorialmanager.com/pmedicine

Once your full submission is complete, your paper will undergo a series of checks in preparation for peer review. Once your manuscript has passed all checks it will be sent out for review.

Feel free to email us at plosmedicine@plos.org if you have any queries relating to your submission.

Kind regards,

Andreia Cunha, PhD

Senior Editor

PLOS Medicine

# Peer review: Decision Letter 1

**DOI:** 10.1371/journal.pmed.1004819.r002

**Authors:** Andreia Cunha, Andreia Cunha

19 Dec 2025

Dear Dr Tozan,

Apologies for the delay in getting back to you with a decision, which was due to challenges in securing all the necessary advice.

Many thanks for submitting your manuscript "The Effect of Armed Conflict on Global Measles Cases: A Structural Equation Modeling Analysis of 193 Countries from 2000 to 2023" (PMEDICINE-D-25-03848R1) to PLOS Medicine. The paper has been reviewed by subject experts and a statistician; their comments are included below and can also be accessed here: [LINK]

As you will see, the reviewers find your work very interesting, but they raise several questions and concerns that we must ask you to address in full. After discussing the paper with the editorial team, I'm pleased to invite you to revise the paper in response to the reviewers' comments. We plan to send the revised paper to some or all of the original reviewers, and we cannot provide any guarantees at this stage regarding publication.

In addition to these revisions, you may need to complete some formatting changes, which you will receive in a follow up email. A member of our team will be in touch with a set of requests shortly. If you do not receive a separate email within a few days, please assume that checks have been completed, and no additional changes are required.

When you upload your revision, please include a point-by-point response that addresses all of the reviewer and editorial points, indicating the changes made in the manuscript and either an excerpt of the revised text or the location (eg: page and line number) where each change can be found. Please also be sure to check the general editorial comments at the end of this letter and include these in your point-by-point response. When you resubmit your paper, please include a clean version of the paper as the main article file and a version with changes tracked as a marked-up manuscript. It may also be helpful to check the guidelines for revised papers at http://journals.plos.org/plosmedicine/s/revising-your-manuscript for any that apply to your paper.

We ask that you submit your revision by Jan 09 2026 11:59PM. However, if this deadline is not feasible, please contact me by email, and we can discuss a suitable alternative.

Don't hesitate to contact me directly with any questions (acunha@plos.org).

Best regards,

Andreia

Andreia Cunha, PhD

Senior editor

PLOS Medicine

acunha@plos.org

-----------------------------------------------------------

Comments from the reviewers:

Reviewer #1: Major Comments

1) The authors should clarify why SEM was preferred over more robust causal inference methods (e.g., marginal structural models, difference-in-differences with staggered adoption, dynamic panel models), given the potential for time-varying confounding and reciprocal causation.

2) The manuscript states that fixed-effects regression was used to "inform SEM specification" and that SEM is used for "exploratory analysis," but the rationale for combining these two frameworks is not clearly articulated.

3) Although the authors include lagged BRDs to address simultaneity, the approach does not adequately resolve issues of temporal ordering or unmeasured confounding. SEM with contemporaneous and one-year lagged variables cannot establish causal direction in a setting with complex time-varying processes such as conflict, displacement, and health system deterioration.

4) The manuscript should clearly state the assumptions required for causal interpretation and explicitly discuss potential violations, including measurement error in BRDs and measles data, and omitted time-varying confounders (e.g., vaccination campaigns, political instability, drought/famine).

5) The analysis relies entirely on annual national aggregates, introducing substantial risk of ecological bias. Measles transmission dynamics and displacement events occur at far more granular temporal and spatial scales. The Methods should acknowledge that country-year averages may mask significant heterogeneity, and results should not be interpreted as individual-level or subnational causal effects.

6 Although the latent construct loads strongly on GDP per capita, schooling, and life expectancy, these are slow-moving structural variables that may not capture short-term socioeconomic disruptions resulting from conflict. The authors should discuss whether faster-moving indicators (e.g., health expenditure, poverty headcount, night-lights data, service-delivery measures) would better reflect conflict-related socioeconomic decline.

7) Variables containing zeros were transformed using log(x+1) prior to standardisation. Adding 1 can distort the distribution when zeros represent true absence versus under-reporting. The authors should justify this choice and consider sensitivity analyses using alternative transformations (e.g., inverse hyperbolic sine). For measles in particular, zero values may reflect surveillance gaps rather than true zero incidence.

8) Although FIML was used, more detail is needed on the extent and structure of missingness for each variable (beyond the heatmap in Figure S1), whether the Missing at Random (MAR) assumption is plausible, and whether alternative approaches (e.g., pattern-mixture models, multiple imputation) were considered. Given heterogeneous data quality across countries, the MAR assumption may not hold.

9) The manuscript uses UCDP battle-related death counts aggregated at country-year level. UCDP notes that these estimates may be incomplete for low-intensity conflicts or remote settings. The authors should discuss how such measurement error may bias coefficients toward the null or produce spurious lagged effects.

10) The analytic model assumes a unidirectional pathway: conflict → displacement → socioeconomic development → measles. However, outbreaks themselves may influence displacement patterns or prompt changes in surveillance activities. The authors should either justify ignoring this bidirectionality or explore its implications.

11) Several robustness models report RMSEA values >0.10 and TLI <0.90 (e.g., Supplementary S1, S2, and S3 Tables). The manuscript should comment on these poorer fits and appropriately qualify interpretation of those models.

12) With a 24-year panel, serial autocorrelation in predictors and outcomes is likely. Failure to diagnose and correct for this (e.g., via GLS, Newey-West, AR(1) structures) may bias results. SEM does not automatically adjust for autocorrelation unless explicitly specified.

Minor Comments

13) Clarify whether continuous variables were standardised within-country or globally, as this affects interpretability and comparability.

14) The SEM equations are not presented. Providing explicit structural equations would enhance transparency.

15) Although low-reporting vaccines were excluded, the rationale for averaging across heterogeneous vaccine types should be explained more clearly.

16) The justification for using tuberculosis incidence as a comparator outcome is limited. TB transmission dynamics differ markedly from measles; further explanation is warranted.

17) The very high temporal persistence of BRDs (β ≈ 0.90) suggests potential non-stationarity. The authors should consider differencing or detrending.

18) Some standardised coefficients are unexpectedly large (e.g., socioeconomic development → TB incidence). The authors should discuss plausibility and potential unmeasured confounding.

19) The Methods state that "robust SEs using MLE" were used, but it is unclear whether standard errors were clustered by country.

20) Path diagrams (Figures 1-2) could be improved in clarity, including label readability, arrow thickness, and clearer depiction of latent variables.

Reviewer #2: This study focuses on the association between armed conflict and measles burden. The topic is practically relevant to both public health and global development. The methodological design (fixed-effects panel regression + SEM) is well-suited for multi-pathway analysis of longitudinal panel data. However, improvements are needed regarding the causal inference and practical interpretation of effect sizes.

Limitations & Recommendations:

1.Despite the inclusion of lagged terms, the SEM framework still hinges on the a priori causal pathway assumption of conflict-displacement-socioeconomic development. While longitudinal panel SEM can account for time trends, it cannot fully mitigate confounding from bidirectional causality—for instance, measles outbreaks may further erode socioeconomic systems, which could in turn amplify conflict risk. This should be discussed in the limitations section.

2. The study applies log-transformation followed by standardization to highly skewed variables (e.g., GDP, BRDs). Although log-transformation improves distribution, it compresses differences between extreme values (e.g., the gap between large-scale and small-scale conflict BRDs is reduced after log-transformation), potentially underestimating the impact of severe conflicts on measles.

3. The study only tests a 1-year lag term, but conflict impacts on measles may have longer lags (e.g., susceptible populations peak 2 years after vaccine coverage disruptions). It is recommended to add models with 2-year and 3-year lag terms to analyze the "temporal attenuation effect" of conflict impacts and clarify the duration of conflict's sustained influence on measles burden.

4. Enhance the discriminability of latent variables: The study treats "socioeconomic development" as a single latent variable, but "GDP," "life expectancy," and "mean years of schooling" may correspond to distinct subdimensions (e.g., "economic level," "health level," "educational level"). It is recommended to test a second-order latent variable model: split these three indicators into two sub-latent variables ("economic development" and "human capital"), then aggregate them into "socioeconomic development" to more precisely capture the mediating effects of different dimensions on measles.

5. Clarify the hierarchical contribution of mediating pathways: The current results note that "the indirect effect is larger than the direct effect," but quantifying the contribution ratio of each pathway would strengthen interpretation:

6. In Table 2, the table notes should indicate the meaning of p-value symbols (e.g., *, **, ***). Additionally, it is not clear how Models 1-20 were constructed or how they differ from each other. For transparency and reproducibility, the manuscript should specify the dependent and independent variables included in each model—either in the table header, a footnote, or an appendix. A short description explaining the conceptual logic behind the model sequence (e.g., stepwise addition of predictors or theoretical blocks) would greatly improve readability.

7.Table 3 contains numerous pathway coefficients but does not distinguish between "direct pathways / indirect pathways / total effects." It is recommended to add a column or labels:

Insert a new "Path Type" column in Table 3, labeling each pathway as "Direct," "Indirect," or "Latent Loading."

Reviewer #3: This is an interesting study of a very important and under-researched area, aiming to explore the linkage between armed conflict on measles outcomes worldwide through both direct and indirect pathways. The authors analyze data from 193 countries across 24 years, and apply a rigorous analytic approach to explore causality including a robust approach to data missingness.

Major comments

1.Would be good to discuss unmeasured confounders that vary with time and that influence both conflict and health outcomes like measles and aren't addressed by the models: humanitarian access/activity, political instability etc. Conflict is often not an external force but emerges locally, and relationships between conflict/displacement/economics and public health are reciprocal and not only in a single causation pathway to health outcomes.

2.Is a 1-year lag the best approach, when often the factors in the model evolve over years, as does conflict. Also, a lot of the actual processes (both covariates and the conflict outcome) involved are sub-national and local, whereas the data are at the national level. Consider an expanded discussion of this in limitations.

3.The authors do acknowledge this ("measurement error and incomplete surveillance could bias associations to the null"), but it would be worth highlighting that conflict is itself a potential cause of under-reporting by disrupting surveillance systems, limiting healthcare access etc, which systematically could lead to significant under-estimation, even using a one-year lag.

4.This study focused on the immediate and short-term impact of conflict, but it conflict might have its greatest impact on measles incidence several years in the future. Conflict disrupts routine vaccination programs in a durable way because systems take time to rebuild, leading to accumulation of immunity gaps and a large pool of susceptible children that create conditions for outbreaks in the future. Catch up vaccination campaigns are challenging to implement in post-conflict conditions and immunity gaps may persist. Displacement usually continues long after conflict ends. The authors implicitly mention longer term impacts via weakened health systems and the delayed effects implied by the lagged variables, but it would be valuable to explicitly mention the mechanism leading to prolonged and compounding risk of measles over multiple years, which was not examined by this report.

5.Are there other ways to measure/quantify armed conflict other than BRDs, and why was BRDs chosen as the best measure for this study?

6.Generally, it would be good to reduce causal language given the limitations in assigning causality because of above comments) ("is associated with higher" instead of "increases")

7.It's not clear to me if "Displacement" refers to internal displacement, external displacement or if inflow of refugees is also included - please specify.

Any attachments provided with reviews can be seen via the following link: [LINK]

--------------------------------------------------------- ---

General editorial requests:

(Note: not all will apply to your paper, but please check each item carefully)

*We ask every co-author listed on the manuscript to fill in a contributing author statement, making sure to declare all competing interests. If any of the co-authors have not filled in the statement, we will remind them to do so when the paper is revised. If all statements are not completed in a timely fashion this could hold up the re-review process. If new competing interests are declared later in the revision process, this may also hold up the submission. Should there be a problem getting one of your co-authors to fill in a statement we will be in contact. Please do not add or remove authors without first discussing this with the handling editor. You can see our competing interests policy here: http://journals.plos.org/plosmedicine/s/competing-interests.

*Please upload any figures associated with your paper as individual TIF or EPS files with 300dpi resolution at resubmission; please read our figure guidelines for more information on our requirements: http://journals.plos.org/plosmedicine/s/figures. While revising your submission, we strongly recommend that you use PLOS's NAAS tool (https://ngplosjournals.pagemajik.ai/artanalysis) to test your figure files. NAAS can convert your figure files to the TIFF file type and meet basic requirements (such as print size, resolution), or provide you with a report on issues that do not meet our requirements and that NAAS cannot fix.

After uploading your figures to PLOS's NAAS tool - https://ngplosjournals.pagemajik.ai/artanalysis, NAAS will process the files provided and display the results in the "Uploaded Files" section of the page as the processing is complete.

If the uploaded figures meet our requirements (or NAAS is able to fix the files to meet our requirements), the figure will be marked as "fixed" above. If NAAS is unable to fix the files, a red "failed" label will appear above.

When NAAS has confirmed that the figure files meet our requirements, please download the file via the download option, and include these NAAS processed figure files when submitting your revised manuscript.

*Please ensure that the paper adheres to the PLOS Data Availability Policy (see http://journals.plos.org/plosmedicine/s/data-availability), which requires that all data underlying the study's findings be provided in a repository or as Supporting Information. For data residing with a third party, authors are required to provide instructions with contact information (web or email address) for obtaining the data. Please note that a study author cannot be the contact person for the data. PLOS journals do not allow statements supported by "data not shown" or "unpublished results." For such statements, authors must provide supporting data or cite public sources that include it.

*We expect all researchers with submissions to PLOS in which author-generated code underpins the findings in the manuscript to make all author-generated code available without restrictions upon publication of the work. In cases where code is central to the manuscript, we may require the code to be made available as a condition of publication. Authors are responsible for ensuring that the code is reusable and well documented. Please make any custom code available, either as part of your data deposition or as a supplementary file. Please add a sentence to your data availability statement regarding any code used in the study, e.g. "The code used in the analysis is available from Github [URL] and archived in Zenodo [DOI link]" Please review our guidelines at https://journals.plos.org/plosmedicine/s/materials-software-and-code-sharing and ensure that your code is shared in a way that follows best practice and facilitates reproducibility and reuse. Because Github depositions can be readily changed or deleted, we encourage you to make a permanent DOI'd copy (e.g. in Zenodo) and provide the URL.

FORMATTING - GENERAL

*Abstract: Please structure your abstract using the PLOS Medicine headings (Background, Methods and Findings, Conclusions). Please combine the Methods and Findings sections into one section.

*At this stage, we ask that you include a short, non-technical Author Summary of your research to make findings accessible to a wide audience that includes both scientists and non-scientists. The Author Summary should immediately follow the Abstract in your revised manuscript. This text is subject to editorial change and should be distinct from the scientific abstract. Ideally each sub-heading should contain 2-3 single sentence, concise bullet points containing the most salient points from your study. In the final bullet point of 'What Do These Findings Mean?', please include the main limitations of the study in non-technical language. Please see our author guidelines for more information: https://journals.plos.org/plosmedicine/s/revising-your-manuscript#loc-author-summary.

*Please express the main results with 95% CIs as well as p values. When reporting p values please report as p<0.001 and where higher as the exact p value p=0.002, for example. Throughout, suggest reporting statistical information as follows to improve clarity for the reader "22% (95% CI [13%,28%]; p</=)". Please be sure to define all numerical values at first use.

*Please include page numbers and line numbers in the manuscript file. Use continuous line numbers (do not restart the numbering on each page).

*Please cite the reference numbers in square brackets. Citations should precede punctuation.

FIGURES AND TABLES

*Please provide titles and legends for all figures and tables (including those in Supporting Information files).

*Please define all abbreviations used in each figure/table (including those in Supporting Information files).

*Please consider avoiding the use of red and green in order to make your figure more accessible to those with color blindness.

SUPPLEMENTARY MATERIAL

*Please note that supplementary material will be posted as supplied by the authors. Therefore, please amend it according to the relevant comments outlined here.

*Please cite your Supporting Information as outlined here: https://journals.plos.org/plosmedicine/s/supporting-information

REFERENCES

*PLOS uses the numbered citation (citation-sequence) method and first six authors, et al.

*Please ensure that journal name abbreviations match those found in the National Center for Biotechnology Information (NCBI) databases (http://www.ncbi.nlm.nih.gov/nlmcatalog/journals), and are appropriately formatted and capitalised.

*Where website addresses are cited, please include the complete URL and specify the date of access (e.g. [accessed: 12/06/2023]).

*Please also see https://journals.plos.org/plosmedicine/s/submission-guidelines#loc-references for further details on reference formatting.

MODELLING STUDIES

The following list is derived from Geoffrey P Garnett, Simon Cousens, Timothy B Hallett, Richard Steketee, Neff Walker. Mathematical models in the evaluation of health programmes. (2011) Lancet DOI:10.1016/S0140-6736(10)61505-X:

*If pertinent, please provide a diagram that shows the model structure, including how the natural history of the disease is represented, the process and determinants of disease acquisition, and how the putative intervention could affect the system.

*Please provide a complete list of model parameters, including clear and precise descriptions of the meaning of each parameter, together with the values or ranges for each, with justification or the primary source cited and important caveats about the use of these values noted.

*Please provide a clear statement about how the model was fitted to the data, including goodness-of-fit measure, the numerical algorithm used, which parameter varied, constraints imposed on parameter values, and starting conditions.

*For uncertainty analyses, please state the sources of uncertainties quantified and not quantified [can include parameter, data, and model structure].

*Please provide sensitivity analyses to identify which parameter values are most important in the model. Uncertainty estimates seek to derive a range of credible results on the basis of an exploration of the range of reasonable parameter values. The choice of method should be presented and justified.

*Please discuss the scientific rationale for the choice of model structure and identify points where this choice could influence conclusions drawn. Please also describe the strength of the scientific basis underlying the key model assumptions.

*For studies that develop a prediction model or evaluate its performance, please ensure that the study is reported according to the TRIPOD statement (https://www.equator-network.org/reporting-guidelines/tripod-statement) and include the completed checklist as Supporting Information. Please add the following statement, or similar, to the Methods: "This study is reported as per the Transparent Reporting of a Multivariable Prediction Model for Individual Prognosis Or Diagnosis (TRIPOD) statement (S1 Checklist)." For studies using machine learning, please use the TRIPOD-AI checklist. When completing the checklist, please use section and paragraph numbers, rather than page numbers.

# Author comment: Author response to Decision Letter 2

**DOI:** 10.1371/journal.pmed.1004819.r003

7 Feb 2026

Attachment Submitted filename: 20260131_PLOS Medicine_Response to Reviewers_Clean.docx

# Peer review: Decision Letter 2

**DOI:** 10.1371/journal.pmed.1004819.r004

**Authors:** Andreia Cunha, Andreia Cunha, Andreia Cunha

25 Mar 2026

Dear Dr Tozan,

Sincere apologies for the delay in getting back to you with a decision, which was due to challenges in securing all the necessary advice. Many thanks for submitting your manuscript "The Effect of Armed Conflict on Global Measles Cases: A Structural Equation Modeling Analysis of 193 Countries from 2000 to 2023" (PMEDICINE-D-25-03848R2) to PLOS Medicine. The paper has been reviewed by subject experts and a statistician; their comments are included below and can also be accessed here: [LINK]

As you will see, the reviewers are mostly satisfied with the revisions but raise some remaining concerns that we must ask you to address in full. After discussing the paper with the editorial team and an academic editor with relevant expertise, I'm pleased to invite you to revise the paper in response to the reviewers' comments. We plan to send the revised paper to some or all of the original reviewers, and we cannot provide any guarantees at this stage regarding publication.

In addition to these revisions, you may need to complete some formatting changes, which you will receive in a follow up email. A member of our team will be in touch with a set of requests shortly. If you do not receive a separate email within a few days, please assume that checks have been completed, and no additional changes are required.

When you upload your revision, please include a point-by-point response that addresses all of the reviewer and editorial points, indicating the changes made in the manuscript and either an excerpt of the revised text or the location (eg: page and line number) where each change can be found. Please also be sure to check the general editorial comments at the end of this letter and include these in your point-by-point response. When you resubmit your paper, please include a clean version of the paper as the main article file and a version with changes tracked as a marked-up manuscript. It may also be helpful to check the guidelines for revised papers at http://journals.plos.org/plosmedicine/s/revising-your-manuscript for any that apply to your paper.

We ask that you submit your revision by Apr 15 2026 11:59PM. However, if this deadline is not feasible, please contact me by email, and we can discuss a suitable alternative.

Don't hesitate to contact me directly with any questions (acunha@plos.org).

Best regards,

Andreia

Andreia Cunha, PhD

Senior editor

PLOS Medicine

acunha@plos.org

-----------------------------------------------------------

Comments from the reviewers:

Reviewer #1: Overall, the authors have addressed my major methodological and statistical concerns in a thorough and constructive manner. In particular, they have clarified the rationale for using SEM as an exploratory, theory-informed framework; substantially strengthened the discussion of causal assumptions and limitations; implemented multiple robustness analyses (including AR(1) specifications, lag structures, first-difference models, alternative transformations, multiple imputation, and clustering adjustments); and appropriately qualified interpretation in light of model fit indices and ecological design constraints.

I have one remaining minor clarification request. While vaccination coverage is incorporated as a mediator in several robustness models, the rationale for averaging across heterogeneous vaccine types could be explained more explicitly in the Methods section, including why this aggregation is appropriate for the conceptual framework and whether alternative specifications (e.g., measles-specific coverage only) were considered. This does not materially affect the main conclusions but would improve clarity.

Reviewer #2: The manuscript has been substantially strengthened in revision, and I appreciate the authors' careful engagement with prior methodological concerns. I have only a few additional points for clarification that I believe would further enhance the rigor and interpretability of the study.

1.The analysis relies on annual panel data. Given that both conflict events and measles outbreaks may exhibit important short-term dynamics, it would be helpful if the authors could briefly discuss whether annual aggregation might obscure shorter-term conflict-disease interactions or introduce temporal aggregation bias.

2.The conflict, displacement, and measles datasets are likely derived from different reporting systems with potentially distinct time-stamping conventions. Could the authors clarify how temporal alignment across these sources was handled? A brief discussion of potential reporting lags, synchronization issues, or measurement timing discrepancies would be valuable, particularly given the lagged path structure in the SEM framework.

3.Structural equation models can sometimes be subject to model equivalence, where alternative path structures may fit the data comparably well. Beyond the reverse-path specification already examined, it would be useful if the authors could briefly comment on how they assessed the plausibility or relative uniqueness of their specified model structure.

Reviewer #3: This is an interesting study that tries to understand whether and how armed conflict contributes to measles burden worldwide using 23 years of country data and an SEM framework. It is an interesting and plausible premise, and the authors appropriately frame it as an exploratory or structural study and have appropriate caveats about causal inference. Some minor clarifications and corrections would be needed to prepare it for publication. The revision addresses the reviewer comments well

Specific comments and suggestions:

1) The abstract references displacement as the pathway to measles without clarifying it is mostly through its impact on socioeconomic development - could be rephrased to avoid reader confusion about the causative pathway

2) In Table 1, measles cases are attributed to World Bank as the source, but the methods reference WHO as the source (more likely and appropriate). Please clarify

3) Figure 2 is not legible at all due to low resolution.

4) Consider changing the title from "The effect.." to "the association" to be more consistent with the appropriately non-causal framing of the findings.

Any attachments provided with reviews can be seen via the following link: [LINK]

--------------------------------------------------------- ---

General editorial requests:

*We ask every co-author listed on the manuscript to fill in a contributing author statement, making sure to declare all competing interests. If any of the co-authors have not filled in the statement, we will remind them to do so when the paper is revised. If all statements are not completed in a timely fashion this could hold up the re-review process. If new competing interests are declared later in the revision process, this may also hold up the submission. Should there be a problem getting one of your co-authors to fill in a statement we will be in contact. Please do not add or remove authors without first discussing this with the handling editor. You can see our competing interests policy here: http://journals.plos.org/plosmedicine/s/competing-interests.

*Please upload any figures associated with your paper as individual TIF or EPS files with 300dpi resolution at resubmission; please read our figure guidelines for more information on our requirements: http://journals.plos.org/plosmedicine/s/figures. While revising your submission, we strongly recommend that you use PLOS's NAAS tool (https://ngplosjournals.pagemajik.ai/artanalysis) to test your figure files. NAAS can convert your figure files to the TIFF file type and meet basic requirements (such as print size, resolution), or provide you with a report on issues that do not meet our requirements and that NAAS cannot fix.

After uploading your figures to PLOS's NAAS tool - https://ngplosjournals.pagemajik.ai/artanalysis, NAAS will process the files provided and display the results in the "Uploaded Files" section of the page as the processing is complete.

If the uploaded figures meet our requirements (or NAAS is able to fix the files to meet our requirements), the figure will be marked as "fixed" above. If NAAS is unable to fix the files, a red "failed" label will appear above.

When NAAS has confirmed that the figure files meet our requirements, please download the file via the download option, and include these NAAS processed figure files when submitting your revised manuscript.

*We expect all researchers with submissions to PLOS in which author-generated code underpins the findings in the manuscript to make all author-generated code available without restrictions upon publication of the work. In cases where code is central to the manuscript, we may require the code to be made available as a condition of publication. Authors are responsible for ensuring that the code is reusable and well documented. Please make any custom code available, either as part of your data deposition or as a supplementary file. Please add a sentence to your data availability statement regarding any code used in the study, e.g. "The code used in the analysis is available from Github [URL] and archived in Zenodo [DOI link]" Please review our guidelines at https://journals.plos.org/plosmedicine/s/materials-software-and-code-sharing and ensure that your code is shared in a way that follows best practice and facilitates reproducibility and reuse. Because Github depositions can be readily changed or deleted, we encourage you to make a permanent DOI'd copy (e.g. in Zenodo) and provide the URL.

GENERAL EDITORIAL REQUESTS

* At this stage, we ask that you include a short, non-technical Author Summary of your research to make findings accessible to a wide audience that includes both scientists and non-scientists. The Author Summary should immediately follow the Abstract in your revised manuscript. This text is subject to editorial change and should be distinct from the scientific abstract. Ideally each sub-heading should contain 2-3 single sentence, concise bullet points containing the most salient points from your study. In the final bullet point of ‘What Do These Findings Mean?’ Please include the main limitations of the study in non-technical language.

Please see our author guidelines for more information: https://journals.plos.org/plosmedicine/s/revising-your-manuscript#loc-author-summary.

* Please confirm that your title complies with PLOS Medicine's style. Your title must be nondeclarative and not a question. It should begin with main concept if possible. "Effect of" should be used only if causality can be inferred, i.e., for an RCT. Please place the study design ("A randomized controlled trial," "A retrospective study," "A modelling study," etc.) in the subtitle (ie, after a colon).

* Please confirm that your abstract complies with our requirements, including format (three sections: Background, Methods and Findings, and Conclusions) and providing all the information relevant to this study type https://journals.plos.org/plosmedicine/s/submission-guidelines#loc-abstract

* In the last sentence of the Abstract Methods and Findings section, please describe the main limitation(s) of the study's methodology.

* In the abstract, please include the important dependent variables that are adjusted for in the analyses.

* Please ensure that the Introduction ends with a clear description of the study question or hypothesis.

* Please ensure that all abbreviations are defined at first use throughout the text.

* Please confirm that all numbers presented in the abstract are present and identical to numbers presented in the main manuscript text.

* Please review your text for claims of novelty or primacy (e.g. 'for the first time') and remove this language. In addition, please check that any use of statistical terms (such as trend or significant) are supported by the data, and if not please remove them.

* Statistical reporting: Please revise throughout the manuscript, including tables and figures.

- Please report statistical information as follows to improve clarity for the reader (95% CI [13,28]; p</=).

- Please separate upper and lower bounds with commas instead of hyphens as the latter can be confused with reporting of negative values.

- Please repeat statistical definitions (HR, CI etc.) for each set of parentheses.

FUNDING STATEMENT

* The funding statement should include: specific grant numbers, initials of authors who received each award, URLs to sponsors’ websites. Also, please state whether any sponsors or funders (other than the named authors) played any role in study design, data collection and analysis, the decision to publish, or preparation of the manuscript. If they had no role in the research, include this sentence: “The funders had no role in study design, data collection and analysis, decision to publish, or preparation of the manuscript.

* It appears that one or more study authors is affiliated with one or more of the agencies that funded the study. Thus, the statement “The funders had no role in study design, data collection and analysis, decision to publish, or preparation of the manuscript” does not apply. Please revise the Financial Disclosure accordingly, as in "[Author name] is [author's role] at [funding agency]. The funders had no other role in study design…..”

COMPETING INTERESTS STATEMENT

* All authors must declare their relevant competing interests per the PLOS policy, which can be seen here: https://journals.plos.org/plosmedicine/s/competing-interests For authors with ties to industry, please indicate whether any of the interests has a financial stake in the results of the current study.

* Please add this statement to the manuscript's Competing Interests: "[Initials] is an Academic Editor on PLOS Medicine's editorial board.

DATA AVAILABILITY

* PLOS Medicine requires that the de-identified data underlying the specific results in a published article be made available, without restrictions on access, in a public repository or as Supporting Information at the time of article publication, provided it is legal and ethical to do so. Please see the policy at

http://journals.plos.org/plosmedicine/s/data-availability

and FAQs at

http://journals.plos.org/plosmedicine/s/data-availability#loc-faqs-for-data-policy

* The Data Availability Statement (DAS) requires revision. For each data source used in your study:

a) If the data are freely or publicly available, note this and state the location of the data: within the paper, in Supporting Information files, or in a public repository (include the DOI or accession number).

b) If the data are owned by a third party but freely available upon request, please note this and state the owner of the data set and contact information for data requests (web or email address). Note that a study author cannot be the contact person for the data.

c) If the data are not freely available, please describe briefly the ethical, legal, or contractual restriction that prevents you from sharing it. Please also include an appropriate contact (web or email address) for inquiries (again, this cannot be a study author).

* For studies in which a novel model is central to the manuscript's findings, as is the case here, authors are

responsible for providing the source code needed to replicate the study's findings in a repository (such as GitHub, SourceForge or Bitbucket) or a cloud computing service (such as Code Ocean). Protection of authors’ intellectual property will not be cause for exception. Please explain in the manuscript’s Data Availability Statement how readers can access the shared code.

FIGURES

* Please provide titles and legends for all figures and tables (including those in Supporting Information files). Please define all acronyms used in each figure or table in its corresponding legend.

INCLUSIVITY IN GLOBAL RESEARCH

PLOS has a 'Inclusivity in Global Research' policy which aims to promote collaboration and inclusivity in global health research. You are required to complete PLOS’ questionnaire on inclusivity in global research and submit it with your revised paper. The policy and questionnaire can be found at https://journals.plos.org/plosone/s/best-practices-in-research-reporting.

MODELING STUDIES

For more details on the requests below please see Geoffrey P Garnett, Simon Cousens, Timothy B Hallett, Richard Steketee, Neff Walker. Mathematical models in the evaluation of health programmes. (2011) Lancet DOI:10.1016/S0140-6736(10)61505-X.

* Please provide a diagram that shows the model structure, including how the disease natural history is represented, the process and determinants of disease acquisition, and how the putative intervention could affect the system.

* Please provide a complete list of model parameters, including clear and precise descriptions of the meaning of each parameter, together with the values or ranges for each, with justification or the primary source cited, and important caveats about the use of these values noted.

* Please provide a clear statement about how the model was fitted to the data including where relevant goodness-of-fit measure, the numerical algorithm used, which parameter varied, constraints imposed on parameter values, and starting conditions.

* For uncertainty analyses, please state the sources of uncertainties quantified and not quantified this can include parameter, data, and model structure.

* Please provide sensitivity analyses to identify which parameter values are most important in the model. Uncertainty estimates seek to derive a range of credible results on the basis of an exploration of the range of reasonable parameter values. The choice of method should be presented and justified.

* Please discuss the scientific rationale for this choice of model structure and identify points where this choice could influence conclusions drawn. Please also describe the strength of the scientific basis underlying the key model assumptions.

# Author comment: Author response to Decision Letter 3

**DOI:** 10.1371/journal.pmed.1004819.r005

21 Apr 2026

Attachment Submitted filename: 20260419_PLOS Medicine_Response to Reviewers_Clean.docx

# Peer review: Decision Letter 3

**DOI:** 10.1371/journal.pmed.1004819.r006

**Authors:** Andreia Cunha, Andreia Cunha, Andreia Cunha, Andreia Cunha

19 May 2026

Dear Dr. Tozan,

Thank you very much for re-submitting your manuscript "Association of armed conflict and global measles cases: A structural equation modeling analysis of 193 countries from 2000 to 2023" (PMEDICINE-D-25-03848R3) for review by PLOS Medicine.

I have discussed the paper with my colleagues and the academic editor and it was also seen again by the original reviewers. Two of the Reviewers have left no comments for authors but advised the editorial team they were happy with the revision. I am pleased to say that provided the remaining editorial and production issues are dealt with we are planning to accept the paper for publication in the journal.

The remaining issues that need to be addressed are listed at the end of this email. Any accompanying reviewer attachments can be seen via the link below. Please take these into account before resubmitting your manuscript:

[LINK]

***Please note while forming your response, if your article is accepted, you may have the opportunity to make the peer review history publicly available. The record will include editor decision letters (with reviews) and your responses to reviewer comments. If eligible, we will contact you to opt in or out.***

In revising the manuscript for further consideration here, please ensure you address the specific points made by each reviewer and the editors. In your rebuttal letter you should indicate your response to the reviewers' and editors' comments and the changes you have made in the manuscript. Please submit a clean version of the paper as the main article file. A version with changes marked must also be uploaded as a marked up manuscript file.

Please also check the guidelines for revised papers at http://journals.plos.org/plosmedicine/s/revising-your-manuscript for any that apply to your paper. If you haven't already, we ask that you provide a short, non-technical Author Summary of your research to make findings accessible to a wide audience that includes both scientists and non-scientists. The Author Summary should immediately follow the Abstract in your revised manuscript. This text is subject to editorial change and should be distinct from the scientific abstract.

In addition to these revisions, you may need to complete some formatting changes, which you will receive in a follow up email. A member of our team will be in touch with a set of requests shortly. If you do not receive a separate email within a few days, please assume that checks have been completed, and no additional changes are required.

We expect to receive your revised manuscript within 1 week. Please email us (plosmedicine@plos.org) if you have any questions or concerns.

We ask every co-author listed on the manuscript to fill in a contributing author statement. If any of the co-authors have not filled in the statement, we will remind them to do so when the paper is revised. If all statements are not completed in a timely fashion this could hold up the re-review process. Should there be a problem getting one of your co-authors to fill in a statement we will be in contact. YOU MUST NOT ADD OR REMOVE AUTHORS UNLESS YOU HAVE ALERTED THE EDITOR HANDLING THE MANUSCRIPT TO THE CHANGE AND THEY SPECIFICALLY HAVE AGREED TO IT.

Please ensure that the paper adheres to the PLOS Data Availability Policy (see http://journals.plos.org/plosmedicine/s/data-availability), which requires that all data underlying the study's findings be provided in a repository or as Supporting Information. For data residing with a third party, authors are required to provide instructions with contact information for obtaining the data. PLOS journals do not allow statements supported by "data not shown" or "unpublished results." For such statements, authors must provide supporting data or cite public sources that include it.

To enhance the reproducibility of your results, we recommend that you deposit your laboratory protocols in protocols.io, where a protocol can be assigned its own identifier (DOI) such that it can be cited independently in the future. Additionally, PLOS ONE offers an option to publish peer-reviewed clinical study protocols. Read more information on sharing protocols at https://plos.org/protocols?utm_medium=editorial-email&utm_source=authorletters&utm_campaign=protocols

Please review your reference list to ensure that it is complete and correct. If you have cited papers that have been retracted, please include the rationale for doing so in the manuscript text, or remove these references and replace them with relevant current references. Any changes to the reference list should be mentioned in the rebuttal letter that accompanies your revised manuscript.

Please note, when your manuscript is accepted, an uncorrected proof of your manuscript will be published online ahead of the final version, unless you've already opted out via the online submission form. If, for any reason, you do not want an earlier version of your manuscript published online or are unsure if you have already indicated as such, please let the journal staff know immediately at plosmedicine@plos.org.

If you have any questions in the meantime, please contact me or the journal staff on plosmedicine@plos.org.

We look forward to receiving the revised manuscript by May 26 2026 11:59PM.

Sincerely,

Andreia Cunha, PhD

Senior Editor

PLOS Medicine

plosmedicine.org

------------------------------------------------------------

Requests from Editors:

GENERAL

* Please disclose any use of AI tools and technologies to the study or article content in the Methods, including the name(s) of any tool(s) used and a description of their use. Please see our guidelines for more information: https://journals.plos.org/plosmedicine/s/ethical-publishing-practice#loc-artificial-intelligence-tools-and-technologies.

* Please review your text for claims of novelty or primacy (e.g. 'novel') and remove this language.

CODE AVAILABILITY

* Please ensure that your code is shared in a way that follows best practice and facilitates reproducibility and reuse. Because Github depositions can be readily changed or deleted, we request that you to make a permanent DOI'd copy (e.g. in Zenodo) and provide the URL in the Data Availability statement.

FIGURES

* When a p value is given, please specify the statistical test used to determine it in the legend.

* Please provide the unadjusted comparisons as well as the adjusted comparisons in all relevant Tables

* Please specify the variables controlled for in all relevant Tables

ACKNOWLEDGMENTS

* Please include an Acknowledgments section in your manuscript.

OBSERVATIONAL, COHORT, CROSS-SECTIONAL, AND CASE CONTROL STUDIES

*Please ensure that the study is reported according to the STROBE guideline, and include the completed STROBE checklist as Supporting Information. Please add the following statement, or similar, to the Methods: ""This study is reported as per the Strengthening the Reporting of Observational Studies in Epidemiology (STROBE) guideline (S1 Checklist).""

The STROBE guideline can be found here: http://www.equator-network.org/reporting-guidelines/strobe/

When completing the checklist, please use section and paragraph numbers, rather than page numbers.

* Did your study have a prospective protocol or analysis plan? Please state this (either way) early in the Methods section.

a) If a prospective analysis plan (from your funding proposal, IRB or other ethics committee submission, study protocol, or other planning document written before analyzing the data) was used in designing the study, please include the relevant prospectively written document with your revised manuscript as a Supporting Information file to be published alongside your study, and cite it in the Methods section. A legend for this file should be included at the end of your manuscript.

b) If no such document exists, please make sure that the Methods section transparently describes when analyses were planned, and when/why any data-driven changes to analyses took place.

c) In either case, changes in the analysis-- including those made in response to peer review comments-- should be identified as such in the Methods section of the paper, with rationale.

* Your study is observational and therefore causality cannot be inferred. Please remove language that implies causality and refer to associations instead.

* For all observational studies, in the manuscript text, please indicate: (1) the specific hypotheses you intended to test, (2) the analytical methods by which you planned to test them, (3) the analyses you actually performed, and (4) when reported analyses differ from those that were planned, transparent explanations for differences that affect the reliability of the study's results. If a reported analysis was performed based on an interesting but unanticipated pattern in the data, please be clear that the analysis was data-driven.

Comments from Reviewers:

Reviewer #2: The authors have addressed most of my comments properly. The remaining issues that have not been revised are now appropriately discussed in the limitations section. I am generally satisfied with the revised manuscript and recommend acceptance.

Any attachments provided with reviews can be seen via the following link:

[LINK]

# Author comment: Author response to Decision Letter 4

**DOI:** 10.1371/journal.pmed.1004819.r007

30 May 2026

Attachment Submitted filename: 20260530_Response to Editor_Final.docx

# Editor report: Decision Letter 4

**DOI:** 10.1371/journal.pmed.1004819.r008

**Authors:** Andreia Cunha, Andreia Cunha, Andreia Cunha, Andreia Cunha, Andreia Cunha

2 Jun 2026

Dear Dr Tozan,

On behalf of my colleagues and the Academic Editor, Dr Zulfiqar A. Bhutta, I am pleased to inform you that we have agreed to publish your manuscript "Association of armed conflict and global measles cases: A structural equation modeling analysis of 193 countries from 2000 to 2023" (PMEDICINE-D-25-03848R4) in PLOS Medicine.

Before your manuscript can be formally accepted you will need to complete some formatting changes, which you will receive in a follow up email. Please be aware that it may take several days for you to receive this email; during this time no action is required by you. Once you have received these formatting requests, please note that your manuscript will not be scheduled for publication until you have made the required changes.

In the meantime, please log into Editorial Manager at http://www.editorialmanager.com/pmedicine/, click the "Update My Information" link at the top of the page, and update your user information to ensure an efficient production process.

PRESS

We frequently collaborate with press offices. If your institution or institutions have a press office, please notify them about your upcoming paper at this point, to enable them to help maximise its impact. If the press office is planning to promote your findings, we would be grateful if they could coordinate with medicinepress@plos.org. If you have not yet opted out of the early version process, we ask that you notify us immediately of any press plans so that we may do so on your behalf.

We also ask that you take this opportunity to read our Embargo Policy regarding the discussion, promotion and media coverage of work that is yet to be published by PLOS. As your manuscript is not yet published, it is bound by the conditions of our Embargo Policy. Please be aware that this policy is in place both to ensure that any press coverage of your article is fully substantiated and to provide a direct link between such coverage and the published work. For full details of our Embargo Policy, please visit http://www.plos.org/about/media-inquiries/embargo-policy/.

To enhance the reproducibility of your results, we recommend that you deposit your laboratory protocols in protocols.io, where a protocol can be assigned its own identifier (DOI) such that it can be cited independently in the future. Additionally, PLOS ONE offers an option to publish peer-reviewed clinical study protocols. Read more information on sharing protocols at https://plos.org/protocols?utm_medium=editorial-email&utm_source=authorletters&utm_campaign=protocols

Thank you again for submitting to PLOS Medicine. We look forward to publishing your paper.

Sincerely,

Andreia Cunha, PhD

Senior Editor

PLOS Medicine
